# Age- and sex-related profiles for macro, macro/micro and microvascular reactivity indexes: Association between indexes and normative data from 2609 healthy subjects (3-85 years)

**DOI:** 10.1371/journal.pone.0254869

**Published:** 2021-07-19

**Authors:** Yanina Zócalo, Daniel Bia

**Affiliations:** Departamento de Fisiología, Facultad de Medicina, Centro Universitario de Investigación, Innovación y Diagnóstico Arterial (CUiiDARTE), Universidad de la República, Montevideo, Uruguay; Oregon State University, UNITED STATES

## Abstract

Vascular reactivity (VR), defined as blood vessels’ capability to actively modify the diameter and flow resistances can be non-invasively assessed analyzing vascular response to forearm occlusion. Several VR indexes can be quantified: (i) ´microvascular´, which consider variables that depend almost exclusively on changes in distal resistances, (ii)´ macrovascular´, that evaluate the changes in brachial artery (BA) diameter, adjusting for blood flow stimulus, and (iii) ´macro/micro´, whose values depend on the micro and macrovascular response without discriminating each one´s contribution. VR indexes could not be associated. Many VR indexes have been used without availability of adequate normative data (reference intervals, RIs). Aims: (1) to evaluate macro, macro/micro and micro VR indexes obtained in a cohort of healthy children, adolescents and adults, (2) to evaluate the association between VR indexes, (3) to determine the need for age and/or sex-specific RIs, and (4) to define RIs for VR indexes. Methods: Ultrasound (B-mode/Doppler) and automatic computerized analysis were used to assess BA diameter, blood flow velocity and distal resistances, at rest and in conditions of decreased and increased blood flow. Macro, macro/micro and micro VR indexes were quantified (n = 3619). RIs-subgroups were defined according to European Reference Values for Arterial Measurements Collaboration Group (n = 1688, 3–84 years) and HUNT3-Fitness Study Group (n = 2609, 3–85 years) criteria. Mean value and standard deviation equations were obtained for VR indexes. The need for age or sex-specific RIs was analyzed. Percentile curves were defined and data were compared with those obtained in other populations. Conclusion: Macro and macro/micro VR indexes showed no association (or it was very weak) with microvascular indexes. Age- and sex-related profiles and RIs for macro, macro/micro and micro VR indexes were defined in a large population of healthy subjects (3–85 y). Equations for mean, standard deviation and percentiles values (year-to-year) were included in text and spreadsheet formats.

## Introduction

Vascular reactivity (VR) defined as blood vessels’ capability to actively modify the diameter and resistance in response to a stimulus (e.g., blood flow changes) can be non-invasively assessed following post-ischemic vascular response to forearm occlusion [1–5]. Schematically, distal microvessels are stimulated during inflation of a pressure cuff. The occlusion causes transient ischemia, which stimulates dilation of distal resistance microvessels [5]. The high flow velocity, perfusion and/or the reduced resistances observed after cuff deflation (during reactive hyperemia (RH)) are considered ´microvascular reactivity indexes´ [2–6]. In turn, diameter changes in the conduit artery (brachial artery, BA) before (e.g., low flow-mediated constriction) and after (e.g., flow-mediated dilation (FMD)) cuff-deflation are considered macrovascular reactivity indexes [2]. However, it should be noted that changes in conduit artery diameter depend on the arterial intrinsic capability to actively modify its diameter (e.g., liberation of endothelial vasoactive factors, smooth muscle response-capacity), as well as on the RH stimulus related with the microvascular response [2]. An altered microvascular reactivity could associate a reduced forearm blood flow during RH (a reduced stimulus of flow) which might contribute to a reduced macrovascular response. Then, ´unadjusted macrovascular reactivity indexes´ would reflect both macro and microvascular response (´macro/micro VR indexes´) [2,4,5,7]. On the other hand, macrovascular reactivity indexes adjusted for RH stimulus would enable to assess macrovascular response in isolation [2,4,5,7]. In this context, it should be noted that Dhindsa et al. (2008) described that ´micro´, ´macro/micro´ and ´macro´ VR responses could not be associated [4]. Thus, different physiological mechanisms would be involved in the stimulation and development of micro and macrovascular responses [4]. Furthermore, it was recently suggested that aging-related changes in VR, could be observed when assessed in conduit arteries (e.g., FMD), but not in the microcirculation [8]. Consequently, macro and microvascular reactivity physiological changes during growth, development and aging could differ. The characterization of expected values for VR indexes and their age-related changes is of value in the field of vascular biology and research. However, to our knowledge there is scarce data about age-related physiological profiles for VR indexes.

VR assessment has been considered useful in early detection of cardiovascular (CV) disease and risk stratification, which accounts for the interest in VR evaluation in clinical practice [2,9–12]. However macro, macro/micro and micro VR indexes have been used without availability of adequate normative data (age and sex-related reference intervals or percentiles, RIs), obtained in large populations of children, adolescents and adults. The few studies aimed at defining RIs or cutoff values (i) quantified one or two VR indexes (mainly FMD) [13,14]; (ii) considered specific life stages or age-groups [13–19]; (iii) defined normative data for wide age-range groups (e.g., cut-off points per decade) [13,17] or (iv) used approaches currently not recommended (e.g., manual edge detection, single or specific time measures during RH, evaluation of a short time period after cuff deflation) [13,14]. Factors related with the above could have limited VR measurement in clinical practice.

This work’s aims were: (1) to evaluate macro, macro/micro and micro VR indexes obtained in a cohort of healthy children, adolescents and adults from South America, (2) to evaluate the association between indexes used to assess VR, (3) to determine the need for age and/or sex-specific RIs (normative data), and (4) to define RIs for the different VR indexes.

## Materials and methods

### Study population

The study was carried out in the context of the Centro Universitario de Investigación, Innovación y Diagnóstico Arterial (CUiiDARTE) project [20–27], a population-based study developed in Uruguay. In this work, we considered data from 3619 subjects included in the CUiiDARTE Database. This includes data on demographic and anthropometric variables, exposure to CV risk factors (CRFs), personal and family history of CV disease (CVD) and data on structural and functional CV parameters, obtained non-invasively, mainly from community-based projects. All procedures agreed with the Declaration of Helsinki (1975; reviewed in 1983). The study protocol was approved by Institutional Ethic Committee (Comité de Ética en Investigación del Centro Hospitalario Pereira-Rossell). Written informed consent was obtained from participants or from parents in case of subjects aged <18 years old (y), who gave informed assent before data collection. Subjects or parents (in case of subjects aged <18 y) also provided informed written consent to have data from their medical records used in research.

### Anthropometric and clinical evaluation

A clinical interview, together with the anthropometric evaluation enabled us to assess CRFs exposure. A family history of CVD was defined by the presence of first-degree (for all the subjects) and/or second-degree (for subjects ≤18 y) relatives with early (<55 y in males; <65 y in females) CVD. Body weight (BW) and height (BH) were measured with the participant wearing light clothing and no shoes. Standing BH was measured using a portable stadiometer and recorded to the nearest 0.1 cm. BW was measured with an electronic scale (841/843, Seca Inc., Hamburg, Germany; model HBF-514C, Omron Inc., Chicago, Illinois, USA) and recorded to the nearest 0.1 kg. Body mass index (BMI) was calculated as BW-to-squared BH ratio. In children and adolescents z-scores for the BMI were calculated using the World Health Organization software (Anthro-v.3.2.2; Anthro-Plus-v.1.0.4).

### Cardiovascular evaluation

Participants were asked to avoid exercise, tobacco, alcohol, caffeine and food-intake four hours before the evaluation. All haemodynamic measurements were performed in a temperature-controlled environment (21–23˚C), with the subject in supine position and after resting for at least 10–15 minutes, which enabled reaching steady hemodynamic conditions. Using a validated oscillometric device (HEM-433INT; Omron Healthcare Inc., Lake Forest, Illinois, USA), heart rate (HR), and brachial systolic and diastolic blood pressure (BP) levels (bSBP and bDBP) were recorded in supine position simultaneously and/or immediately before or after each VR recording. Then, brachial pulse pressure (bPP; bPP = bSBP–bDBP) and mean BP (bMBP, bMBP = bDBP+bPP/3) were obtained.

CV evaluation in CUiiDARTE project includes assessing: (i) peripheral (brachial, radial, tibial) and central (aortic, carotid) BP levels; central (aortic, carotid) pulse wave analysis and wave separation analysis-derived parameters (e.g., augmentation index, forward and backward pressure wave components), (ii) carotid, femoral and BA diameter waveforms and intima-media thickness, (iii) VR indexes, (iv) carotid, femoral and BA Doppler-derived blood velocity profiles and indexes (e.g., resistive), (v) ankle-brachial index, (vi) screening for carotid and femoral atherosclerotic plaques, (vii) carotid, femoral and BA local stiffness (e.g., elastic modulus), (viii) hemodynamic evaluation (e.g., systemic resistances, cardiac output) using BA pulse contour analysis and/or impedance cardiography, and (ix) regional stiffness (e.g., carotid-femoral, carotid-radial pulse wave velocity). In this work, the analysis was focused on VR data [22,23].

### Carotid and femoral artery ultrasound

Left and right common, internal and external carotid arteries, vertebral arteries and common femoral arteries were examined (B-Mode and Doppler ultrasound, 7–13 MHz, linear transducer, M-Turbo, SonoSite Inc., Bothell, WA, USA) [26,27]. Transverse and longitudinal views (from different angles) were obtained to assess atherosclerotic plaques presence. An atherosclerotic plaque was defined as focal wall thickening at least 50% greater than adjacent segments; focal thickening protruding into the lumen at least 0.5 mm and/or an intima-media thickness ≥1.5 mm [26,27].

### Vascular reactivity assessment

**Fig 1:**
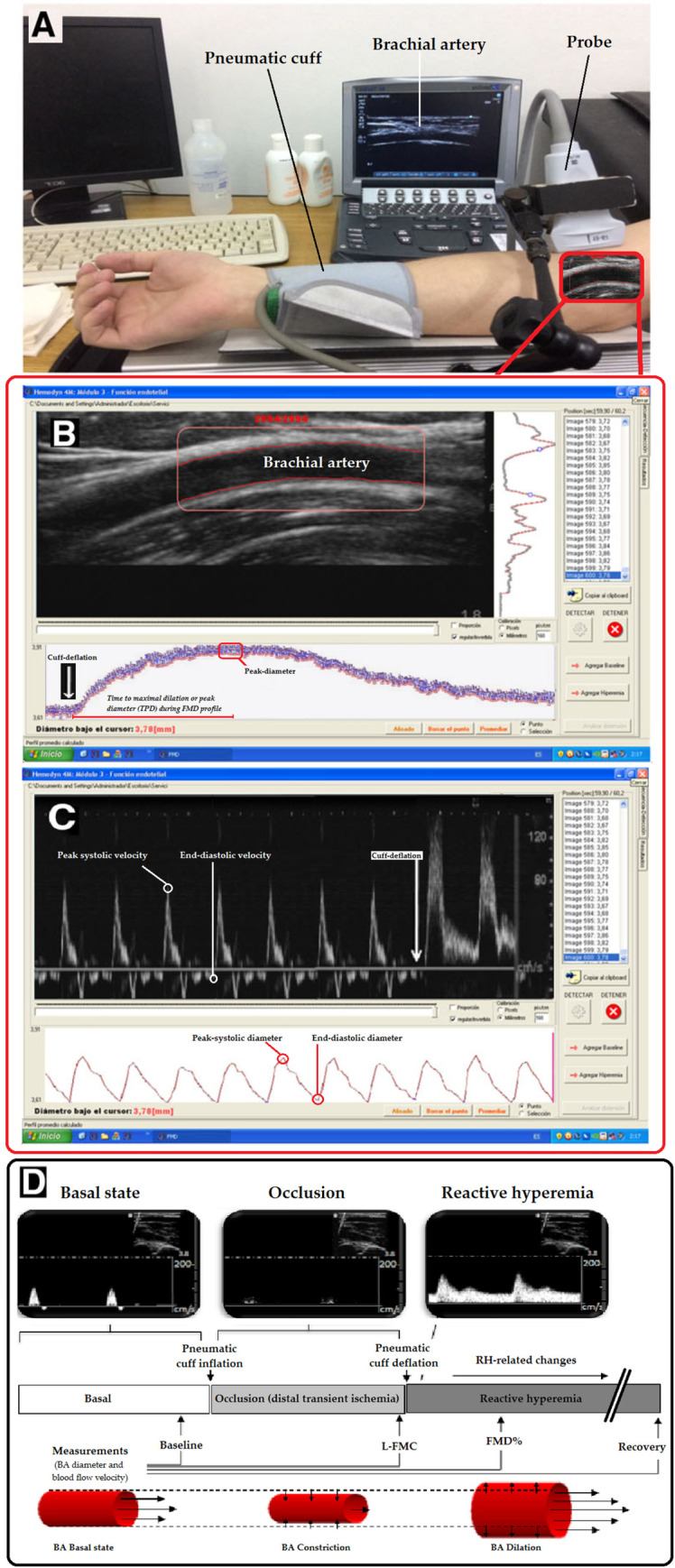
Vascular reactivity assessment. A: Instrumentation for vascular reactivity (VR) evaluation. B: Brachial artery (BA) characteristic image (top) with software tracking vessel wall over time (edge detection analysis) in the region of interest (ROI), so as to reconstruct beat-to-beat BA diameter temporal profile (bottom), before and after cuff-deflation (release of arterial occlusion). Note the beat-to-beat BA dilation after cuff-deflation. Data in the figure were obtained in a healthy subject. Blood velocity values and systolic, mean and end-diastolic diameters were determined for each analyzed beat. C: BA blood flow velocity and diameter at the time of cuff deflation (white arrow). D: Basal and VR (e.g., FMD%) data were assessed following a protocol that included 60-second (s) records in basal conditions, 300 s during arterial occlusion (cuff inflation) and 240 s after cuff deflation (reactive hyperemia, RH).

Vascular reactivity was evaluated by means of standardized methods [2,5,12] [Fig 1].

Left shoulder and arm were positioned on a support, ensuring comfort and stability, thus avoiding muscle tension development and subsequent movement. In turn, forearm and wrist were placed on a support to minimize motion and artifacts in the records (e.g. due to cuff inflation). Then, BA was interrogated in longitudinal plane (7–13 MHz, M-Turbo, SonoSite Inc., USA). To ensure adequate records the transducer was fixed using a stereotactic probe holder [2,3]. Doppler and B-modes were selected to record BA center-line blood flow sonogram and diameter. A standard pediatric BP-cuff (Omron, Japan), positioned distally in the forearm, was inflated to 50 mmHg above bSBP for 5 minutes. Ultrasound-derived image sequences (videos) were obtained in the following conditions: (i) baseline (60-second (s) videos obtained immediately before cuff-inflation), (ii) occlusion (300-s videos recorded during the time the cuff remained inflated (distal transient ischemia) and (iii) release (240-s videos recorded during the cuff-deflation or release and subsequent RH) [2,3]. An examination takes less than 30 minutes, including preparation, rest and scan.

Recorded videos were stored for blinded off-line analysis. Automatic wall-detection and Doppler velocity tracing software (Hemodyn-4-M, Dinap s.r.l, Argentina; Sonosite Inc, USA) were used [3,28]. Once a straight segment was identified in the BA (B-mode recording) and defined as the region of interest (ROI), the software allowed beat-to-beat automatic identification of arterial wall-lumen interfaces. Then, distances between anterior and posterior walls, averaged over the ROI, were the estimate for instantaneous BA diameter. Instantaneous blood flow velocity was obtained. Instantaneous (beat-to-beat) arterial diameter and blood velocity were measured before, during and after arterial occlusion (´baseline´, ´occlusion´ and ´release or RH videos´, respectively).

The beginning and end of the arterial occlusion were identified on the flow velocity signal. That made it easier to assess the vascular parameters in the different conditions (e.g., basal, RH): (i) peak systolic, mean and end-diastolic diameter (DD, mm), (ii) peak systolic (PSV, cm/s), mean and end-diastolic flow velocity (EDV, cm/s), and (iii) resistive index (RI), a measure of pulsate flow that reflects the resistance associated with distal microvessels [3]. RI was quantified as:

$$RI=\frac{PSV-EDV}{PSV}$$ (1)

Baseline diameter and flow velocity were calculated as the mean of data obtained in baseline conditions. Values corresponding to RH state were maximum levels observed within the first 210 s after occlusion release (cuff-deflation). Pre-release arterial diameter and flow velocity were calculated as the mean of data acquired in the last 15–30 s before cuff-deflation. Time to peak DD was calculated as the time from cuff-deflation to maximum hyperemic DD [3]. Considering a given blood viscosity (μ, μ = 0.035 dyne*s/cm^2^) and a parabolic velocity profile, wall shear stress (WSS) can be obtained as a function of local centre-stream velocity (V, cm/s) and diameter (D, cm) [29]:

$$WSS=\frac{\left(8.\mu .V\right)}{D}$$ (2)

From Eq 2 peak systolic and end-diastolic WSS were calculated.

### Vascular reactivity indexes

Similar to Dhindsa et al., in this work VR indexes were schematically divided into: (i) ´microvascular indexes´, which consider variables that depend almost exclusively on changes in distal resistances (e.g., RH indexes), (ii) ´macrovascular indexes´, that evaluate the change in the diameter of the BA, adjusting for blood flow velocity or WSS (e.g., FMD/WSS) and (iii) ’macro/micro´ VR indexes, whose values depend on the micro and/or the macrovascular response without discriminating each one´s relative contribution (e.g., WSS change, due diameter and/or blood flow change) [4,5].

### Macro and macro/micro vascular reactivity indexes

These indexes can be divided into those that: (i) evaluate BA response by comparing baseline conditions with those observed after cuff-deflation (RH state), (ii) evaluate BA response by comparing baseline data with those before cuff-deflation (Pre-release state), (iii) integrate the above responses. In addition, as was mentioned, some approaches analyze BA response (change in diameter) adjusted for the stimulus (macro VR indexes), whereas others do not (macro/micro VR indexes).

#### Basal vs. RH indexes (not adjusted for stimulus)

Celermajer et al. approach that involves VR test has become the most popular method to assess endothelial function (EF) [30]. As mentioned, a pneumatic cuff is positioned around the forearm and insuflated to determine transient (5 minutes) distal ischemia. Once the cuff is deflated, an increase in BA flow is observed, which stimulates the endothelium to release vasodilator factors. This results in BA dilatation, assessed by B-Mode ultrasound. The magnitude of BA dilation is used as EF or VR index. Over the years, the original approach has been modified (improved), for example, by includying in the analysis the time-course of the FMD, rather than considering discrete (punctual) times (e.g., 30, 60, 90 s) [2,3,12].

The vascular response (in absolute ☯mm] and relative ☯ %] terms), can be quantified as [12]:

$${\Delta DD}_{Peak\_Basal}={DD}_{Peak}-{DD}_{Basal}$$ (3)

$${DD\;Ratio}_{Peak\_Basal}=\frac{{DD}_{Peak}}{{DD}_{Basal}}*100$$ (4)

where DD_*Peak*_ and DD_*Basal*_ are maximum end-diastolic (RH state) and baseline end-diastolic BA diameters, respectively [3,31]. FMD% was quantified as [31–33]:

$$FMD\%=\frac{{DD}_{Peak}-{DD}_{Basal}}{{DD}_{Basal}}*100$$ (5)

Different FMD% temporal-patterns, have been described, which show differences in the kinetics of the dilatory response [3,34–36]. The magnitude and kinetics (e.g., latency) of the response would give complimentary information. Subjects with a delayed though significant vasodilation, associated with a blunted early response, exhibit increased CV risk [35]. In this work we quantified the time to peak diamater (TPD), as the time to maximal end-diastolic diameter or maximal dilation after cuff-deflation (RH state) (TPD_FMD%).

#### Basal vs. RH indexes (adjusted for stimulus)

Different ways to include the stimulus in the analysis have been proposed. The way to determine the stimulus more accurately is still discussed [31]. Some of the most used indexes were considered in this work [3,31].


$${\Delta DD}_{Peak\_Basal}/{\Delta V}_{Peak\_Basal}=\frac{{DD}_{Peak}-{DD}_{Basal}}{{EDV}_{Peak}-{EDV}_{Basal}}$$ (6)

$$FMD/{\Delta V}_{Peak\_Basal}=\frac{\left(\frac{{DD}_{Peak}-{DD}_{Basal}}{{DD}_{Basal}}\right)}{{EDV}_{Peak}-{EDV}_{Basal}}$$ (7)

$$pFMDv=\frac{\left(\frac{{DD}_{Peak}-{DD}_{Basal}}{{DD}_{Basal}}\right)}{\left(\frac{{EDV}_{Peak}-{EDV}_{Basal}}{{EDV}_{Basal}}\right)}$$ (8)

FMD% was also normalized by WSS levels [3,7,37]:

$${FMD\%}_{WSS}=\frac{\left((\frac{{DD}_{Peak}-{DD}_{Basal}}{{DD}_{Basal}})*100\right)}{\left(\frac{{EDWSS}_{Peak}-{EDWSS}_{Basal}}{{EDWSS}_{Basal}}\right)}=\frac{FMD\%}{\left(\frac{{EDWSS}_{Peak}-{EDWSS}_{Basal}}{{EDWSS}_{Basal}}\right)}$$ (9)

#### Basal vs. pre-release indexes (not adjusted for stimulus)

$${\Delta DD}_{Prerelease\_Basal}={DD}_{Prerelease}-{DD}_{Basal}$$ (10)

$${DD\;Ratio}_{Prerelease\_Basal}=\frac{{DD}_{Prerelease}}{{DD}_{Basal}}*100$$ (11)

where DD_Prre-release_ is the BA diameter at the end of vascular occlussion (pre-release) [3,31].

Whereas FMD% provides data about EF ´recruitability´(e.g., response to a specific stimulus), it does not provide information related with basal or tonic EF (e.g., basal release of vasoctive factors) [38,39]. A low baseline tone, leading to pre-dilated BA, could result in a blunted FMD% despite of normal EF. In turn, high baseline tone, associated with pre-constricted BA, could result in normal FMD%, in spite of endothelial dysfunction. Therefore, FMD% does not provide data on the endothelial responsiveness to resting WSS levels, nor on the vasoconstrictor response to WSS reductions [38,39]. To assess the arterial response to low blood flow (vaso-constriction), Gori et al. proposed an index that considers data obtained during vascular occlusion [38]. Similar to FMD%, the response observed under conditions of reduced flow was named low-flow mediated (vaso) constriction (L-FMC) [38]. L-FMC% could provide (complementary) data useful in the characterization of vessel responsiveness and/or CV risk stratification [39]. In this work L-FMC was quantified as percentage change in BA DD, considering basal and pre-release data [28,29]:

$$LFMC\%=\frac{{DD}_{Prerelease}-{DD}_{Basal}}{{DD}_{Basal}}*100$$ (12)

#### Basal vs. pre-release indexes (adjusted for stimulus)

$${\Delta DD}_{Prerelease\_Basal}/{\Delta V}_{Prerelease\_Basal}=\frac{{DD}_{Prerelease}-{DD}_{Basal}}{{EDV}_{Prerelease}-{EDV}_{Basal}}$$ (13)

$$LFMC/{\Delta V}_{Prelease\_Basal}=\frac{\left(\frac{{DD}_{Prerelease}-{DD}_{Basal}}{{DD}_{Basal}}\right)}{{EDV}_{Prerelease}-{EDV}_{Basal}}$$ (14)

$$pLFMCv=\frac{\left(\frac{{DD}_{Prerelease}-{DD}_{Basal}}{{DD}_{Basal}}\right)}{\left(\frac{{EDV}_{Prerelease}-{EDV}_{Basal}}{{EDV}_{Basal}}\right)},$$ (15)

LFMC% normalized by WSS was also calculated:

$${LFMC\%}_{WSS}=\frac{LFMC\%}{\left(\frac{{EDWSS}_{Prerelease}-{EDWSS}_{Basal}}{{EDWSS}_{Basal}}\right)}$$ (16)

#### Total vasoactive range or total vascular reactivity

It was proposed that the combined evaluation of vasodilator and vasoconstrictor responses (FMD% and LFMC%), as well as their composite endpoint, the total vasoactive range or vascular reactivity (TVR) may improve CV risk stratification [15,38,39]. In this work TVR was quantified [15]:

$$TVR=\frac{{DD}_{Peak}-{DD}_{Prerelease}}{{DD}_{Basal}}*100$$ (17)

#### Hyperemic wall shear-stress

$${\Delta WSS}_{Peak\_Basal}={WSS}_{Peak}-{WSS}_{Basal}$$ (18)

### Microvascular reactivity indexes

Blood flow velocity and distal resistances changes (Doppler-derived) during RH have been proposed to evaluate microvascular reactivity [3,40].


$${\Delta V}_{Peak\_Basal}=V_{Peak}-V_{Basal}$$ (19)

$$\Delta RI={RI}_{Peak}-{RI}_{Basal}$$ (20)

$${\Delta RI\%}_{Peak\_Basal}=\frac{{RI}_{Peak}-{RI}_{Basal}}{{RI}_{Basal}}*100$$ (21)

Table 1 shows the different indexes considered to assess VR in the present work.

**Table 1:** Vascular reactivity indexes: Equations.

Index	Equation	
**1. Macrovascular and Macro/Microvascular Indexes**		
**1.1. Basal vs. Hyperemia Indexes (not adjusted for stimulus)**		
ΔDD_Peak_Basal_ [mm]		DD_Peak_—DD_Basal_
DD Ratio _Peak_Basal_ [%]		(DD_Peak_/DD_Basal_)*100
FMD% [%]		((DD_Peak_—DD_Basal_)/DD_Basal_)*100
TPD_FMD% [seconds]		Time between cuff deflation and hyperemic end-diastolic peak diameter
**1.2. Basal vs. Hyperemia Indexes (adjusted for stimulus)**		
ΔDD _Peak-Basal_/ΔV_Peak-Basal_ [mm/cm/s]		(DDPeak—DD_Basal_)/(EDV_Peak_- EDV_Basal_)
FMD/ΔV_Peak_Basal_ [1/cm/s]		((DD_Peak_ -DD_Basal_)/DD_Basal_)/(EDV_Peak_-EDV_Basal_)
pFMDv		(DD_Peak_-DD_Basal_)/DD_Basal_/((EDV_Peak_-EDV_Basal_)/EDV_Basal_)
FMD%_WSS_		(((DD_Peak_—DD_Basal_)/DD_Basal_)*100)/((EDWSS_Peak_—EDWSS_Basal_)/ED WSS_Basal_
**1.3. Basal vs. Pre-release Indexes (not adjusted for stimulus)**		
ΔDD_Prerelease_Basal_ [mm]		DD_Prerelease_—DD_Basal_
DD Ratio _Prerelease_Basal_ [%]		(DD_Prerelease_/DD_Basal_)*100
LFMC% [%]		(DD_Prerelease_ − DD_Basal_)/DD_Basal_)*100
**1.4. Basal vs. Pre-release Indexes (adjusted for stimulus)**		
ΔDD_Prerelease_Basal_/ΔV_Prelease_Basal_		(DDPrerelease—DD_Basal_)/(EDV_Prerelease_-EDV_Basal_)
LFMC/ΔV_Prerelease_Basal_ [1/cm/s]		((DDPrerelease—DD_Basal_)/DD_Basal_)/(EDVPrerelease—EDV_Basal_)
pL-FMCv		((DDPre-release—DD_Basal_)/DD_Basal)_/((EDVPrerelease—EDV_Basal_)/EDV_Basal_)
L-FMC%/WSS		L-FMC%/((EDWSSPrerelease—EDWSS_Basal_)/EDWSS_Basal_)
**1.5.Total Vascular Reactivity**		
TVR [%]		((DDPeak—DD_Prerelease_)/DD_Basal_)*100
**1.6. Hyperemic shear-stress**		
ΔWSS_Peak_Basal_ [dyn/cm^2^]		WSS_Peak_—WSS_Basal_
**3. Microvascular Indexes**		
ΔV_Peak_Basal_ [cm/s]		V_Peak_—V_Basal_
ΔRI_Peak_Basal_		RI_Peak_—RI_Basal_
ΔRI%_Peak_Basal_ [%]		((RI_Peak_—RI_Basal_)/RI_Basal_)*100

### Data analysis

**Fig 2:**
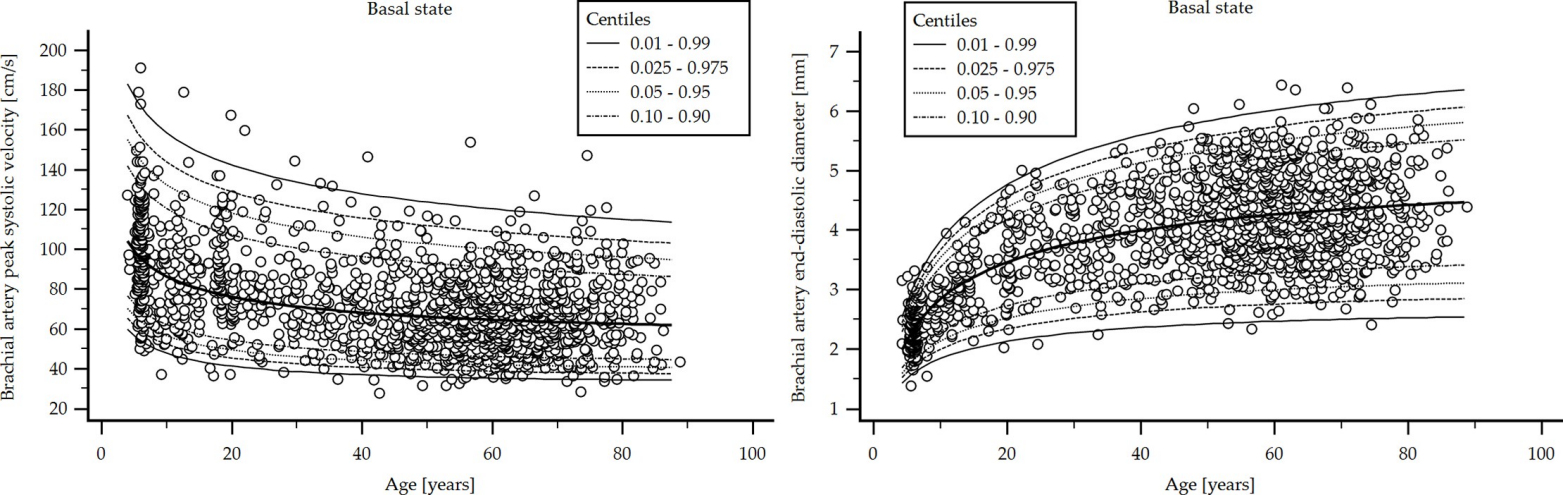
Age-related profiles: Brachial artery diameter and blood flow velocity. Age-related 1th, 2.5th, 5th, 10th, 50th, 90th, 95th, 97.5th and 99th percentiles for brachial artery (BA) end-diastolic diameter (DD) (Top) and peak systolic velocity (PSV) (Bottom).

A step-wise analysis was performed. ***First***, it was analyzed (checked) whether the studied variables (i.e., BA diameter and blood flow velocities) showed (in our population) the expected tendency in terms of age-related variations [Fig 2].

***Second***, VR indexes were calculated, for the entire population and for subgroups defined to determine the RIs. Two different approaches were used to define the RIs subgroups, which enabled comparative analyses with data from other groups: (i) European Reference Values for Arterial Measurements Collaboration Group (called, ´European criteria´) [41–44] and (ii) HUNT3 Fitness Study Group (called, ´HUNT-FIT criteria´) [13]. According to the ´European criteria´ we defined a healthy sub-group that included subjects who did not meet any of the following criteria: (i) history of CVD (i.e., cerebrovascular, coronary, valvular or peripheral arterial disease); (ii) use of BP-, lipid- and/or glucose-lowering drugs; (iii) arterial hypertension (≥18 y: bSBP ≥140 mmHg and/or bDBP ≥90 mmHg; <18 y: bSBP and bDBP <95th percentile for sex, age and BH); (iv) current smoking; (v) diabetes (self-reported and/or fasting plasma glucose ≥126 mg/dl (if available)); (vi) dyslipidemia (self-reported or total cholesterol ≥240 mg/dl or HDL cholesterol <40 mg/dl (if available)); (vii) obesity (≥18 y: BMI ≥30 kg/m^2^; <18 y: z-BMI ≥2.0). In addition, none of them had (i) congenital, chronic or infectious diseases and (ii) cardiac rhythm other than sinus rhythm. The resulting ´European criteria´ RIs sub-group included 1688 subjects (864 females). As stated, a RIs subgroup was also defined in agreement with HUNT3 Fitness Study (a HUNT3 sub-study) criteria. In our knowledge this is among the biggest studies that determined reference data for FMD% in adults [13]. The study included ´Healthy adults´ (n = 4739; age: ≥20 y), defined as subjects without cardio-respiratory diseases or cancer. Pregnancy and/or use of BP-, lipid- and/or glucose-lowering drugs (but not tobacco use, obesity or dyslipemia) were considered exclusion criteria. The resulting HUNT-FIT RIs subgroup included 2609 subjects (1303 females). Tables 2–4 show descriptive data for all the studied subjects and for the RIs subgroups. Complementary data and sex distribution are in (S1-S6 Tables in S1 File).

**Table 2:** Demographic, anthropometric and clinical characteristics.

	All studied subjects				European criteria subgroup				HUNT-FIT criteria subgroup			
Variable	MV	SD	Min	Max	MV	SD	Min	Max	MV	SD	Min	Max
Age [years]	33.9	24.2	2.8	89.0	20.1	16.9	2.8	84.2	23.7	19.3	2.8	85.0
Body weight [Kg.]	61.1	25.3	12.3	150.6	47.9	22.8	12.3	105.0	54.4	24.8	12.3	133.0
Body height [m]	1.55	0.23	0.90	1.97	1.47	0.26	0.90	1.94	1.50	0.25	0.90	1.95
BMI [Kg./m^2^]	24.1	6.0	11.5	71.3	20.4	4.2	11.5	30.0	22.4	5.5	11.5	71.3
z-BMI [SD]	0.94	1.45	-4.63	8.03	0.34	0.92	-4.63	1.98	0.93	1.45	-4.63	8.03
HR [beats/minute]	74	14	35	143	75	15	40	132	75	15	40	132
bSBP [mmHg]	119	17	64	235	112	13	80	171	115	15	80	186
bDBP [mmHg]	69	10	41	129	65	8	47	97	66	9	45	110
Total cholesterol [mg/dl]	200	44	94	379	195	26	99	238	203	41	99	363
HDL cholesterol [mg/dl]	51	15	17	122	58	12	41	100	52	15	17	105
LDL cholesterol [mg/dl]	123	40	28	323	118	26	31	180	127	37	31	293
Triglycerides [mg/dl]	133	86	24	783	93	39	24	272	123	86	24	783
Glycaemia [mg/dl]	94	19	40	307	88	9	40	121	89	10	40	141
TC ≥240 mg/dl [%]	7.2				0.0				5.0			
HDL <40 mg/dl [%]	8.9				0.0				4.9			
Glycaemia ≥126 mg/dl [%]	0.9				0.0				0.2			
Current Smoke [%]	11.4				0.0				10.2			
Hypertension [%]	26.4				0.0				8.2			
Diabetes [%]	5.7				0.0				0.0			
Obesity [%	21.9				0.0				17.9			
Family History CVD [%]	13.5				7.6				9.6			
Physically inactive [%]	45.6				32.3				41.0			
History of CVD [%]	8.8				0.0				0.0			
BP-lowering drugs [%]	21.7				0.0				0.0			
Lipid-lowering drugs [%]	13.5				0.0				0.0			
Glucose-lowering drugs [%]	4.1				0.0				0.0			
Atheroma plaques (%)	22.2				6.6				10.2			

MV: Mean value. SD: Standard deviation. Min., Max.: Minimum and maximum values. z: z-score. BMI: Body mass index. TC: Total cholesterol. CVD: Cardiovascular disease.

**Table 3:** Brachial artery parameters during vascular reactivity test.

	All studied subjects				European criteria subgroup				HUNT-FIT criteria subgroup			
Variable	MV	SD	Min	Max	MV	SD	Min	Max	MV	SD	Min	Max
**Basal**												
bSBP [mmHg]	122	17	78	214	112	14	78	176	116	15	78	184
bDBP [mmHg]	71	10	41	128	66	10	41	95	68	10	41	106
SystD [mm]	4.00	0.96	1.49	6.56	3.44	0.95	1.49	6.50	3.64	0.96	1.49	6.50
DD [mm]	3.82	0.96	1.39	6.44	3.26	0.95	1.39	6.39	3.46	0.96	1.39	6.39
PD [mm]	0.18	0.09	0.02	0.86	0.18	0.08	0.04	0.67	0.18	0.09	0.02	0.86
PSV [cm/s]	74.31	22.75	27.80	191.10	81.13	24.58	27.80	191.10	79.17	24.20	27.80	191.10
EDV [cm/s]	3.43	8.87	-48.1	52.60	3.38	10.56	-48.1	33.00	3.84	10.21	-48.1	52.60
RI	0.95	0.09	0.56	1.41	0.95	0.10	0.56	1.41	0.95	0.10	0.56	1.41
PS WSS [dyne/cm^2^]	56.53	30.04	16.40	260.58	72.73	37.52	17.81	260.58	66.66	34.55	17.81	260.58
ED WSS [dyne/cm^2^]	3.40	7.19	-27.1	56.65	4.83	9.05	-27.1	41.81	4.50	8.58	-27.1	56.65
**Pre-release**												
SystD [mm]	4.32	0.83	2.20	6.79	3.98	0.86	2.20	6.56	4.09	0.83	2.20	6.56
DD [mm]	4.16	0.83	1.96	6.70	3.81	0.85	1.96	6.40	3.93	0.83	1.96	6.40
PD [mm]	0.16	0.10	0.00	1.28	0.17	0.11	0.00	1.28	0.17	0.11	0.00	1.28
PSV [cm/s]	59.69	17.07	19.40	130.70	62.38	17.47	19.40	121.40	62.39	17.68	19.40	124.00
EDV [cm/s]	-1.16	5.44	-45.2	66.00	-1.71	7.67	-45.2	66.00	-1.49	6.86	-45.2	66.00
RI	1.02	0.07	0.26	1.51	1.02	0.09	0.26	1.50	1.02	0.09	0.26	1.51
PS WSS [dyne/cm^2^]	40.53	16.11	12.74	111.19	46.17	18.27	13.87	103.38	44.92	17.68	13.87	111.19
ED WSS [dyne/cm^2^]	-0.78	4.10	-37.4	51.19	-1.26	6.23	-37.4	51.19	-1.12	5.41	-37.4	51.19
**Hyperemia**												
SystD [mm]	4.41	0.82	2.18	6.85	4.06	0.85	2.18	6.51	4.18	0.82	2.18	6.51
DD [mm]	4.27	0.81	2.02	6.73	3.92	0.85	2.02	6.42	4.03	0.82	2.02	6.42
PD [mm]	0.14	0.09	0.01	1.26	0.14	0.07	0.01	0.55	0.14	0.09	0.01	1.26
PSV [cm/s]	100.27	24.82	43.60	257.00	101.86	24.97	48.00	180.00	101.53	25.16	48.00	200.00
EDV [cm/s]	28.09	12.24	0.00	96.00	29.32	11.61	10.00	80.00	29.40	12.14	0.00	85.00
RI	0.72	0.09	0.42	1.01	0.71	0.08	0.43	0.92	0.71	0.09	0.43	1.00
PS WSS [dyne/cm^2^]	65.37	21.77	21.57	170.85	71.54	25.08	26.09	170.85	69.88	24.01	26.09	170.85
ED WSS [dyne/cm^2^]	18.97	9.24	0.00	72.49	21.43	9.95	5.52	58.98	21.00	9.72	0.00	58.98

MV: Mean value. SD: Standard deviation. Min., Max.: Minimum and maximum values. bSBP, bDBP: Brachial systolic and diastolic Blood pressure. SystD, DD and PD: Systolic, diastolic and pulsate diameter. PSV, EDV: Peak and end-diastolic blood flow velocity. RI: Resistive index. PS WSS and ED WSS: Peak systolic and end-diastolic wall shear stress.

**Table 4:** Vascular reactivity indexes: Levels.

	All studied subjects				European criteria subgroup				HUNT-FIT criteria subgroup			
Variable	MV	SD	Min	Max	MV	SD	Min	Max	MV	SD	Min	Max
**1. Macrovascular and Macro/Microvascular Reactivity Indexes**												
**1.1. Basal vs. Hyperemia indexes (not adjusted for stimulus)**												
ΔDD_Peak_Bas_ [mm]	0.20	0.18	-1.38	1.65	0.23	0.16	-0.46	1.09	0.22	0.17	-0.46	1.15
DD Ratio _Peak_Bas_ [%]	105.33	5.10	76.45	151.72	106.72	4.89	91.10	135.24	106.09	5.25	90.99	143.11
FMD% [%]	5.33	5.10	-23.55	51.72	6.72	4.89	-8.90	35.24	6.09	5.25	-9.01	43.11
TPD_FMD% [s]	90.32	44.85	0.08	224.64	90.42	44.63	0.08	224.64	89.65	45.81	0.08	224.64
**1.2. Basal vs. Hyperemia indexes (adjusted for stimulus)**												
ΔDD_Peak_Bas_/ΔV_Peak_Bas_ [mm/cm/s]	0.009	0.013	-0.152	0.158	0.010	0.009	-0.013	0.086	0.009	0.012	-0.152	0.086
FMD/ΔV_Peak_Bas_ [1/cm/s]	0.002	0.010	-0.188	0.226	0.003	0.002	-0.003	0.021	0.003	0.014	-0.188	0.226
pFMDv	0.02	0.24	-4.15	2.24	0.03	0.06	-0.01	0.40	0.01	0.33	-4.15	2.24
FMD%WSS	0.03	0.18	-1.85	1.67	0.04	0.09	-0.01	0.74	0.02	0.20	-1.85	1.31
**1.3. Basal vs. Pre-release indexes (not adjusted for stimulus)**												
ΔD_Pre_Basal_ [mm]	0.10	0.20	-1.06	1.43	0.12	0.17	-0.56	1.13	0.11	0.19	-0.69	1.13
DD Ratio _Pre_Basal_ [%]	102.66	5.47	78.23	144.83	103.67	5.07	89.17	131.39	103.14	5.63	83.09	140.89
L-FMC% [%]	2.66	5.47	-21.77	44.83	3.67	5.07	-10.83	31.39	3.14	5.63	-16.91	40.89
**1.4. Basal vs. Pre-release indexes (adjusted for stimulus)**												
ΔDD_Pre_Basal_/ΔEDV_Pre_Basal_	0.00	0.07	-0.39	0.86	0.01	0.11	-0.22	0.86	0.00	0.09	-0.33	0.86
L-FMC/ΔV_Pre_Basal_ [1/cm/s]	0.000	0.026	-0.092	0.380	0.005	0.045	-0.061	0.380	0.001	0.034	-0.092	0.380
pL-FMCv	-0.01	0.11	-0.69	1.45	0.00	0.09	-0.19	0.52	0.00	0.13	-0.41	1.45
L-FMC%/WSS	-4.67	27.33	-327.07	125.94	-3.20	27.62	-105.85	125.94	-4.96	32.42	-327.07	125.94
**1.5.Total Vascular Reactivity**												
TVR [%]	2.71	4.52	-33.45	27.93	3.12	3.81	-15.45	19.31	3.00	4.37	-21.62	22.09
**1.6. Hyperemic shear-stress**												
ΔWSS_Peak_Basal_ [dyn/cm^2^]	16.66	8.86	-9.72	72.49	19.24	9.63	3.19	58.98	18.38	9.50	-9.72	58.98
**2. Microvascular Reactivity Indexes**												
ΔV_Peak_Basal_ [cm/s]	24.78	11.54	-10.00	96.00	26.33	11.01	5.87	70.62	25.81	11.62	-10.00	70.62
ΔRI_Peak_Basal_	-0.24	0.10	-0.59	0.15	-0.25	0.09	-0.55	-0.04	-0.24	0.10	-0.59	0.15
ΔRI%_Peak_Basal_ [%]	-24.25	9.89	-54.13	23.44	-25.42	8.77	-51.51	-5.06	-25.06	9.70	-54.13	17.65

MV: Mean value. SD: Standard deviation. Min., Max.: Minimum and maximum values. For vascular reactivity indexes abbreviations: See text and Table 1. Peak: Peak value during reactive hyperemic conditions. Bas: Basal or baseline conditions. Pre: Pre-release conditions.

***Third***, we analyzed the association of carotid and/or femoral atherosclerotic plaques presence with VR indexes by means of point-biserial correlations without and with Bootstrapping (sample number = 1000; Bias Corrected accelerated Confidence Intervals; simple sampling). Results showed that for healthy asymptomatic subjects included in the RIs subgroups, atherosclerotic plaques presence was not associated with VR indexes. Then, subjects with atherosclerotic plaques were not excluded from the RIs subgroups. This way, our inclusion and exclusion criteria completely agreed with those of the HUNT-FIT and European Group.

***Fourth***, we assessed the association (simple bivariate correlations) between VR indexes [Table 5]. This enabled to identify that alternative indexes used to assess ´macro´, ´macro/micro´ and ´micro´ VR were not equivalent (but could give different and complimentary data). Therefore, it was necessary to define RIs (separately) for each VR index.

**Table 5:** Association between vascular reactivity indexes.

		ΔDD_Peak_Bas_[mm]	DDRatio_Peak_Bas_[%]	FMD% [%]	TPD_FMD% [s]	ΔDD _Peak-Bas_/ΔV_Peak-Bas_ [mm/cm/s]	FMD/ΔV_Peak_Bas_ [1/cm/s]	pFMDv	FMD%WSS	ΔD_Pre_Bas_[mm]	DD Ratio _Pre_Bas_[%]	L-FMC% [%]
**Basal vs. Hyperemia indexes (not adjusted for stimulus)**												
ΔDD _Peak_Bas_ [mm]	R	1.00	0.94	0.94	0.00	0.60	0.59	0.44	0.47	0.44	0.49	0.49
	P	------	**<0.001**	**<0.001**	0.997	**<0.001**	**<0.001**	**<0.001**	**<0.001**	**<0.001**	**<0.001**	**<0.001**
DD Ratio _Peak_Bas_ [%]	R	0.943	1.000	1.000	-0.001	0.596	0.657	0.505	0.579	0.473	0.574	0.574
	P	**<0.001**	------	**<0.001**	0.986	**<0.001**	**<0.001**	**<0.001**	**<0.001**	**<0.001**	**<0.001**	**<0.001**
FMD% [%]	R	0.943	1.000	1.000	-0.001	0.596	0.657	0.505	0.579	0.473	0.574	0.574
	P	**<0.001**	**<0.001**	------	0.986	**<0.001**	**<0.001**	**<0.001**	**<0.001**	**<0.001**	**<0.001**	**<0.001**
TPD_FMD% [s]	R	0.000	-0.001	-0.001	1.000	-0.042	-0.033	-0.023	0.000	-0.024	-0.014	-0.014
	P	0.997	0.986	0.986	------	0.412	0.512	0.653	0.994	0.638	0.784	0.784
**Basal vs. Hyperemia indexes (adjusted for stimulus)**												
ΔDD_Peak-Bas_/ΔV_Peak-Bas [_mm/cm/s]	R	0.596	0.596	0.596	-0.042	1.000	0.969	0.917	0.791	0.353	0.387	0.387
	P	**<0.001**	**<0.001**	**<0.001**	0.412	------	**<0.001**	**<0.001**	**<0.001**	**<0.001**	**<0.001**	**<0.001**
FMD/ΔV _Peak_Bas_ [1/cm/s]	R	0.586	0.657	0.657	-0.033	0.969	1.000	0.930	0.866	0.375	0.452	0.452
	P	**<0.001**	**<0.001**	**<0.001**	0.512	**<0.001**	------	**<0.001**	**<0.001**	**<0.001**	**<0.001**	**<0.001**
pFMDv	R	0.442	0.505	0.505	-0.023	0.917	0.930	1.000	0.871	0.282	0.339	0.339
	P	**<0.001**	**<0.001**	**<0.001**	0.653	**<0.001**	**<0.001**	------	**<0.001**	**<0.001**	**<0.001**	**<0.001**
FMD%WSS	R	0.470	0.579	0.579	0.000	0.791	0.866	0.871	1.000	0.349	0.444	0.444
	P	**<0.001**	**<0.001**	**<0.001**	0.994	**<0.001**	**<0.001**	**<0.001**	------	**<0.001**	**<0.001**	**<0.001**
**Basal vs. Pre-release indexes (not adjusted for stimulus)**												
ΔD_Pre_Bas_[mm]	R	0.438	0.473	0.473	-0.024	0.353	0.375	0.282	0.349	1.000	0.964	0.964
	P	**<0.001**	**<0.001**	**<0.001**	0.638	**<0.001**	**<0.001**	**<0.001**	**<0.001**	------	**<0.001**	**<0.001**
DD Ratio _Pre_Bas_[%]	R	0.485	0.574	0.574	-0.014	0.387	0.452	0.339	0.444	0.964	1.000	1.000
	P	**<0.001**	**<0.001**	**<0.001**	0.784	**<0.001**	**<0.001**	**<0.001**	**<0.001**	**<0.001**	------	**<0.001**
L-FMC% [%]	R	0.485	0.574	0.574	-0.014	0.387	0.452	0.339	0.444	0.964	1.000	1.000
	P	**<0.001**	**<0.001**	**<0.001**	0.784	**<0.001**	**<0.001**	**<0.001**	**<0.001**	**<0.001**	**<0.001**	------
**Basal vs. Pre-release indexes (adjusted for stimulus)**												
ΔDD_Pre_Bas_/ΔEDV_Pre_Bas_	R	-0.116	-0.144	-0.144	0.003	-0.098	-0.110	-0.098	-0.109	-0.372	-0.373	-0.373
	P	**0.023**	**0.005**	**0.005**	0.960	0.056	**<0.001**	0.054	**<0.001**	**<0.001**	**<0.001**	**<0.001**
L-FMC/ΔV_Pre_Bas_ [1/cm/s]	R	-0.157	-0.202	-0.202	0.006	-0.122	-0.147	-0.120	-0.145	-0.403	-0.428	-0.428
	P	**0.002**	**<0.001**	**<0.001**	0.901	**0.017**	**0.004**	**0.018**	**0.004**	**<0.001**	**<0.001**	**<0.001**
pL-FMCv	R	-0.294	-0.333	-0.333	0.006	-0.248	-0.278	-0.208	-0.269	-0.485	-0.496	-0.496
	P	**<0.001**	**<0.001**	**<0.001**	0.904	**<0.001**	**<0.001**	**<0.001**	**<0.001**	**<0.001**	**<0.001**	**<0.001**
L-FMC%/WSS	R	-0.242	-0.274	-0.274	-0.018	-0.211	-0.235	-0.195	-0.237	-0.363	-0.372	-0.372
	P	**<0.001**	**<0.001**	**<0.001**	0.727	**<0.001**	**<0.001**	**<0.001**	**<0.001**	**<0.001**	**<0.001**	**<0.001**
**Total Vascular Reactivity**												
TVR [%]	R	0.527	0.497	0.497	0.014	0.248	0.247	0.198	0.168	-0.500	-0.426	-0.426
	P	**<0.001**	**<0.001**	**<0.001**	0.786	**<0.001**	**<0.001**	**<0.001**	**0.001**	**<0.001**	**<0.001**	**<0.001**
**Hyperemic shear-stress**												
ΔWSS _Peak_Bas_ [dyn/cm^2^]	R	-0.059	-0.009	-0.009	-0.106	-0.200	-0.174	-0.135	-0.191	-0.087	-0.074	-0.074
	P	0.247	0.854	0.854	**0.037**	**<0.001**	**0.001**	**0.008**	**<0.001**	0.088	0.146	0.146
**Microvascular Indexes**												
ΔV_Peak_Bas_ [cm/s]	R	0.005	0.000	0.000	-0.100	-0.174	-0.172	-0.124	-0.180	-0.034	-0.039	-0.039
	P	0.920	0.993	0.993	0.051	**0.001**	**0.001**	**0.015**	**<0.001**	0.510	0.444	0.444
ΔRI_Peak_Bas_	R	0.089	0.098	0.098	0.123	0.180	0.180	0.206	0.237	0.059	0.065	0.065
	P	0.083	0.054	0.054	**0.015**	**<0.001**	**<0.001**	**<0.001**	**<0.001**	0.247	0.204	0.204
ΔRI%_Peak_Bas_ [%]	R	0.072	0.081	0.081	0.130	0.164	0.164	0.163	0.221	0.061	0.069	0.069
	P	0.160	0.111	0.111	**0.011**	**0.001**	**0.001**	**0.001**	**<0.001**	0.229	0.177	0.177
			**ΔDD**_**Pre_Bas**_**/ΔEDV**_**Pre_Bas**_	**L-FMC/ΔV**_**Pre_Bas**_ **[1/cm/s]**	**pL-FMCv**	**L-FMC%/WSS**	**TVR [%]**	**ΔV**_**Pek_Bas**_ **[cm/s]**	**ΔWSS**_**Peak_Bas**_**[dyn/cm**^**2**^**]**	**ΔRI**_**Peak_Bas**_	**ΔRI%**_**Peak_Bas**_**[%]**	
**Basal vs. Hyperemia indexes (not adjusted for stimulus)**												
ΔDD _Peak_Bas_ [mm]	R		-0.12	-0.16	-0.29	-0.24	0.53	0.01	-0.06	0.09	0.07	
	p		**0.023**	**0.002**	**<0.001**	**<0.001**	**<0.001**	0.920	0.247	0.083	0.160	
DD Ratio _Peak_Bas_ [%]	R		-0.144	-0.202	-0.333	-0.274	0.497	0.000	-0.009	0.098	0.081	
	p		**0.005**	**<0.001**	**<0.001**	**<0.001**	**<0.001**	0.993	0.854	0.054	0.111	
FMD% [%]	R		-0.144	-0.202	-0.333	-0.274	0.497	0.000	-0.009	0.098	0.081	
	p		**0.005**	**<0.001**	**<0.001**	**<0.001**	**<0.001**	0.993	0.854	0.054	0.111	
TPD_FMD% [s]	R		0.003	0.006	0.006	-0.018	0.014	-0.100	-0.106	0.123	0.130	
	p		0.960	0.901	0.904	0.727	0.786	0.051	**0.037**	**0.015**	**0.011**	
**Basal vs. Hyperemia indexes (adjusted for stimulus)**												
ΔDD_Peak_Bas_/ΔV_Peak_Bas_ [mm/cm/s]	R		-0.098	-0.122	-0.248	-0.211	0.248	-0.174	-0.200	0.180	0.164	
	p		0.056	**0.017**	**<0.001**	**<0.001**	**<0.001**	**0.001**	**<0.001**	**<0.001**	**0.001**	
FMD/ΔV _Peak_Bas_ [1/cm/s]	R		-0.110	-0.147	-0.278	-0.235	0.247	-0.172	-0.174	0.180	0.164	
	p		**0.031**	**0.004**	**<0.001**	**<0.001**	**<0.001**	**0.001**	**0.001**	**<0.001**	**0.001**	
pFMDv	R		-0.098	-0.120	-0.208	-0.195	0.198	-0.124	-0.135	0.206	0.163	
	p		0.054	**0.018**	**<0.001**	**<0.001**	**<0.001**	**0.015**	**0.008**	**<0.001**	**0.001**	
FMD%WSS	R		-0.109	-0.145	-0.269	-0.237	0.168	-0.180	-0.191	0.237	0.221	
	p		**0.033**	**0.004**	**<0.001**	**<0.001**	**0.001**	**<0.001**	**<0.001**	**<0.001**	**<0.001**	
**Basal vs. Pre-release indexes (not adjusted for stimulus)**												
ΔD _Pre_Bas_ [mm]	R		-0.372	-0.403	-0.485	-0.363	-0.500	-0.034	-0.087	0.059	0.061	
	p		**<0.001**	**<0.001**	**<0.001**	**<0.001**	**<0.001**	0.510	0.088	0.247	0.229	
DD Ratio _Pre_Bas_ [%]	R		-0.373	-0.428	-0.496	-0.372	-0.426	-0.039	-0.074	0.065	0.069	
	p		**<0.001**	**<0.001**	**<0.001**	**<0.001**	**<0.001**	0.444	0.146	0.204	0.177	
L-FMC% [%]	R		-0.373	-0.428	-0.496	-0.372	-0.426	-0.039	-0.074	0.065	0.069	
	p		**<0.001**	**<0.001**	**<0.001**	**<0.001**	**<0.001**	0.444	0.146	0.204	0.177	
**Basal vs. Pre-release indexes (adjusted for stimulus)**												
ΔDD _Pre_Bas_/ΔEDV_Pre_Bas_	R		1.000	0.983	-0.461	-0.406	0.236	0.001	0.021	-0.020	-0.010	
	p		------	**<0.001**	**<0.001**	**<0.001**	**<0.001**	0.980	0.681	0.693	0.848	
L-FMC/ΔV _Pre_Bas_ [1/cm/s]	R		0.983	1.000	-0.416	-0.405	0.231	0.008	0.033	-0.017	-0.011	
	p		**<0.001**	------	**<0.001**	**<0.001**	**<0.001**	0.879	0.523	0.737	0.836	
pL-FMCv	R		-0.461	-0.416	1.000	0.921	0.158	0.088	0.124	-0.137	-0.115	
	p		**<0.001**	**<0.001**	------	**<0.001**	**0.002**	0.083	**0.015**	**0.007**	**0.023**	
L-FMC%/WSS	R		-0.406	-0.405	0.921	1.000	0.092	0.086	0.124	-0.186	-0.138	
	p		**<0.001**	**<0.001**	**<0.001**	------	0.072	0.091	**0.015**	**<0.001**	**0.007**	
**Total Vascular Reactivity**												
TVR [%]	R		0.236	0.231	0.158	0.092	1.000	0.042	0.068	0.040	0.017	
	p		**<0.001**	**<0.001**	**0.002**	0.072	------	0.412	0.181	0.439	0.742	
**Hyperemic shear-stress**												
ΔWSS _Peak_Bas_ [dyn/cm^2^]	R		0.021	0.033	0.124	0.124	0.068	0.930	1.000	-0.716	-0.745	
	p		0.681	0.523	**0.015**	**0.015**	0.181	**<0.001**	------	**<0.001**	**<0.001**	
ΔWSS% _Peak_Bas_ [%]	R		0.017	0.050	0.010	-0.034	0.039	0.098	0.095	-0.020	-0.078	
	p		0.744	0.332	0.838	0.510	0.449	0.056	0.063	0.700	0.125	
**Microvascular Indexes**												
ΔV_Peak_Bas_ [cm/s]	R		0.001	0.008	0.088	0.086	0.042	1.000	0.930	-0.753	-0.803	
	p		0.980	0.879	0.083	0.091	0.412	------	**<0.001**	**<0.001**	**<0.001**	
ΔV% _Peak_Bas_ [%]	R		0.010	0.041	0.006	-0.038	0.047	0.098	0.093	-0.020	-0.078	
	p		0.850	0.427	0.908	0.460	0.354	0.055	0.067	0.699	0.124	
ΔRI _Peak_Bas_	R		-0.020	-0.017	-0.137	-0.186	0.040	-0.753	-0.716	1.000	0.966	
	p		0.693	0.737	**0.007**	**<0.001**	0.439	**<0.001**	**<0.001**	------	**<0.001**	
ΔRI% _Peak_Bas_ [%]	R		-0.010	-0.011	-0.115	-0.138	0.017	-0.803	-0.745	0.966	1.000	
	p		0.848	0.836	**0.023**	**0.007**	0.742	**<0.001**	**<0.001**	**<0.001**	------	

***Fifth***, we evaluated whether age and/or sex-specific RIs were necessary using multiple linear regression models that included interaction analysis (Sex*Age) with *Johnson-Neyman significance regions* definition [S7 Table in S1 File]. Variables "y", "x" and "w" (moderating variable) were assigned, respectively, to the VR index (e.g., FMD%), sex and age. Thereafter we identified indexes that: (i) required sex-specific RIs only from a certain age, (ii) required sex-specific RIs regardless of age, (iii) did not require sex-specific RIs or (iv) did not require age- and sex-specific RIs [S7 and S8 Tables in S1 File]. The association between BA diameter, bSBP and bDBP and VR indexes was evaluated (simple bivariate (zero-order) and partial correlations (adjusting for age and sex)).

Finally, as a ***sixth step***, age-related RIs were obtained for the two different RIs subgroups. Age-related equations for mean value (MV) and standard deviation (SD) were obtained (for all, females and males) [S9 Table in S1 File]. To this end, parametric regression methods based on fractional polynomials (FPs) were applied. These were described by Royston and Wright [45], considered in the European Reference Values for Arterial Measurements Collaboration Group methodological strategy, and already used by our group to define RIs for hemodynamic, ventricular, atrial and arterial parameters [20,25,46–48]. Briefly, fitting FPs age-specific MV and SD regression curves for the different variables were defined using an iterative procedure (generalized least squares). Then, age-specific equations were obtained. For instance, FMD% mean equation would be: FMD% mean = a+b*Age^p^+c*Age^q^+…, where a, b, c, … are the coefficients, and p, q, … are powers, with numbers selected from the set [-2, -1, -0.5, 0, 0.5, 1, 2, 3] estimated from the regression for the mean FMD% curve, and likewise from the SD curve. Continuing the example, FPs with powers [1,2], that is, with p = 1 and q = 2, illustrate an equation with the form a+b*age+c*age^2^ [45]. Residuals were used to assess the model fit, which was deemed appropriate if the scores were normally distributed, with a mean of 0 and a SD of 1, randomly scattered above and below 0 when plotted against age. Best fitted curves considering visual and mathematical criteria (Kurtosis and Skewness levels) were selected.

From the equations obtained for MV and SD [S9 Table in S1 File], age-specific percentiles were defined using standard normal distribution (Z). The 1th, 2.5th, 5th, 10th, 25th, 50th, 75th, 90th, 95th, 97.5th and 99th percentiles were calculated, for example for FMD% as: mean FMD%+Zp*SD, where Zp assumed the values -2.3263, -1.9599, -1.6448, -1.2815, -0.6755, 0, 0.6755, 1.2815, 1.6448, 1.9599 and 2.3263, respectively. Following the described approach, RIs were defined for each macro, macro/micro and micro VR index listed in Table 1. Data for each year of life (in the range considered) are in (S10-S35 Tables in S1 File for ´European Criteria´ RIs subgroup; S36-S63 Tables in S1 File for ´HUNT-FIT criteria´ RIs subgroup). S2 File shows age-related profiles (percentile curves) for the different VR indexes.

The minimum sample size required for RIs construction was 377 [49]. Like in previous works and according to central limit theorem, normal distribution was considered (taking into account Kurtosis and Skewness coefficients distribution and sample size ˃30) [50]. Data analysis was done using MedCalc Statistical Software (version 18.5, MedCalc Inc., Ostend, Belgium) and IBM-SPSS Software (version 26, SPSS Inc., Illinois, USA). PROCESS version 3.5 (SPSS extension) was used for moderation (interaction) analysis [51]. A p<0.05 was considered statistically significant. Evans’s Empirical Classifications of Interpreting Correlation Strength by Using r was applied: r<0.20, very weak; r: 0.20–0.39, weak; r: 0.40–0.59, moderate; r: 0.60–0.79, strong; r ≥0.80, very strong [52].

## Results

### Subjects characteristics

Table 2 shows demographic, clinical, CV and anthropometric data. Note the wide age-ranges considered. Table 3 shows BA parameters during VR test. VR indexes are shown in Table 4.

### Association between vascular reactivity indexes

**Fig 3:**
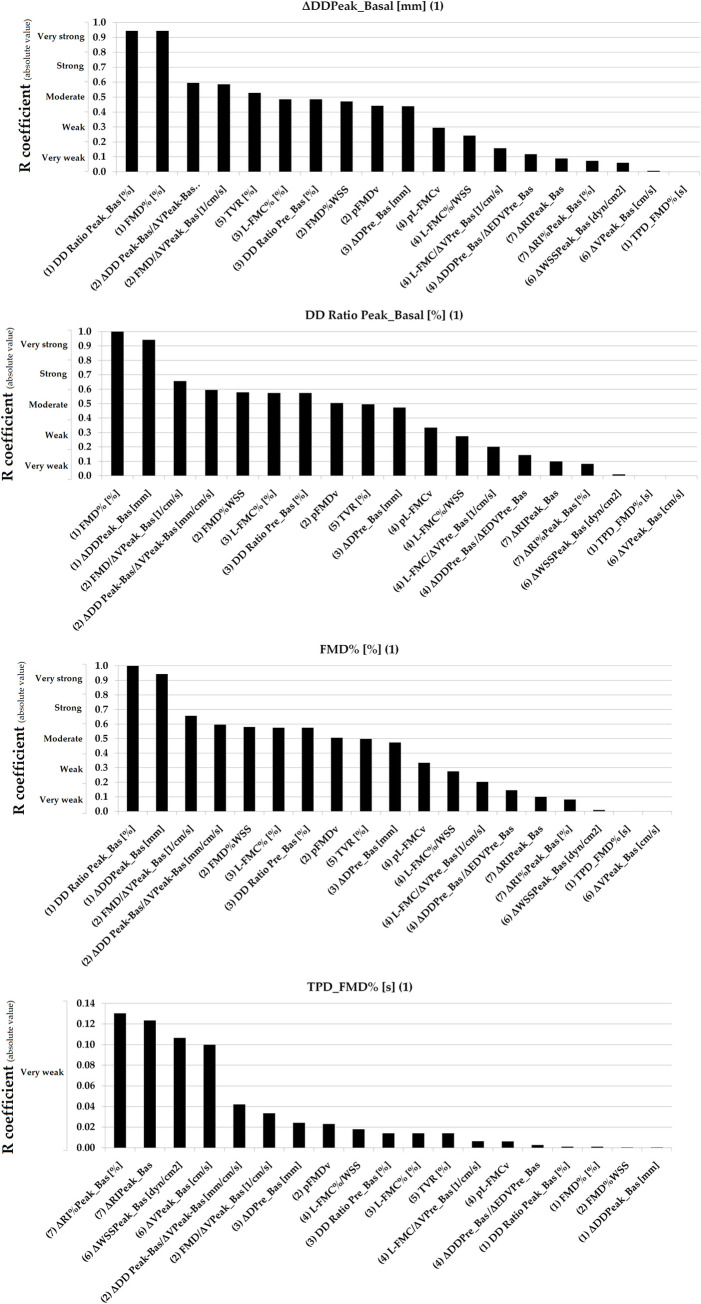
Absolute values for R coefficients (ordered from highest to lowest). Numbers in parentheses: (1 and 2): Basal vs. RH Indexes (non-adjusted and adjusted for stimulus); (3 and 4): Basal vs. Pre-release Indexes (non-adjusted and adjusted for stimulus); (5): Total Vascular Reactivity; (6): Hyperemic Stimulus Indexes; (7): Microvascular Indexes. Evans’ Empirical Classifications of Interpreting Correlation Strength by Using ‘r’ was indicated (´y´ axis). RH: Reactive hyperemia.

**Fig 4:**
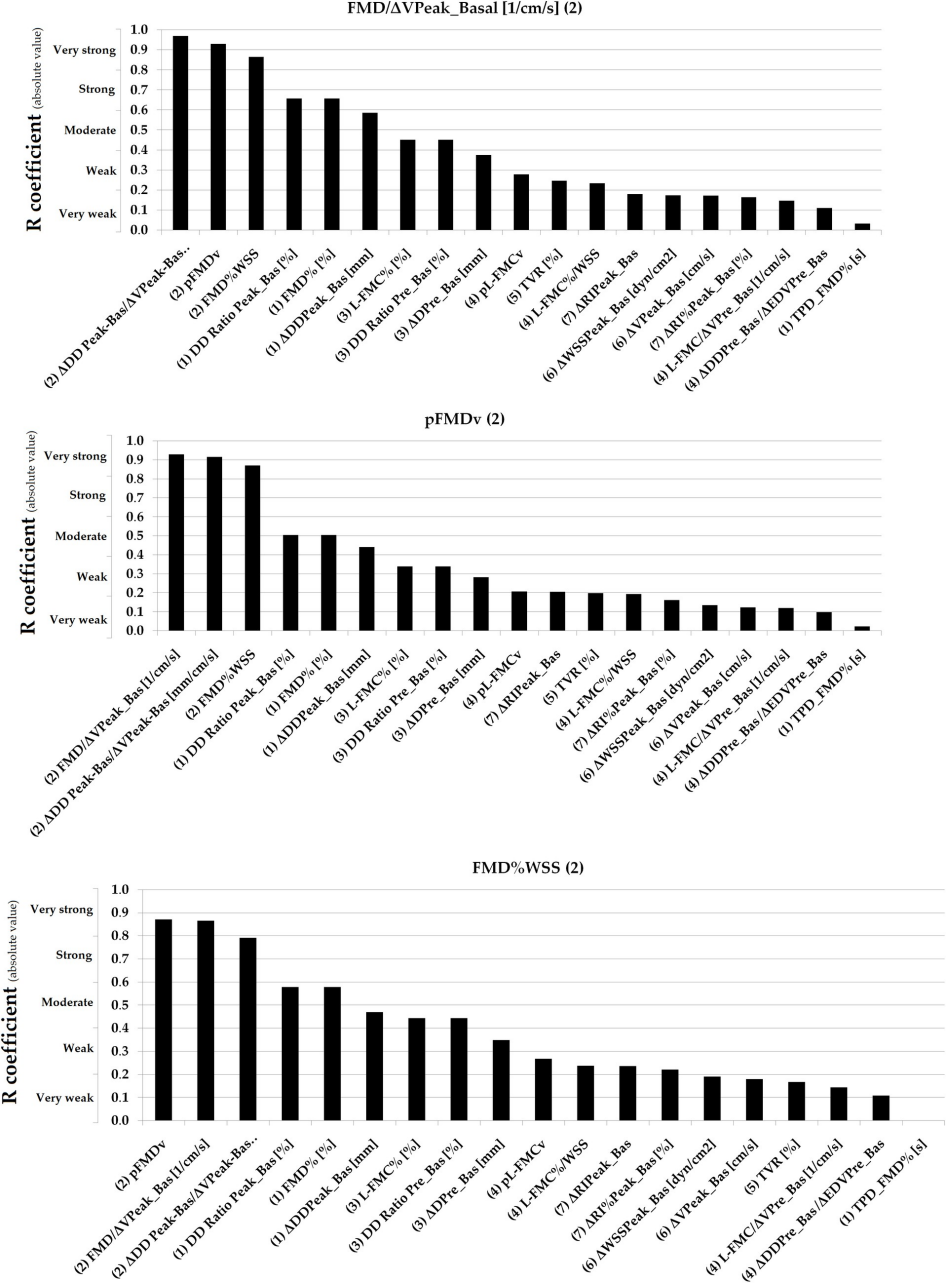
Absolute values for R coefficients (ordered from highest to lowest). Numbers in parentheses: (1 and 2): Basal vs. RH Indexes (non-adjusted and adjusted for stimulus); (3 and 4): Basal vs. Pre-release Indexes (non-adjusted and adjusted for stimulus); (5): Total Vascular Reactivity; (6): Hyperemic Stimulus Indexes; (7): Microvascular Indexes. Evans’ Empirical Classifications of Interpreting Correlation Strength by Using ‘r’ was indicated (´y´ axis). RH: Reactive hyperemia.

**Fig 5:**
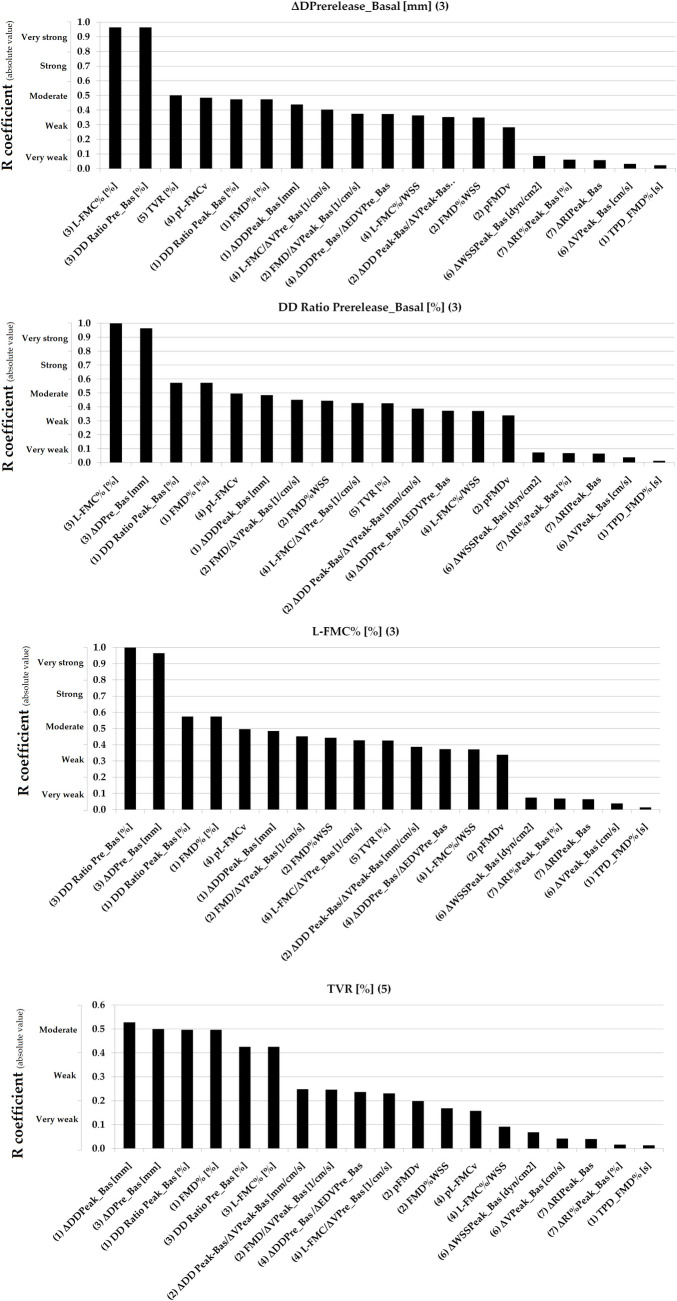
Absolute values for R coefficients (ordered from highest to lowest). Numbers in parentheses: (1 and 2): Basal vs. RH Indexes (non-adjusted and adjusted for stimulus); (3 and 4): Basal vs. Pre-release Indexes (non-adjusted and adjusted for stimulus); (5): Total Vascular Reactivity; (6): Hyperemic Stimulus Indexes; (7): Micro-vascular Indexes. Evans’ Empirical Classifications of Interpreting Correlation Strength by Using ‘r’ was indicated (´y´ axis). RH: Reactive hyperemia.

**Fig 6:**
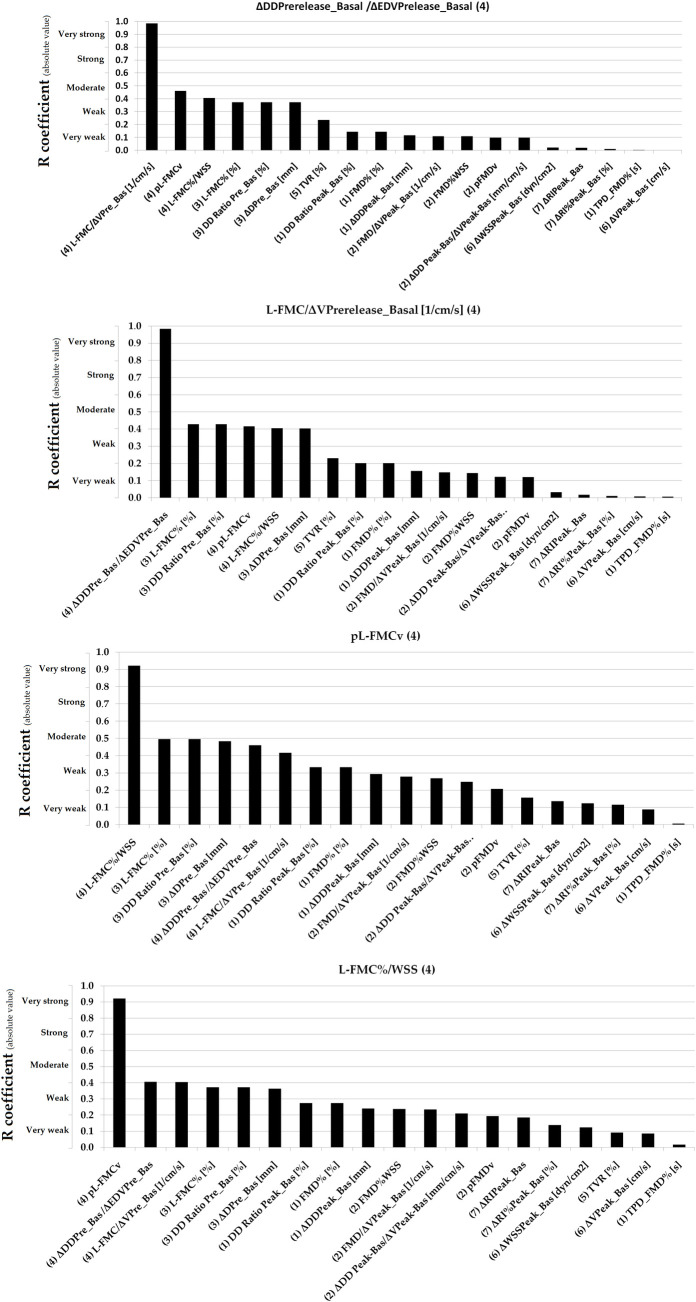
Absolute values for R coefficients (ordered from highest to lowest). Numbers in parentheses: (1 and 2): Basal vs. RH Indexes (non-adjusted and adjusted for stimulus); (3 and 4): Basal vs. Pre-release Indexes (non-adjusted and adjusted for stimulus); (5): Total Vascular Reactivity; (6): Hyperemic Stimulus Indexes; (7): Micro-vascular Indexes. Evans’ Empirical Classifications of Interpreting Correlation Strength by Using ‘r’ was indicated (´y´ axis). RH: Reactive hyperemia.

**Fig 7:**
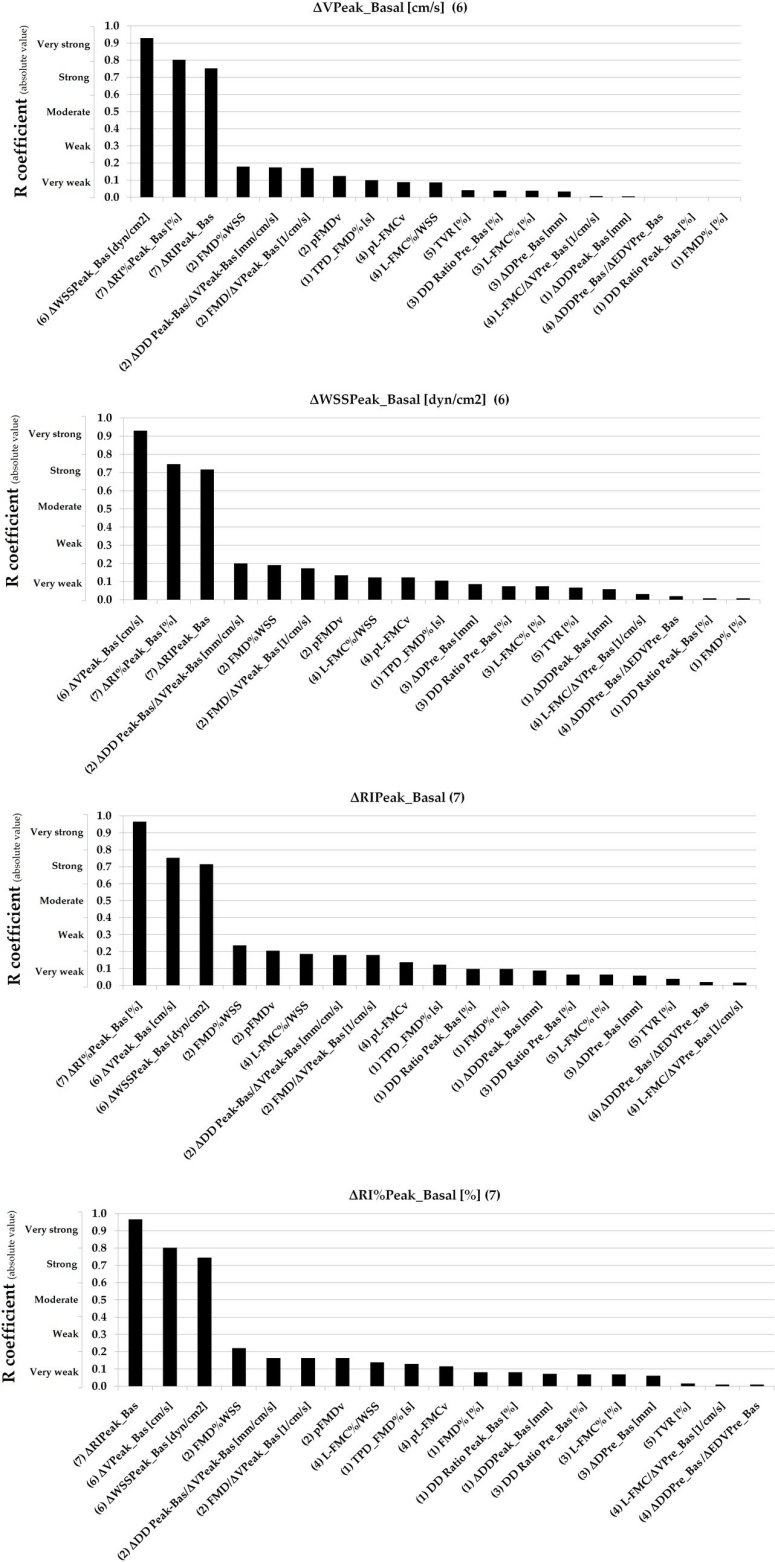
Absolute values for R coefficients (ordered from highest to lowest). Numbers in parentheses: (1 and 2): Basal vs. RH Indexes (non-adjusted and adjusted for stimulus); (3 and 4): Basal vs. Pre-release Indexes (non-adjusted and adjusted for stimulus); (5): Total Vascular Reactivity; (6): Hyperemic Stimulus Indexes; (7): Micro-vascular Indexes. Evans’ Empirical Classifications of Interpreting Correlation Strength by Using ‘r’ was indicated (´y´ axis). RH: Reactive hyperemia.

Simple bivariate correlation data are shown in Table 5. Figs 3–7 show absolute values for r coefficient (ordered from highest to lowest).

In general terms, VR indexes showed no association with each other. Very strong (r ≥0.80) or strong (r: 0.60–0.79) associations were only observed for indexes belonging to the same group (e.g., some macro VR indexes) and thus are mathematically related (e.g., FMD% vs. ΔDD_Peak-Basal_, r˃0.90) [Table 5, Figs 3–7]. Conversely, parameters from different class or groups (e.g. macro and micro VR indexes) showed non-significant, weak or very week associations [Table 5].

L-FMC% and FMD% showed a moderate association [Table 5, Fig 5]. TPD_FMD% showed no association with other VR indexes (r<0.14) [Table 5, Fig 3].

When analyzing recruitable EF and baseline data (i.e., ΔDD Peak-Basal/ΔV Peak-Basal; FMD/ΔV Peak_Basal; pFMDv; FMD%WSS; ΔDD Prerelease_Basal/ΔEDV Prelease_Basal; L-FMC/ΔV Prerelease_Basal; pL-FMCv and L-FMC%/WSS) the adjustment for stimulus did not result in increased associations [Table 5, Figs 3–7].

Macro and macro/micro VR indexes showed either no significant or very weak associations with indexes evaluating the micro VR [Table 5, Fig 7].

### Association between vascular reactivity indexes and brachial artery diameter and blood pressure levels

**Fig 8:**
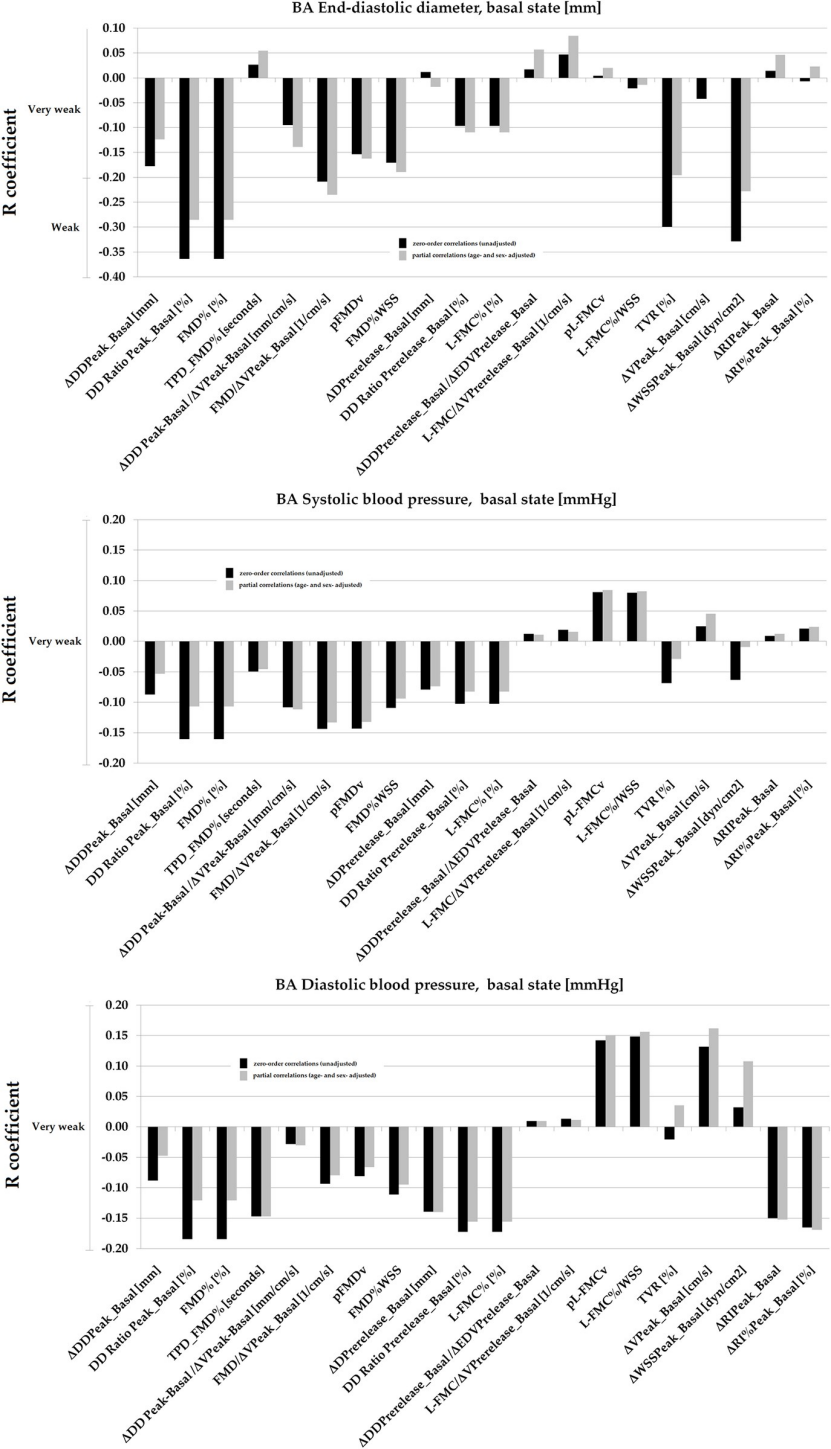
Associations (simple and partial correlations [adjustment for age and sex]) between vascular reactivity indexes and brachial artery (BA) end-diastolic diameter, and systolic and diastolic blood pressure.

Basal BA DD showed weak association with arterial capability to dilate in response to a hyperemic stimulus (data non-adjusted for stimulus). The associations were very weak when data co-adjusted for stimulus were considered. In turn, basal BA DD showed no association with pre-release data (adjusted for stimulus) or micro-vascular resistances changes [Fig 8]. bSBP and bDBP were negatively associated (very weak levels) with some VR indexes [Fig 8]. The levels of the associations were lower when data adjusted for stimulus were considered.

### Vascular reactivity: Age- and sex-specific reference intervals

There were VR indexes: (i) that required sex-specific RIs from certain ages (e.g., males and females <60.1 y did not show differences in pFMDv [p˃0.05; European Criteria subgroup], while for older ages, pFMDv was gradually higher in males), (ii) for which sex-specific RIs were necessary disregard of age (e.g., ΔWSS_*Peak_Basal*_), (iii) for which sex-specific RIs were not necessary (e.g., FMD%) and (iv) that did not require age and sex-specific RIs (e.g., TPD_FMD%) [S7 and S8 Tables in S1 File].

### Age- and/or sex-related reference intervals

Data for year-by-year RIs are in (S10-S35 Tables in S1 File for ´European Criteria´; S36-S63 Tables in S1 File for ´HUNT-FIT criteria´ RIs subgroup). S2 File shows age-related percentile curves (all, females and males) for the different VR indexes.

**Fig 9:**
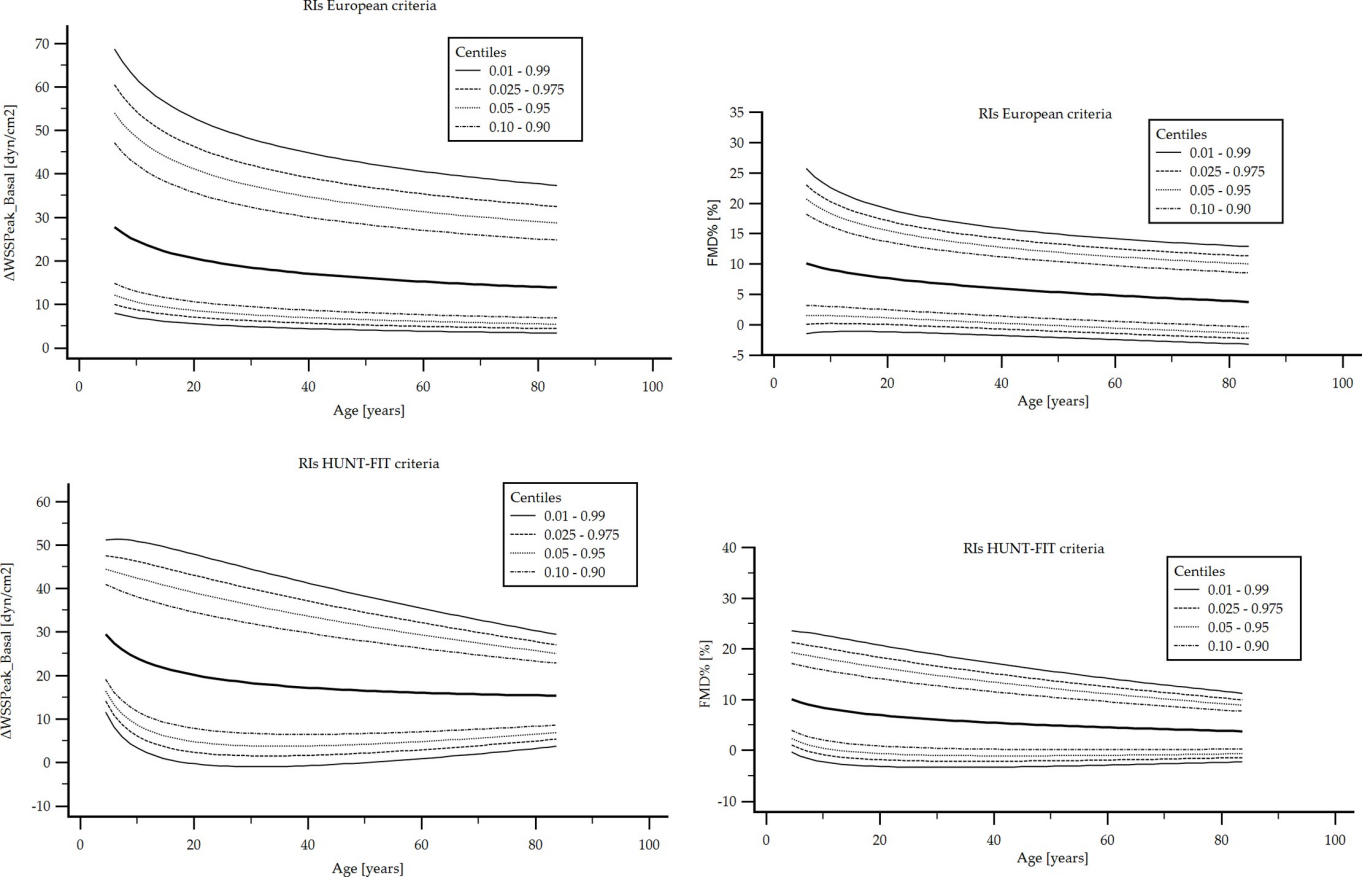
FMD% (top) and ΔWSS_Peak_Basal_ (bottom) age-related profiles or percentile curves for European (left) and HUNT-FIT (right) criteria. Quantitative data is in Table 6 and S1 File.

Fig 9 exemplifies age-related profiles or percentile curves obtained. Table 6 summarizes (10 y intervals) reference values (p2.5th, p10th, p25th, p50th, p75th, p90th, p97.5th) for VR indexes obtained for RIs-subgroups defined following the European and HUNT-FIT criteria.

**Table 6:** Vascular reactivity indexes RIs (European and HUNT-FIT criteria; detailed by decade of life).

	European Criteria							HUNT-FIT Criteria						
	**Percentiles**													
**Age [year]**	2.5th	10th	25th	**50th**	75th	90th	97.5th	2.5th	10th	25th	**50th**	75th	90th	97.5th
**ΔDD Peak_Basal [mm]: All**														
6	-0.04	0.05	0.14	**0.23**	0.33	0.43	0.53	-0.01	0.08	0.16	**0.26**	0.36	0.45	0.56
10	0.00	0.08	0.16	**0.25**	0.34	0.42	0.52	-0.03	0.06	0.15	**0.24**	0.35	0.44	0.55
20	0.01	0.09	0.17	**0.25**	0.34	0.42	0.51	-0.05	0.04	0.12	**0.22**	0.33	0.43	0.54
30	0.00	0.08	0.16	**0.24**	0.33	0.41	0.51	-0.07	0.02	0.11	**0.21**	0.32	0.42	0.53
40	-0.02	0.06	0.14	**0.23**	0.32	0.41	0.51	-0.08	0.02	0.10	**0.21**	0.31	0.41	0.53
50	-0.05	0.04	0.12	**0.22**	0.32	0.41	0.51	-0.09	0.01	0.10	**0.20**	0.31	0.41	0.52
60	-0.08	0.02	0.10	**0.20**	0.31	0.40	0.51	-0.09	0.00	0.09	**0.19**	0.30	0.40	0.52
70	-0.11	-0.01	0.08	**0.19**	0.30	0.40	0.52	-0.10	0.00	0.09	**0.19**	0.30	0.40	0.52
80	-0.13	-0.03	0.06	**0.18**	0.29	0.40	0.52	-0.10	-0.01	0.08	**0.19**	0.30	0.40	0.51
84	-0.14	-0.04	0.06	**0.17**	0.29	0.40	0.52	-0.10	-0.01	0.08	**0.19**	0.29	0.40	0.51
**DD Ratio Peak_Basal [%]: All**														
6	98.8	102.8	106.3	**110.3**	114.2	117.8	121.9	99.6	103.0	106.1	**109.6**	113.3	116.7	120.6
10	99.3	102.7	105.9	**109.4**	112.9	116.0	119.6	98.6	101.9	105.1	**108.6**	112.3	115.8	119.7
20	99.4	102.3	105.0	**108.0**	110.9	113.6	116.6	97.6	100.8	103.8	**107.2**	110.8	114.1	117.8
30	99.1	101.8	104.3	**107.0**	109.7	112.2	115.0	97.3	100.4	103.2	**106.4**	109.7	112.7	116.2
40	98.8	101.4	103.7	**106.3**	108.9	111.2	113.8	97.4	100.2	102.8	**105.7**	108.8	111.6	114.8
50	98.4	100.9	103.1	**105.6**	108.2	110.4	113.0	97.5	100.1	102.5	**105.2**	108.0	110.5	113.5
60	98.0	100.4	102.6	**105.1**	107.6	109.8	112.3	97.7	100.1	102.3	**104.7**	107.3	109.6	112.3
70	97.6	100.0	102.2	**104.6**	107.1	109.3	111.7	97.9	100.1	102.1	**104.4**	106.7	108.8	111.2
80	97.2	99.6	101.7	**104.2**	106.6	108.8	111.3	98.2	100.2	102.0	**104.0**	106.1	108.0	110.2
84	97.0	99.4	101.6	**104.0**	106.4	108.6	111.1	98.3	100.2	101.9	**103.9**	105.9	107.7	109.8
**FMD% [%]: All**														
6	0.14	3.22	6.28	**10.01**	14.07	17.99	22.67	0.27	3.16	6.01	**9.46**	13.19	16.77	21.03
10	0.26	3.05	5.79	**9.11**	12.70	16.15	20.25	-0.84	2.05	4.91	**8.41**	12.21	15.89	20.28
20	0.07	2.49	4.85	**7.69**	10.74	13.66	17.12	-1.83	0.90	3.62	**6.96**	10.61	14.14	18.37
30	-0.28	1.96	4.13	**6.74**	9.54	12.22	15.39	-2.10	0.45	2.99	**6.09**	9.48	12.76	16.68
40	-0.66	1.46	3.53	**6.00**	8.66	11.20	14.20	-2.12	0.25	2.60	**5.47**	8.58	11.59	15.17
50	-1.04	1.01	3.00	**5.39**	7.95	10.40	13.30	-2.03	0.17	2.34	**4.97**	7.82	10.56	13.82
60	-1.41	0.58	2.53	**4.86**	7.36	9.75	12.59	-1.88	0.16	2.16	**4.56**	7.15	9.63	12.58
70	-1.76	0.19	2.10	**4.38**	6.85	9.20	12.00	-1.69	0.19	2.02	**4.21**	6.55	8.79	11.44
80	-2.09	-0.18	1.70	**3.96**	6.39	8.73	11.49	-1.47	0.25	1.92	**3.90**	6.01	8.02	10.38
84	-2.22	-0.32	1.55	**3.80**	6.23	8.55	11.31	-1.38	0.29	1.89	**3.79**	5.81	7.72	9.98
**TPD FMD% [s]: All**														
6	23.2	41.5	61.8	**88.5**	119.5	150.7	189.4	32.6	49.5	67.6	**91.0**	118.1	145.4	179.3
10	18.3	37.3	59.2	**88.7**	123.4	158.8	203.1	22.2	40.2	60.8	**88.7**	122.0	156.4	200.1
20	15.7	34.8	57.2	**87.9**	124.3	161.7	208.7	15.5	33.4	55.1	**85.8**	123.2	162.7	213.4
30	16.5	35.2	57.1	**86.7**	121.8	157.7	202.8	15.1	32.6	54.0	**84.2**	121.0	159.9	209.8
40	18.5	36.7	57.6	**85.5**	118.3	151.8	193.6	16.6	33.8	54.4	**83.1**	117.9	154.4	201.1
50	21.0	38.6	58.3	**84.3**	114.6	145.2	183.3	19.1	35.9	55.5	**82.3**	114.4	147.8	190.3
60	23.9	40.8	59.2	**83.2**	110.8	138.5	172.7	22.2	38.5	56.9	**81.6**	110.8	140.9	178.9
70	27.1	43.2	60.2	**82.1**	107.0	131.8	162.2	25.9	41.5	58.6	**81.1**	107.3	134.0	167.4
80	30.6	45.6	61.2	**81.0**	103.3	125.2	152.1	30.1	44.8	60.5	**80.7**	103.9	127.2	156.1
84	32.0	46.6	61.7	**80.6**	101.8	122.7	148.1	31.8	46.2	61.2	**80.5**	102.5	124.5	151.7
**ΔDD Peak-Basal/ΔVPeak-Basal [mm/cm/s]: All**														
6	1.8(-3)	3.7(-3)	5.6(-3)	**7.8(-3)**	1.0(-2)	1.2(-2)	1.5(-2)	-4.0(-3)	4.2(-4)	4.6(-3)	**9.4(-3)**	1.5(-2)	1.9(-2)	2.5(-2)
10	4.8(-4)	3.2(-3)	5.9(-3)	**9.2(-3)**	1.3(-2)	1.7(-2)	2.1(-2)	-4.3(-3)	1.4(-4)	4.3(-3)	**9.2(-3)**	1.4(-2)	1.9(-2)	2.5(-2)
20	-1.0(-3)	2.3(-3)	5.8(-3)	**1.0(-2)**	1.5(-2)	2.0(-2)	2.7(-2)	-4.7(-3)	-2.3(-4)	4.0(-3)	**9.0(-3)**	1.4(-2)	1.9(-2)	2.5(-2)
30	-1.8(-3)	1.8(-3)	5.4(-3)	**1.0(-2)**	1.6(-2)	2.1(-2)	2.8(-2)	-4.9(-3)	-4.5(-4)	3.8(-3)	**8.8(-3)**	1.4(-2)	1.9(-2)	2.5(-2)
40	-2.2(-3)	1.3(-3)	5.0(-3)	**9.7(-3)**	1.5(-2)	2.1(-2)	2.8(-2)	-5.1(-3)	-6.1(-4)	3.7(-3)	**8.7(-3)**	1.4(-2)	1.9(-2)	2.5(-2)
50	-2.5(-3)	9.2(-4)	4.5(-3)	**9.1(-3)**	1.4(-2)	2.0(-2)	2.7(-2)	-5.2(-3)	-7.3(-4)	3.6(-3)	**8.6(-3)**	1.4(-2)	1.9(-2)	2.5(-2)
60	-2.7(-3)	5.8(-4)	4.0(-3)	**8.4(-3)**	1.3(-2)	1.9(-2)	2.5(-2)	-5.3(-3)	-8.3(-4)	3.5(-3)	**8.5(-3)**	1.4(-2)	1.9(-2)	2.5(-2)
70	-2.8(-3)	2.9(-4)	3.5(-3)	**7.7(-3)**	1.2(-2)	1.7(-2)	2.3(-2)	-5.4(-3)	-9.1(-4)	3.4(-3)	**8.5(-3)**	1.4(-2)	1.9(-2)	2.5(-2)
80	-2.9(-3)	1.8(-5)	3.1(-3)	**6.9(-3)**	1.1(-2)	1.6(-2)	2.2(-2)	-5.5(-3)	-9.8(-4)	3.3(-3)	**8.4(-3)**	1.4(-2)	1.9(-2)	2.5(-2)
84	-3.0(-3)	-8.3(-5)	2.9(-3)	**6.7(-3)**	1.1(-2)	1.5(-2)	2.1(-2)	-5.5(-3)	-1.0(-3)	3.3(-3)	**8.4(-3)**	1.4(-2)	1.9(-2)	2.5(-2)
**FMD/ΔVPeak_Basal [1/cm/s]: All**														
6	3.6(-4)	1.4(-3)	2.3(-3)	**3.4(-3)**	4.5(-3)	5.6(-3)	6.8(-3)	-6.3(-4)	5.6(-4)	1.7(-3)	**3.1(-3)**	4.7(-3)	6.2(-3)	8.0(-3)
10	-2.3(-4)	9.7(-4)	2.1(-3)	**3.4(-3)**	4.8(-3)	6.2(-3)	7.7(-3)	-1.1(-3)	2.1(-4)	1.5(-3)	**3.1(-3)**	4.8(-3)	6.5(-3)	8.6(-3)
20	-8.0(-4)	5.2(-4)	1.8(-3)	**3.3(-3)**	4.8(-3)	6.3(-3)	8.1(-3)	-1.4(-3)	-1.1(-4)	1.2(-3)	**2.8(-3)**	4.6(-3)	6.4(-3)	8.6(-3)
30	-9.9(-4)	3.1(-4)	1.5(-3)	**3.0(-3)**	4.5(-3)	6.0(-3)	7.7(-3)	-1.5(-3)	-2.1(-4)	1.0(-3)	**2.6(-3)**	4.3(-3)	6.0(-3)	8.0(-3)
40	-1.0(-3)	1.9(-4)	1.4(-3)	**2.7(-3)**	4.2(-3)	5.6(-3)	7.2(-3)	-1.4(-3)	-2.3(-4)	9.4(-4)	**2.4(-3)**	3.9(-3)	5.5(-3)	7.4(-3)
50	-1.0(-3)	1.1(-4)	1.2(-3)	**2.5(-3)**	3.8(-3)	5.0(-3)	6.5(-3)	-1.3(-3)	-2.1(-4)	8.6(-4)	**2.2(-3)**	3.6(-3)	5.0(-3)	6.7(-3)
60	-9.9(-4)	6.2(-5)	1.0(-3)	**2.2(-3)**	3.4(-3)	4.5(-3)	5.9(-3)	-1.2(-3)	-1.7(-4)	8.0(-4)	**2.0(-3)**	3.3(-3)	4.5(-3)	6.0(-3)
70	-9.2(-4)	3.0(-5)	9.1(-4)	**1.9(-3)**	3.0(-3)	4.0(-3)	5.2(-3)	-1.0(-3)	-1.2(-4)	7.6(-4)	**1.8(-3)**	2.9(-3)	4.0(-3)	5.3(-3)
80	-8.3(-4)	1.1(-5)	7.9(-4)	**1.7(-3)**	2.6(-3)	3.5(-3)	4.5(-3)	-8.8(-4)	-6.1(-5)	7.2(-4)	**1.6(-3)**	2.6(-3)	3.6(-3)	4.7(-3)
84	-7.9(-4)	6.0(-6)	7.5(-4)	**1.6(-3)**	2.5(-3)	3.3(-3)	4.3(-3)	-8.1(-4)	-3.5(-5)	7.1(-4)	**1.6(-3)**	2.5(-3)	3.4(-3)	4.4(-3)
**pFMDv: All**														
6	1.9(-3)	1.2(-2)	2.6(-2)	**4.9(-2)**	8.1(-2)	1.2(-1)	1.9(-1)	-5.5(-2)	-2.7(-2)	-1.3(-3)	**2.9(-2)**	6.0(-2)	8.9(-2)	1.2(-1)
10	9.8(-4)	9.9(-3)	2.1(-2)	**3.9(-2)**	6.4(-2)	9.5(-2)	1.4(-1)	-6.0(-2)	-3.0(-2)	-1.6(-3)	**3.1(-2)**	6.6(-2)	9.8(-2)	1.4(-1)
20	-2.6(-3)	4.4(-3)	1.3(-2)	**2.7(-2)**	4.8(-2)	7.2(-2)	1.1(-1)	-6.0(-2)	-3.0(-2)	-1.6(-3)	**3.2(-2)**	6.6(-2)	9.9(-2)	1.4(-1)
30	-5.7(-3)	3.3(-4)	8.3(-3)	**2.1(-2)**	4.1(-2)	6.5(-2)	1.0(-1)	-5.6(-2)	-2.8(-2)	-1.4(-3)	**2.9(-2)**	6.1(-2)	9.1(-2)	1.3(-1)
40	-7.9(-3)	-2.7(-3)	4.7(-3)	**1.7(-2)**	3.7(-2)	6.2(-2)	1.0(-1)	-5.0(-2)	-2.5(-2)	-1.1(-3)	**2.6(-2)**	5.5(-2)	8.1(-2)	1.1(-1)
50	-9.6(-3)	-5.0(-3)	2.0(-3)	**1.4(-2)**	3.4(-2)	6.2(-2)	1.1(-1)	-4.4(-2)	-2.2(-2)	-7.2(-4)	**2.3(-2)**	4.8(-2)	7.1(-2)	9.7(-2)
60	-1.1(-2)	-6.8(-3)	-2.0(-4)	**1.2(-2)**	3.3(-2)	6.3(-2)	1.1(-1)	-3.8(-2)	-1.8(-2)	-3.7(-4)	**2.0(-2)**	4.1(-2)	6.1(-2)	8.3(-2)
70	-1.2(-2)	-8.2(-3)	-2.0(-3)	**1.0(-2)**	3.2(-2)	6.4(-2)	1.2(-1)	-3.1(-2)	-1.5(-2)	-2.1(-5)	**1.7(-2)**	3.4(-2)	5.0(-2)	6.8(-2)
80	-1.3(-2)	-9.3(-3)	-3.5(-3)	**8.8(-3)**	3.1(-2)	6.6(-2)	1.3(-1)	-2.5(-2)	-1.2(-2)	3.2(-4)	**1.4(-2)**	2.7(-2)	4.0(-2)	5.4(-2)
84	-1.3(-2)	-9.7(-3)	-4.0(-3)	**8.2(-3)**	3.1(-2)	6.7(-2)	1.3(-1)	-2.2(-2)	-1.0(-2)	4.6(-4)	**1.3(-2)**	2.5(-2)	3.6(-2)	4.9(-2)
**FMD% WSS: All**														
6	1.2(-3)	1.3(-2)	2.9(-2)	**5.7(-2)**	1.0(-1)	1.6(-1)	2.7(-1)	-6.8(-2)	-3.2(-2)	2.7(-3)	**4.4(-2)**	8.7(-2)	1.3(-1)	1.8(-1)
10	8.4(-4)	1.0(-2)	2.3(-2)	**4.4(-2)**	7.7(-2)	1.2(-1)	1.9(-1)	-6.3(-2)	-3.0(-2)	1.6(-3)	**3.9(-2)**	7.8(-2)	1.1(-1)	1.6(-1)
20	-2.5(-3)	4.8(-3)	1.4(-2)	**3.0(-2)**	5.4(-2)	8.5(-2)	1.3(-1)	-5.5(-2)	-2.7(-2)	5.3(-4)	**3.2(-2)**	6.5(-2)	9.6(-2)	1.3(-1)
30	-5.7(-3)	4.3(-4)	8.9(-3)	**2.3(-2)**	4.5(-2)	7.5(-2)	1.2(-1)	-5.0(-2)	-2.4(-2)	1.2(-4)	**2.8(-2)**	5.7(-2)	8.5(-2)	1.2(-1)
40	-8.1(-3)	-2.8(-3)	4.9(-3)	**1.9(-2)**	4.1(-2)	7.2(-2)	1.3(-1)	-4.6(-2)	-2.2(-2)	-2.7(-5)	**2.5(-2)**	5.2(-2)	7.6(-2)	1.0(-1)
50	-9.9(-3)	-5.3(-3)	1.9(-3)	**1.5(-2)**	3.8(-2)	7.2(-2)	1.3(-1)	-4.2(-2)	-2.0(-2)	-5.4(-5)	**2.3(-2)**	4.8(-2)	7.0(-2)	9.6(-2)
60	-1.1(-2)	-7.2(-3)	-4.7(-4)	**1.3(-2)**	3.7(-2)	7.4(-2)	1.4(-1)	-3.9(-2)	-1.9(-2)	-9.8(-6)	**2.2(-2)**	4.4(-2)	6.5(-2)	8.8(-2)
70	-1.2(-2)	-8.6(-3)	-2.4(-3)	**1.1(-2)**	3.6(-2)	7.7(-2)	1.6(-1)	-3.6(-2)	-1.7(-2)	7.9(-5)	**2.0(-2)**	4.1(-2)	6.0(-2)	8.2(-2)
80	-1.3(-2)	-9.8(-3)	-4.0(-3)	**9.1(-3)**	3.6(-2)	8.1(-2)	1.8(-1)	-3.4(-2)	-1.6(-2)	2.0(-4)	**1.9(-2)**	3.8(-2)	5.6(-2)	7.6(-2)
84	-1.3(-2)	-1.0(-2)	-4.5(-3)	**8.5(-3)**	3.5(-2)	8.2(-2)	1.8(-1)	-3.3(-2)	-1.6(-2)	2.5(-4)	**1.8(-2)**	3.7(-2)	5.4(-2)	7.4(-2)
**ΔD Prerelease_Basal [mm]: All**														
6	-0.09	-0.01	0.05	**0.13**	0.21	0.28	0.37	-0.17	-0.07	0.02	**0.12**	0.23	0.32	0.43
10	-0.11	-0.03	0.05	**0.14**	0.23	0.31	0.41	-0.18	-0.08	0.01	**0.12**	0.24	0.34	0.46
20	-0.14	-0.05	0.04	**0.14**	0.24	0.34	0.45	-0.20	-0.10	0.01	**0.12**	0.24	0.35	0.48
30	-0.16	-0.07	0.02	**0.13**	0.24	0.34	0.46	-0.21	-0.10	0.00	**0.12**	0.24	0.35	0.48
40	-0.18	-0.08	0.01	**0.12**	0.23	0.34	0.46	-0.22	-0.11	-0.01	**0.11**	0.23	0.34	0.47
50	-0.19	-0.09	0.00	**0.11**	0.22	0.33	0.45	-0.22	-0.12	-0.02	**0.10**	0.22	0.33	0.45
60	-0.20	-0.10	-0.01	**0.10**	0.21	0.32	0.44	-0.22	-0.12	-0.02	**0.09**	0.20	0.31	0.43
70	-0.21	-0.11	-0.02	**0.09**	0.20	0.31	0.43	-0.23	-0.12	-0.03	**0.08**	0.19	0.30	0.42
80	-0.22	-0.12	-0.03	**0.08**	0.19	0.29	0.42	-0.23	-0.13	-0.03	**0.07**	0.18	0.28	0.40
84	-0.22	-0.12	-0.03	**0.07**	0.19	0.29	0.41	-0.23	-0.13	-0.04	**0.07**	0.18	0.28	0.39
**DD Ratio Prerelease_Basal [%]: All**														
6	95.4	98.8	102.0	**105.6**	109.4	112.9	116.9	95.0	97.8	100.7	**104.2**	108.3	112.6	118.2
10	95.5	98.7	101.7	**105.2**	108.7	112.0	115.9	94.8	97.6	100.4	**104.0**	108.1	112.3	117.9
20	95.4	98.4	101.2	**104.4**	107.7	110.7	114.2	94.7	97.4	100.1	**103.5**	107.3	111.3	116.4
30	95.3	98.2	100.8	**103.8**	106.9	109.8	113.1	94.7	97.3	99.8	**103.0**	106.6	110.2	114.9
40	95.1	97.9	100.4	**103.3**	106.3	109.0	112.2	94.8	97.2	99.6	**102.6**	105.9	109.2	113.5
50	95.0	97.7	100.1	**102.9**	105.8	108.4	111.5	94.9	97.2	99.5	**102.2**	105.3	108.3	112.2
60	94.8	97.4	99.8	**102.5**	105.3	107.9	110.8	95.0	97.2	99.3	**101.9**	104.7	107.5	111.0
70	94.7	97.2	99.5	**102.2**	104.9	107.4	110.2	95.2	97.2	99.2	**101.6**	104.2	106.8	109.9
80	94.6	97.0	99.3	**101.9**	104.5	106.9	109.7	95.3	97.2	99.1	**101.3**	103.7	106.0	108.9
84	94.5	96.9	99.2	**101.7**	104.4	106.8	109.5	95.4	97.2	99.0	**101.2**	103.5	105.8	108.5
**L-FMC% [%]: All**														
6	-3.82	-1.00	1.85	**5.38**	9.28	13.08	17.67	-5.68	-2.44	0.72	**4.52**	8.60	12.49	17.10
10	-3.82	-1.14	1.58	**4.92**	8.61	12.20	16.51	-5.85	-2.62	0.52	**4.31**	8.37	12.26	16.85
20	-3.95	-1.45	1.05	**4.13**	7.50	10.78	14.72	-5.90	-2.82	0.18	**3.77**	7.62	11.29	15.64
30	-4.09	-1.73	0.64	**3.55**	6.73	9.82	13.52	-5.80	-2.90	-0.09	**3.28**	6.87	10.29	14.33
40	-4.24	-1.97	0.29	**3.07**	6.11	9.05	12.58	-5.67	-2.94	-0.30	**2.83**	6.17	9.35	13.08
50	-4.38	-2.20	-0.01	**2.66**	5.58	8.41	11.80	-5.52	-2.95	-0.49	**2.43**	5.53	8.46	11.91
60	-4.52	-2.40	-0.29	**2.30**	5.12	7.85	11.13	-5.36	-2.95	-0.66	**2.05**	4.92	7.63	10.81
70	-4.65	-2.59	-0.54	**1.97**	4.70	7.35	10.53	-5.20	-2.95	-0.81	**1.70**	4.35	6.85	9.78
80	-4.77	-2.77	-0.77	**1.66**	4.32	6.90	9.98	-5.04	-2.94	-0.96	**1.37**	3.82	6.12	8.80
84	-4.82	-2.84	-0.86	**1.55**	4.18	6.73	9.78	-4.97	-2.94	-1.01	**1.24**	3.61	5.83	8.42
**ΔDD Prerelease_Basal/ΔEDV Prelease_Basal: All**														
6	-0.10	-0.05	0.01	**0.09**	0.18	0.27	0.39	-0.18	-0.11	-0.03	**0.06**	0.17	0.28	0.41
10	-0.10	-0.05	0.00	**0.06**	0.13	0.21	0.30	-0.14	-0.09	-0.03	**0.03**	0.10	0.17	0.25
20	-0.10	-0.06	-0.02	**0.02**	0.08	0.13	0.19	-0.09	-0.06	-0.03	**0.00**	0.03	0.06	0.10
30	-0.09	-0.06	-0.03	**0.01**	0.05	0.09	0.14	-0.07	-0.05	-0.03	**-0.01**	0.01	0.03	0.05
40	-0.09	-0.06	-0.04	**-0.01**	0.03	0.06	0.10	-0.07	-0.05	-0.03	**-0.01**	0.00	0.02	0.04
50	-0.09	-0.07	-0.04	**-0.02**	0.01	0.04	0.07	-0.07	-0.05	-0.03	**-0.01**	0.01	0.02	0.04
60	-0.09	-0.07	-0.05	**-0.02**	0.00	0.03	0.05	-0.07	-0.05	-0.03	**-0.01**	0.01	0.03	0.05
70	-0.09	-0.07	-0.05	**-0.03**	-0.01	0.01	0.04	-0.08	-0.06	-0.03	**-0.01**	0.02	0.04	0.07
80	-0.09	-0.07	-0.05	**-0.04**	-0.02	0.00	0.02	-0.09	-0.06	-0.04	**0.00**	0.03	0.06	0.09
84	-0.08	-0.07	-0.05	**-0.04**	-0.02	0.00	0.02	-0.09	-0.06	-0.04	**0.00**	0.03	0.06	0.10
**L-FMC/ΔV Prerelease_Basal [1/cm/s]: All**														
6	-0.03	-0.02	0.00	**0.03**	0.07	0.11	0.16	-0.05	-0.03	-0.01	**0.02**	0.06	0.10	0.15
10	-0.03	-0.02	0.00	**0.02**	0.05	0.08	0.12	-0.04	-0.03	-0.01	**0.01**	0.03	0.06	0.09
20	-0.03	-0.02	-0.01	**0.01**	0.03	0.05	0.07	-0.03	-0.02	-0.01	**0.00**	0.01	0.02	0.04
30	-0.03	-0.02	-0.01	**0.00**	0.02	0.03	0.05	-0.02	-0.02	-0.01	**0.00**	0.00	0.01	0.02
40	-0.03	-0.02	-0.01	**0.00**	0.01	0.02	0.03	-0.02	-0.01	-0.01	**0.00**	0.00	0.01	0.01
50	-0.03	-0.02	-0.01	**-0.01**	0.00	0.01	0.02	-0.02	-0.01	-0.01	**0.00**	0.00	0.01	0.01
60	-0.03	-0.02	-0.01	**-0.01**	0.00	0.01	0.02	-0.02	-0.01	-0.01	**0.00**	0.00	0.01	0.01
70	-0.02	-0.02	-0.02	**-0.01**	0.00	0.00	0.01	-0.02	-0.01	-0.01	**0.00**	0.00	0.01	0.02
80	-0.02	-0.02	-0.02	**-0.01**	-0.01	0.00	0.01	-0.02	-0.01	-0.01	**0.00**	0.01	0.01	0.02
84	-0.02	-0.02	-0.02	**-0.01**	-0.01	0.00	0.00	-0.02	-0.01	-0.01	**0.00**	0.01	0.02	0.03
**pL-FMCv: All**														
6	-0.13	-0.09	-0.04	**0.03**	0.12	0.21	0.32	-0.14	-0.09	-0.05	**0.00**	0.05	0.11	0.17
10	-0.12	-0.08	-0.04	**0.02**	0.09	0.17	0.26	-0.15	-0.10	-0.05	**0.01**	0.07	0.12	0.19
20	-0.11	-0.08	-0.04	**0.00**	0.06	0.11	0.19	-0.15	-0.10	-0.05	**0.01**	0.07	0.13	0.20
30	-0.11	-0.08	-0.05	**-0.01**	0.04	0.09	0.14	-0.14	-0.09	-0.05	**0.00**	0.06	0.11	0.18
40	-0.10	-0.08	-0.05	**-0.01**	0.03	0.07	0.12	-0.13	-0.09	-0.05	**0.00**	0.05	0.09	0.15
50	-0.10	-0.08	-0.05	**-0.02**	0.02	0.05	0.10	-0.12	-0.09	-0.05	**-0.01**	0.03	0.07	0.12
60	-0.10	-0.08	-0.05	**-0.02**	0.01	0.04	0.08	-0.11	-0.08	-0.05	**-0.02**	0.02	0.05	0.09
70	-0.10	-0.07	-0.05	**-0.03**	0.00	0.03	0.07	-0.11	-0.08	-0.05	**-0.02**	0.01	0.04	0.07
80	-0.09	-0.07	-0.05	**-0.03**	0.00	0.02	0.06	-0.10	-0.07	-0.05	**-0.03**	-0.01	0.02	0.04
84	-0.09	-0.07	-0.05	**-0.03**	0.00	0.02	0.05	-0.09	-0.07	-0.05	**-0.03**	-0.01	0.01	0.03
**L-FMC%/WSS: All**														
6	-75.06	-48.58	-27.19	**-4.87**	16.35	34.72	54.63	-46.66	-29.46	-15.71	**-1.63**	11.49	22.63	34.50
10	-59.23	-38.99	-22.04	**-4.07**	13.20	28.23	44.61	-37.90	-24.30	-13.11	**-1.46**	9.52	18.93	29.02
20	-39.55	-26.46	-15.18	**-2.98**	8.89	19.31	30.75	-26.90	-17.56	-9.65	**-1.24**	6.82	13.81	21.37
30	-28.66	-19.35	-11.21	**-2.35**	6.35	14.03	22.49	-20.83	-13.75	-7.66	**-1.11**	5.23	10.76	16.78
40	-21.14	-14.38	-8.43	**-1.90**	4.54	10.25	16.56	-16.67	-11.09	-6.25	**-1.01**	4.09	8.57	13.47
50	-15.42	-10.57	-6.28	**-1.55**	3.13	7.29	11.91	-13.51	-9.06	-5.17	**-0.94**	3.20	6.86	10.87
60	-10.80	-7.48	-4.53	**-1.27**	1.97	4.86	8.08	-10.98	-7.42	-4.30	**-0.88**	2.48	5.44	8.71
70	-6.94	-4.88	-3.05	**-1.02**	1.00	2.80	4.82	-8.86	-6.04	-3.56	**-0.83**	1.86	4.24	6.88
80	-3.62	-2.65	-1.78	**-0.82**	0.15	1.01	1.97	-7.04	-4.86	-2.92	**-0.79**	1.32	3.20	5.27
84	-2.42	-1.84	-1.32	**-0.74**	-0.16	0.35	0.93	-6.39	-4.42	-2.69	**-0.77**	1.12	2.81	4.69
**TVR [%]: All**														
6	-4.82	-1.47	1.52	**4.82**	8.12	11.07	14.37	-5.62	-1.82	1.50	**5.13**	8.69	11.85	15.33
10	-4.42	-1.39	1.31	**4.30**	7.29	9.96	12.95	-5.61	-2.10	0.97	**4.33**	7.62	10.55	13.78
20	-3.87	-1.28	1.03	**3.60**	6.16	8.45	11.01	-5.32	-2.24	0.46	**3.41**	6.32	8.90	11.76
30	-3.56	-1.21	0.87	**3.19**	5.50	7.57	9.88	-4.96	-2.16	0.31	**3.00**	5.67	8.03	10.64
40	-3.33	-1.17	0.76	**2.90**	5.03	6.94	9.08	-4.60	-2.00	0.28	**2.79**	5.26	7.46	9.89
50	-3.16	-1.13	0.67	**2.67**	4.66	6.46	8.46	-4.25	-1.82	0.32	**2.66**	4.98	7.05	9.33
60	-3.01	-1.11	0.59	**2.48**	4.37	6.06	7.95	-3.92	-1.63	0.38	**2.60**	4.79	6.74	8.89
70	-2.89	-1.08	0.53	**2.33**	4.12	5.72	7.51	-3.60	-1.43	0.47	**2.57**	4.65	6.49	8.54
80	-2.79	-1.06	0.48	**2.19**	3.90	5.43	7.14	-3.29	-1.24	0.58	**2.57**	4.54	6.30	8.25
84	-2.75	-1.05	0.46	**2.14**	3.82	5.32	7.00	-3.17	-1.16	0.62	**2.57**	4.51	6.23	8.14
**TVR [%]: Female**														
6	-3.32	-0.29	2.52	**5.75**	9.06	12.10	15.56	-2.85	0.53	3.46	**6.64**	9.75	12.49	15.49
10	-3.31	-0.50	2.11	**5.10**	8.16	10.97	14.16	-4.46	-0.92	2.14	**5.44**	8.67	11.50	14.62
20	-3.29	-0.77	1.56	**4.22**	6.94	9.44	12.28	-5.75	-2.21	0.84	**4.14**	7.35	10.17	13.27
30	-3.28	-0.93	1.23	**3.71**	6.24	8.55	11.19	-5.87	-2.48	0.44	**3.61**	6.69	9.40	12.37
40	-3.27	-1.05	1.00	**3.34**	5.74	7.93	10.42	-5.59	-2.40	0.37	**3.36**	6.28	8.85	11.67
50	-3.27	-1.13	0.83	**3.07**	5.35	7.45	9.82	-5.14	-2.15	0.44	**3.26**	6.00	8.42	11.08
60	-3.26	-1.21	0.68	**2.84**	5.04	7.05	9.34	-4.59	-1.81	0.61	**3.24**	5.81	8.08	10.57
70	-3.26	-1.27	0.56	**2.65**	4.77	6.72	8.93	-4.01	-1.43	0.82	**3.27**	5.67	7.78	10.11
80	-3.25	-1.32	0.46	**2.48**	4.55	6.43	8.58	-3.39	-1.01	1.07	**3.34**	5.56	7.53	9.70
84	-3.25	-1.34	0.42	**2.42**	4.46	6.33	8.45	-3.15	-0.84	1.17	**3.37**	5.53	7.44	9.54
**TVR [%]: Male**														
6	-5.96	-2.38	0.73	**4.12**	7.44	10.38	13.62	-7.66	-3.79	-0.35	**3.46**	7.25	10.64	14.43
10	-5.19	-2.05	0.70	**3.69**	6.63	9.24	12.12	-6.50	-3.07	-0.02	**3.36**	6.73	9.74	13.11
20	-4.16	-1.60	0.65	**3.11**	5.53	7.68	10.06	-5.06	-2.24	0.27	**3.06**	5.84	8.33	11.10
30	-3.56	-1.34	0.62	**2.76**	4.88	6.77	8.85	-4.30	-1.85	0.34	**2.76**	5.18	7.35	9.78
40	-3.14	-1.15	0.60	**2.52**	4.42	6.11	7.99	-3.82	-1.63	0.32	**2.49**	4.65	6.59	8.75
50	-2.81	-1.01	0.58	**2.33**	4.06	5.61	7.31	-3.47	-1.49	0.27	**2.23**	4.18	5.94	7.89
60	-2.54	-0.89	0.57	**2.18**	3.77	5.19	6.76	-3.22	-1.41	0.20	**1.98**	3.77	5.37	7.15
70	-2.32	-0.79	0.56	**2.05**	3.52	4.84	6.30	-3.02	-1.36	0.11	**1.75**	3.39	4.86	6.49
80	-2.12	-0.70	0.55	**1.94**	3.31	4.53	5.89	-2.86	-1.34	0.02	**1.53**	3.04	4.39	5.90
84	-2.05	-0.67	0.55	**1.89**	3.23	4.42	5.74	-2.80	-1.33	-0.01	**1.45**	2.90	4.21	5.67
**ΔV Peak_Basal [cm/s]: Male**														
6	11.31	16.16	16.16	**16.16**	16.16	43.35	52.99	11.10	15.73	20.12	**25.24**	30.62	35.65	41.48
10	10.54	15.33	15.33	**15.33**	15.33	42.61	52.37	8.13	13.92	19.51	**26.14**	33.18	39.83	47.61
20	9.54	14.24	14.24	**14.24**	14.24	41.61	51.52	5.52	12.16	18.70	**26.57**	35.01	43.06	52.53
30	8.98	13.62	13.62	**13.62**	13.62	41.03	51.03	4.86	11.59	18.24	**26.28**	34.91	43.15	52.87
40	8.59	13.19	13.19	**13.19**	13.19	40.62	50.68	4.85	11.43	17.93	**25.76**	34.18	42.20	51.66
50	8.30	12.86	12.86	**12.86**	12.86	40.30	50.42	5.15	11.48	17.69	**25.16**	33.17	40.79	49.77
60	8.06	12.59	12.59	**12.59**	12.59	40.05	50.20	5.62	11.63	17.50	**24.53**	32.04	39.17	47.56
70	7.87	12.37	12.37	**12.37**	12.37	39.83	50.01	6.20	11.85	17.34	**23.88**	30.85	37.45	45.19
80	7.70	12.18	12.18	**12.18**	12.18	39.64	49.86	6.84	12.12	17.20	**23.24**	29.63	35.68	42.75
84	7.64	12.11	12.11	**12.11**	12.11	39.58	49.80	7.12	12.24	17.16	**22.98**	29.15	34.97	41.77
**ΔWSS Peak_Basal [dyn/cm**^**2**^**]: All**														
6	10.08	14.85	20.29	**27.84**	37.18	47.29	60.73	10.55	15.92	21.10	**27.27**	33.83	40.04	47.35
10	8.71	12.94	17.80	**24.57**	32.98	42.10	54.27	6.03	11.68	17.23	**23.94**	31.18	38.10	46.30
20	7.06	10.64	14.77	**20.57**	27.82	35.72	46.29	2.33	7.83	13.35	**20.11**	27.49	34.60	43.08
30	6.21	9.43	13.18	**18.46**	25.08	32.32	42.03	1.51	6.69	11.90	**18.29**	25.25	31.97	39.98
40	5.65	8.64	12.13	**17.06**	23.26	30.05	39.18	1.62	6.46	11.30	**17.21**	23.62	29.80	37.15
50	5.24	8.06	11.35	**16.03**	21.91	28.37	37.07	2.15	6.66	11.11	**16.50**	22.33	27.92	34.54
60	4.92	7.60	10.75	**15.22**	20.85	27.05	35.40	2.94	7.09	11.14	**16.02**	21.25	26.24	32.14
70	4.67	7.23	10.25	**14.55**	19.99	25.96	34.03	3.89	7.67	11.32	**15.68**	20.32	24.73	29.91
80	4.45	6.92	9.84	**13.99**	19.25	25.05	32.87	4.96	8.35	11.60	**15.44**	19.50	23.34	27.83
84	4.37	6.81	9.69	**13.79**	18.99	24.72	32.46	5.41	8.65	11.73	**15.37**	19.20	22.81	27.03
**ΔWSS Peak_Basal [dyn/cm**^**2**^**]: Female**														
6	12.84	18.30	24.40	**32.70**	42.77	53.49	67.55	12.76	17.76	17.76	**17.76**	17.76	39.28	45.60
10	11.02	15.82	21.19	**28.53**	37.47	47.01	59.54	8.80	14.51	14.51	**14.51**	14.51	40.05	47.72
20	8.85	12.84	17.33	**23.49**	31.04	39.11	49.76	4.88	10.87	10.87	**10.87**	10.87	38.72	47.26
30	7.73	11.28	15.31	**20.85**	27.65	34.95	44.58	3.44	9.22	9.22	**9.22**	9.22	36.32	44.68
40	6.99	10.27	13.98	**19.11**	25.41	32.19	41.15	2.84	8.29	8.29	**8.29**	8.29	33.75	41.59
50	6.46	9.52	13.01	**17.83**	23.76	30.15	38.61	2.65	7.71	7.71	**7.71**	7.71	31.20	38.41
60	6.04	8.94	12.25	**16.82**	22.47	28.56	36.62	2.69	7.35	7.35	**7.35**	7.35	28.75	35.27
70	5.71	8.47	11.63	**16.01**	21.42	27.26	35.00	2.86	7.12	7.12	**7.12**	7.12	26.39	32.22
80	5.43	8.08	11.11	**15.32**	20.53	26.16	33.63	3.13	6.99	6.99	**6.99**	6.99	24.15	29.30
84	5.33	7.94	10.93	**15.08**	20.22	25.77	33.14	3.26	6.95	6.95	**6.95**	6.95	23.28	28.16
**ΔWSS Peak_Basal [dyn/cm**^**2**^**]: Male**														
6	9.41	13.80	18.79	**25.72**	34.26	43.48	55.73	9.70	15.05	20.22	**26.38**	32.96	39.20	46.54
10	8.11	12.01	16.48	**22.70**	30.42	38.77	49.89	5.23	10.67	16.03	**22.53**	29.55	36.27	44.24
20	6.56	9.86	13.68	**19.03**	25.70	32.96	42.68	1.83	6.96	12.11	**18.42**	25.30	31.94	39.85
30	5.76	8.74	12.20	**17.08**	23.19	29.87	38.81	1.34	6.10	10.86	**16.70**	23.05	29.17	36.45
40	5.23	8.00	11.23	**15.79**	21.52	27.80	36.23	1.77	6.16	10.52	**15.82**	21.55	27.06	33.60
50	4.85	7.46	10.51	**14.84**	20.29	26.26	34.31	2.64	6.67	10.61	**15.36**	20.47	25.35	31.11
60	4.55	7.03	9.95	**14.09**	19.31	25.05	32.79	3.77	7.41	10.94	**15.14**	19.63	23.88	28.89
70	4.31	6.69	9.49	**13.48**	18.52	24.06	31.55	5.07	8.32	11.42	**15.08**	18.95	22.60	26.87
80	4.10	6.40	9.10	**12.96**	17.85	23.23	30.50	6.50	9.33	12.00	**15.12**	18.39	21.46	25.03
84	4.03	6.29	8.96	**12.78**	17.61	22.93	30.12	7.10	9.75	12.25	**15.16**	18.20	21.03	24.33
**ΔRI Peak_Basal: All**														
6	-0.40	-0.35	-0.31	**-0.25**	-0.20	-0.16	-0.11	-0.44	-0.37	-0.31	**-0.25**	-0.19	-0.13	-0.07
10	-0.41	-0.36	-0.31	**-0.25**	-0.20	-0.15	-0.10	-0.44	-0.37	-0.31	**-0.25**	-0.18	-0.13	-0.06
20	-0.43	-0.36	-0.31	**-0.25**	-0.19	-0.14	-0.08	-0.44	-0.37	-0.31	**-0.24**	-0.18	-0.12	-0.06
30	-0.43	-0.37	-0.31	**-0.25**	-0.18	-0.13	-0.07	-0.44	-0.37	-0.31	**-0.24**	-0.18	-0.12	-0.05
40	-0.44	-0.37	-0.31	**-0.25**	-0.18	-0.12	-0.06	-0.45	-0.37	-0.31	**-0.24**	-0.17	-0.11	-0.05
50	-0.44	-0.37	-0.31	**-0.24**	-0.18	-0.12	-0.05	-0.45	-0.37	-0.31	**-0.24**	-0.17	-0.11	-0.05
60	-0.45	-0.37	-0.31	**-0.24**	-0.18	-0.12	-0.05	-0.45	-0.37	-0.31	**-0.24**	-0.17	-0.11	-0.04
70	-0.45	-0.38	-0.31	**-0.24**	-0.17	-0.11	-0.04	-0.45	-0.37	-0.31	**-0.24**	-0.17	-0.11	-0.04
80	-0.45	-0.38	-0.31	**-0.24**	-0.17	-0.11	-0.04	-0.45	-0.38	-0.31	**-0.24**	-0.17	-0.11	-0.04
84	-0.45	-0.38	-0.31	**-0.24**	-0.17	-0.11	-0.04	-0.45	-0.38	-0.31	**-0.24**	-0.17	-0.11	-0.04
**ΔRI Peak_Basal: Female**														
6	-0.40	-0.37	-0.33	**-0.29**	-0.25	-0.21	-0.16	-0.44	-0.38	-0.32	**-0.26**	-0.19	-0.13	-0.05
10	-0.40	-0.36	-0.33	**-0.28**	-0.23	-0.19	-0.14	-0.44	-0.38	-0.33	**-0.26**	-0.20	-0.14	-0.07
20	-0.40	-0.36	-0.32	**-0.27**	-0.21	-0.16	-0.09	-0.43	-0.38	-0.33	**-0.26**	-0.20	-0.14	-0.07
30	-0.40	-0.36	-0.31	**-0.26**	-0.20	-0.14	-0.07	-0.43	-0.37	-0.32	**-0.26**	-0.19	-0.13	-0.06
40	-0.40	-0.36	-0.31	**-0.25**	-0.19	-0.12	-0.05	-0.43	-0.37	-0.32	**-0.25**	-0.19	-0.12	-0.05
50	-0.40	-0.35	-0.31	**-0.24**	-0.18	-0.11	-0.04	-0.42	-0.36	-0.31	**-0.24**	-0.18	-0.11	-0.04
60	-0.40	-0.35	-0.30	**-0.24**	-0.17	-0.10	-0.03	-0.42	-0.36	-0.30	**-0.24**	-0.17	-0.10	-0.03
70	-0.40	-0.35	-0.30	**-0.24**	-0.17	-0.10	-0.02	-0.42	-0.35	-0.30	**-0.23**	-0.16	-0.09	-0.02
80	-0.40	-0.35	-0.30	**-0.23**	-0.16	-0.09	-0.01	-0.41	-0.35	-0.29	**-0.22**	-0.15	-0.08	0.00
84	-0.40	-0.35	-0.30	**-0.23**	-0.16	-0.09	0.00	-0.41	-0.35	-0.29	**-0.22**	-0.14	-0.08	0.00
**ΔRI Peak_Basal: Male**														
6	-0.39	-0.33	-0.29	**-0.24**	-0.19	-0.14	-0.09	-0.42	-0.36	-0.31	**-0.26**	-0.20	-0.16	-0.11
10	-0.40	-0.35	-0.29	**-0.24**	-0.18	-0.14	-0.08	-0.43	-0.36	-0.30	**-0.24**	-0.18	-0.13	-0.08
20	-0.43	-0.36	-0.30	**-0.24**	-0.18	-0.13	-0.07	-0.44	-0.36	-0.30	**-0.23**	-0.16	-0.11	-0.05
30	-0.44	-0.37	-0.31	**-0.25**	-0.18	-0.13	-0.06	-0.45	-0.37	-0.30	**-0.23**	-0.16	-0.10	-0.04
40	-0.45	-0.38	-0.31	**-0.25**	-0.18	-0.12	-0.06	-0.46	-0.38	-0.31	**-0.24**	-0.17	-0.11	-0.04
50	-0.46	-0.38	-0.32	**-0.25**	-0.18	-0.12	-0.05	-0.47	-0.38	-0.31	**-0.24**	-0.17	-0.11	-0.05
60	-0.46	-0.39	-0.32	**-0.25**	-0.18	-0.12	-0.05	-0.47	-0.39	-0.32	**-0.25**	-0.18	-0.12	-0.05
70	-0.47	-0.39	-0.32	**-0.25**	-0.18	-0.12	-0.05	-0.48	-0.40	-0.33	**-0.26**	-0.19	-0.13	-0.06
80	-0.47	-0.39	-0.33	**-0.25**	-0.18	-0.12	-0.05	-0.49	-0.40	-0.34	**-0.26**	-0.20	-0.14	-0.07
82	-0.48	-0.39	-0.33	**-0.25**	-0.18	-0.12	-0.05	-0.49	-0.41	-0.34	**-0.27**	-0.20	-0.14	-0.07
**ΔRI% Peak_Basal [%]: All**														
6	-39.37	-34.93	-30.85	**-26.21**	-21.48	-17.15	-12.25	-40.68	-35.62	-30.91	**-25.50**	-19.93	-14.81	-8.97
10	-40.23	-35.45	-31.05	**-26.03**	-20.91	-16.22	-10.90	-41.81	-36.43	-31.40	**-25.59**	-19.61	-14.10	-7.79
20	-41.38	-36.16	-31.32	**-25.79**	-20.13	-14.95	-9.06	-42.94	-37.21	-31.82	**-25.60**	-19.16	-13.22	-6.42
30	-42.04	-36.57	-31.48	**-25.65**	-19.67	-14.20	-7.98	-43.33	-37.45	-31.92	**-25.51**	-18.89	-12.77	-5.76
40	-42.51	-36.86	-31.59	**-25.55**	-19.35	-13.67	-7.21	-43.45	-37.50	-31.90	**-25.41**	-18.69	-12.49	-5.38
50	-42.88	-37.08	-31.67	**-25.47**	-19.10	-13.26	-6.61	-43.44	-37.46	-31.83	**-25.30**	-18.54	-12.30	-5.15
60	-43.17	-37.27	-31.75	**-25.40**	-18.89	-12.92	-6.12	-43.37	-37.37	-31.73	**-25.18**	-18.41	-12.16	-5.00
70	-43.42	-37.42	-31.80	**-25.35**	-18.72	-12.63	-5.71	-43.24	-37.25	-31.61	**-25.07**	-18.30	-12.05	-4.90
80	-43.64	-37.55	-31.86	**-25.30**	-18.57	-12.39	-5.35	-43.09	-37.11	-31.48	**-24.96**	-18.21	-11.98	-4.84
84	-43.72	-37.60	-31.87	**-25.28**	-18.51	-12.30	-5.22	-43.03	-37.05	-31.43	**-24.91**	-18.17	-11.95	-4.83
**RI% Peak_Basal [%]: Female**														
6	--	--	--	--	--	--	--	-41.30	-36.69	-31.99	**-26.22**	-19.93	-13.88	-6.70
10	--	--	--	--	--	--	--	-41.76	-37.23	-32.61	**-26.91**	-20.68	-14.69	-7.57
20	--	--	--	--	--	--	--	-42.01	-37.48	-32.84	**-27.10**	-20.83	-14.79	-7.61
30	--	--	--	--	--	--	--	-41.90	-37.29	-32.55	**-26.70**	-20.31	-14.15	-6.83
40	--	--	--	--	--	--	--	-41.68	-36.95	-32.11	**-26.13**	-19.59	-13.30	-5.81
50	--	--	--	--	--	--	--	-41.42	-36.57	-31.60	**-25.48**	-18.80	-12.36	-4.71
60	--	--	--	--	--	--	--	-41.12	-36.15	-31.07	**-24.81**	-17.98	-11.40	-3.58
70	--	--	--	--	--	--	--	-40.82	-35.72	-30.52	**-24.12**	-17.15	-10.43	-2.45
80	--	--	--	--	--	--	--	-40.50	-35.29	-29.97	**-23.43**	-16.31	-9.46	-1.32
84	--	--	--	--	--	--	--	-40.38	-35.11	-29.75	**-23.16**	-15.98	-9.07	-0.87
**ΔRI% Peak_Basal [%]: Male**														
6	--	--	--	--	--	--	--	-39.08	-34.75	-30.84	**-26.45**	-22.02	-18.00	-13.49
10	--	--	--	--	--	--	--	-40.62	-35.35	-30.56	**-25.18**	-19.74	-14.80	-9.25
20	--	--	--	--	--	--	--	-42.58	-36.37	-30.71	**-24.33**	-17.87	-12.01	-5.40
30	--	--	--	--	--	--	--	-43.63	-37.12	-31.17	**-24.45**	-17.65	-11.47	-4.51
40	--	--	--	--	--	--	--	-44.33	-37.73	-31.70	**-24.88**	-17.97	-11.70	-4.63
50	--	--	--	--	--	--	--	-44.83	-38.26	-32.25	**-25.44**	-18.55	-12.29	-5.23
60	--	--	--	--	--	--	--	-45.23	-38.73	-32.79	**-26.07**	-19.26	-13.07	-6.09
70	--	--	--	--	--	--	--	-45.54	-39.16	-33.33	**-26.73**	-20.04	-13.96	-7.10
80	--	--	--	--	--	--	--	-45.80	-39.56	-33.86	**-27.40**	-20.85	-14.91	-8.20
82	--	--	--	--	--	--	--	-45.84	-39.64	-33.96	**-27.53**	-21.02	-15.10	-8.43

All: female and male.

## Discussion

### Main findings

The main findings can be summarized as follows:

***First, methods used to assess macro, macro/micro and micro VR showed little association with each other. Furthermore, significant associations were mostly very weak or weak and in no case the association was very strong (r ≥0.80). The adjustment for stimulus did not result in an increase in the association between VR indexes [Table 5, Figs 3–7]***.

In our knowledge, Dhindsa et al. (2008) [4], was the first study to comprehensively assess the relationship between simultaneously measured peripheral VR indexes. In forty apparently healthy subjects (28 men; age: 19–68 y), the authors evaluated and compared different techniques and indexes used to assess VR. Schematically, ´macro´ (i.e., FMD%, change in brachial-to-radial pulse wave velocity), ´macro/micro´ (hyperemic WSS) and ´micro´ (reactive hyperemic flow, skin reactive hyperemia, fingertip pulse wave amplitude, fingertip temperature) VR indexes were quantified. The authors reported that VR indexes were not strongly associated. Furthermore, for most of the techniques assessed (75%), correlations between VR indexes did not reach statistical significance [4]. In agreement with the aforementioned, in this work VR indexes showed little association with each other. The above would mean that vascular response to a given stimulus would be a complex, composite issue, involving different and independent changes at different vascular levels and components, which would be reflected by specific indexes. These would be indicators (measure) of particular functional arterial properties and would not necessarily be affected in a similar and/or simultaneous way. As discussed by Dhindsa et al. the findings suggest that different physiological working mechanisms may be involved in the stimulation and development of vascular responses (e.g., dissimilar contribution of macro- and microvascular properties to VR indexes) [4,5].

In addition to differences in the association among them, VR indexes would show differences in their association with CRFs and/or CVD [53,54]. Related with this, previously we found that conditions like hypertensive states during pregnancy [32] and childhood obesity [3], could have a different impact on macro-, macro/micro and micro VR indexes. For instance, microvascular response was preserved whereas macrovascular response was impaired [3]. In turn, it should be noted that the impaired response was not a universal finding among the different macro/micro VR indexes (e.g., there were changes in FMD% but not in L-FMC%) [32]. Recently, Jekell et al. found weak agreement between changes in micro and macro VR in hypertensive subjects (n = 71) [53]. In Framingham Offspring, Third Generation and Omni Cohorts (n = 7031; age: 19–88 y; 54% women), macro and micro VR indexes showed differences in the association with CRFs and showed almost no correlation with each other [54]. In this regard, it has been proposed that micro-vascular dysfunction would be a prognostic biomarker whereas macro-vascular dysfunction would reflect atherosclerosis presence. Thus, the simultaneous assessment of macro and micro VR would be of value [55]. Differences in endothelial characteristics (e.g., structure, function, response to stimulus) between micro and macrovascular territories could contribute to explain the described findings [56].

The finding that micro and macro VR would show a weak (if any) correlation with each other and that they would be differently affected by different factors and/or clinical conditions should advise against extrapolating findings from one to the other [54]. In turn, the need for specific RIs for the different indexes is highlighted (see below).

***Second, RIs data showed that healthy subjects non-exposed to factors related to increased CV risk could have negative FMD% and positive LFMC% levels [Table 5]***.

**Fig 10:**
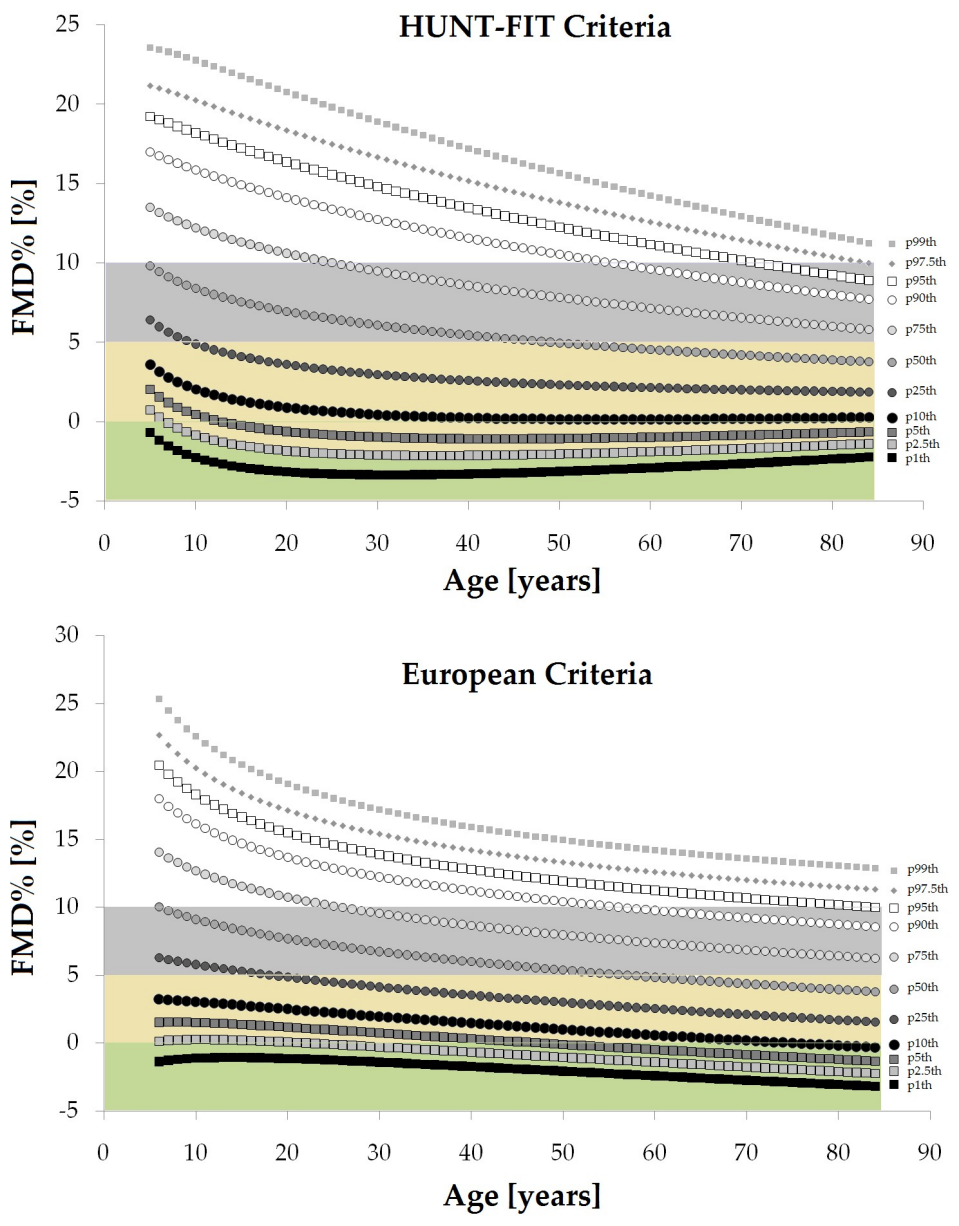
Comparative age-related profiles for FMD% considering different cut-off values to define ´low FMD%´ (based on previous reports). (i) ≤0.0% [13], (ii) ≤5.0% [57–59] and (iii)≤10% [60].

**Fig 11:**
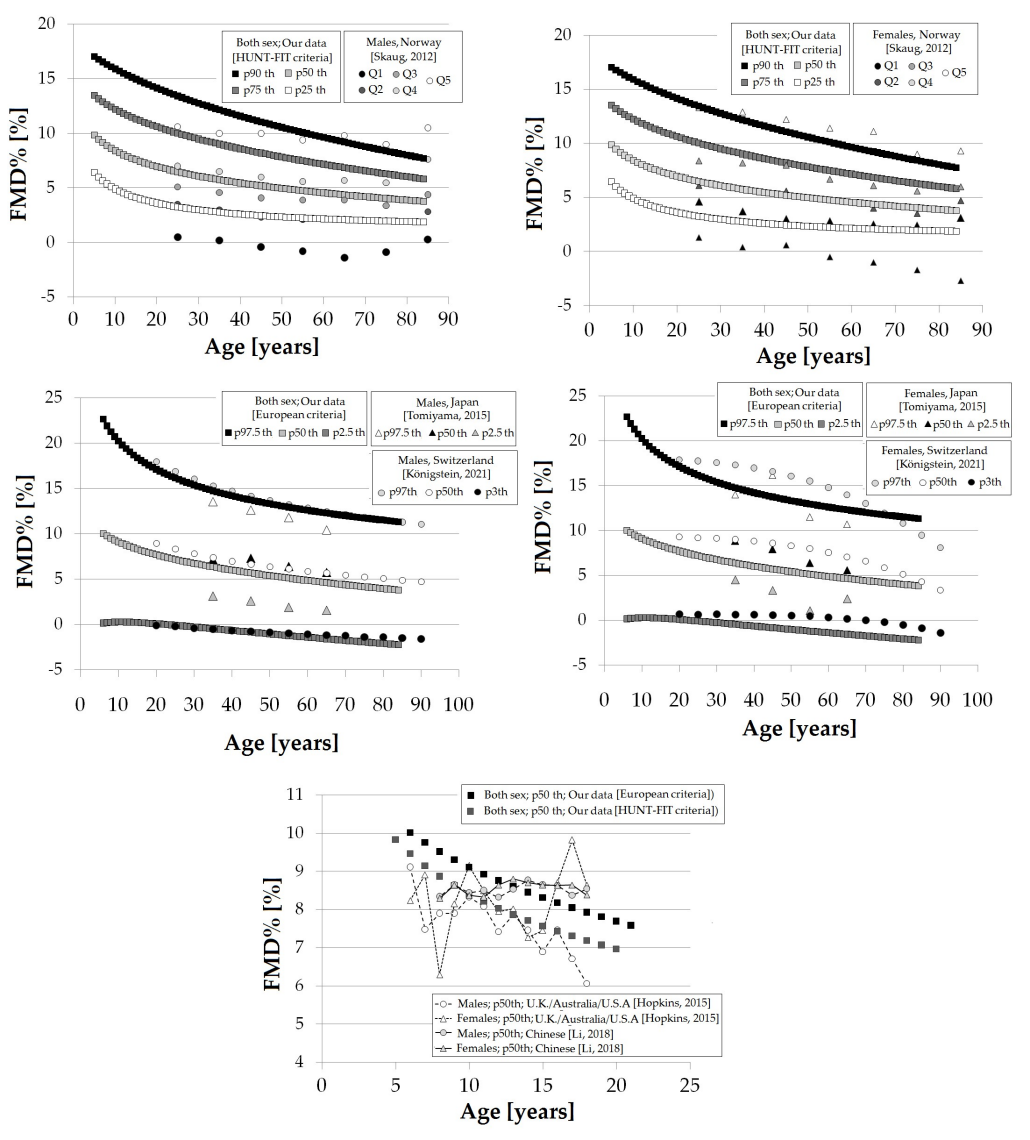
**Comparative age-related FMD% profiles for adults (top, middle) and for children and adolescents (bottom).** U.K.: United Kingdom. U.S.A.: United States of America.

In agreement with Skaug et al. [13] and Königstein et al. [15], who reported RIs for Norwegian and Swiss adults, respectively, in this work and disregard of RIs-subgroup considered (´European´ and ´HUNT-FIT´ criteria) there were subjects whose FMD% reached negative values [Table 6, Figs 10 and 11].

Therefore, in healthy subjects it would be expected to find an absence of dilatatory response during VR test, though the amount would be relatively small (e.g., below the 2.5th percentile). According to Skaug et al. [13] subjects without significant dilatation should be considered to have ´endothelial dysfunction´, even though it would not be possible to identify the explanatory factor(s) for the lack of response (e.g., failure to produce and release nitric oxide, altered smooth muscle response and/or the artery is at the structural limit of dilation) [2,14]. Looking at available data (including our results), eliminating subjects without a dilatory response at the time of constructing RIs would be an ´arbitrary definition´, which could have an effect on the RIs obtained.

In theory, a BP drop could be responsible for a negative or reduced FMD%. RH results from metabolic products accumulated during transient ischemia that determine microcirculatory vasodilation. The resulting fall in microvascular resistance leads to increased blood flow upstream in BA, so that its own resistance to flow may become significant (at least until its own vasodilation occurs) and create a BP loss, which may cause passive BP-dependent mechanical constriction (elastic recoil) [61]. Higher positive FMD% values were observed in association with greater RH and/or smaller BA pressure drop [61]. In this respect, dilatation in the VR test would be ´masked´ by the constriction associated to BP drop. Differences in RH and/or in baseline BA distension (strain) would contribute to explain the imbalance between opposite mechanisms and differences in the observed reponses (dilatation vs. constriction).

**Fig 12:**
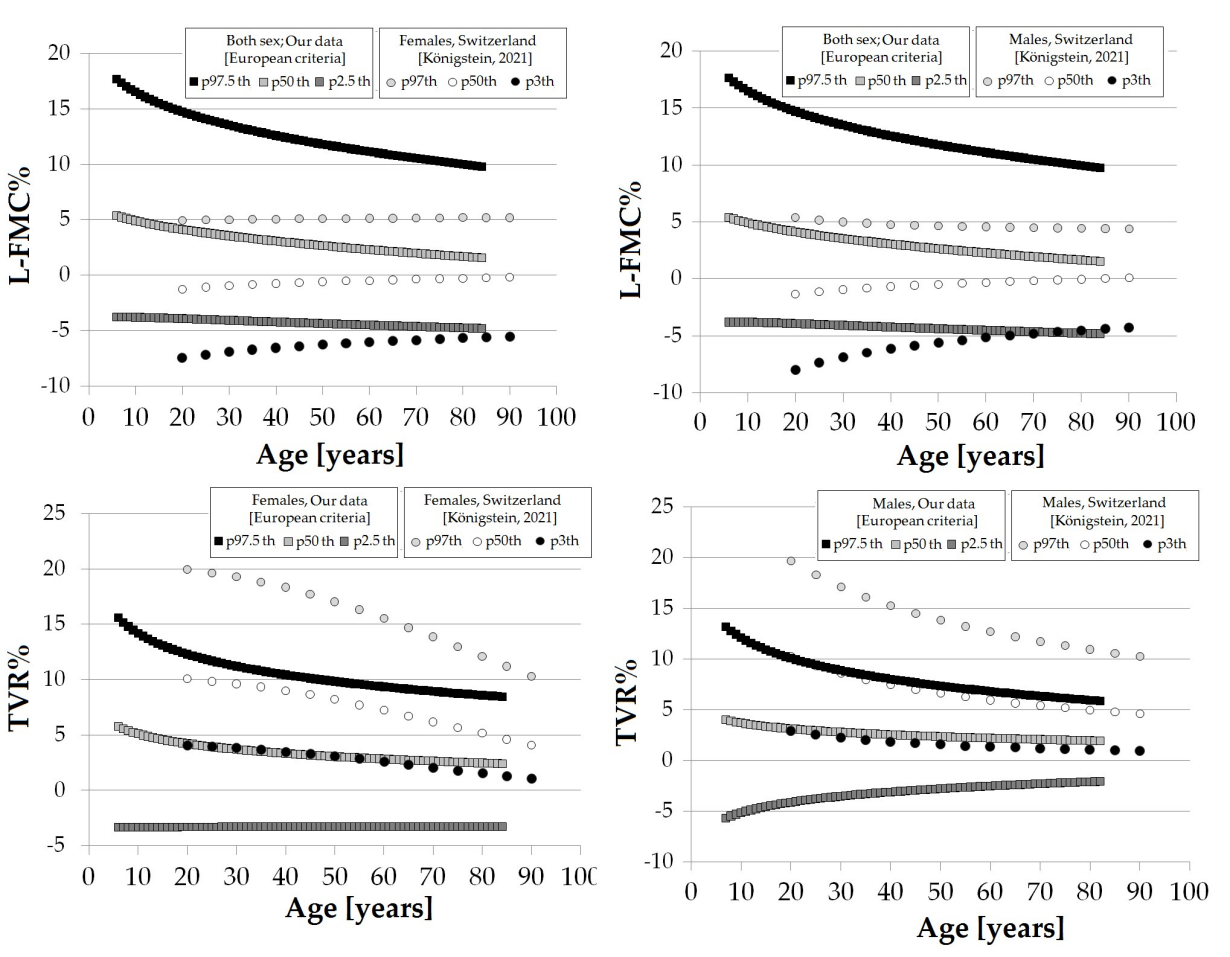
Comparative age-related L-FMC% and TVR% profiles.

In agreement with data reported by Königstein et al. data (n = 457, age: 20–91 y, 50% females, nonsmoking without chronic diseases and regular medication; very low CV risk) [15] [Fig 12], a significant number of subjects showed positive L-FMC% values (pre-release BA diameters larger than basal diameters). This was ´corrected´ (although partially) when L-FMC% was adjusted for stimulus [Table 6]. This is in agreement with Thijjsen et al., who observed that BA diameters were larger during the cuff-inflation (distal arterial occlusion) than in basal conditions [62]. The ´impact´ of distal cuff-inflation on BA diameter was age-dependent. In children, adolescents and young adults, cuff inflation was associated with an increase in BA diameter, whereas in older subjects the changes (differences) were not significant (probably due to the age-related increase in arterial stiffness) [62]. It should be noted that available data regarding BA diameter levels (and changes) associated with arterial occlusion are controversial [7,63–66]. L-FMC% in BA would not be a universal response, as observed in the radial artery. In this regard, L-FMC% was firstly described as a radial artery specific-response [38] and there is agreement in the presence of radial constriction associated with proximal cuff inflation (zero blood flow and ischemic metabolites accumulation) [38,65,67]. Changes in BA diameter during cuff inflation would not be explained by ischemic metabolites accumulation, but could be the result of BP-related factors [62]. An increase in BA diameter during cuff-occlusion could contribute to explain the finding of negative values when calculating TVR [Table 6, Fig 12].

Fig 12 shows age-related profiles for L-FMC% obtained in this and in Königstein et al. work. [15]. Positive L-FMC% values were observed in both populations, but L-FMC% levels and age-related profiles differed (similarities were mainly observed at older ages). Biological (e.g., age-related differences in arterial stiffness) as well as methodological (e.g., distance between the proximal edge of the cuff and the ultrasound probe, cuff size) factors could contribute to explain the findings and the differences between groups. As described, L-FMC% would depend on flow variations during cuff-inflation. Thus, at the time of analyzing the vascular response in a subject or population, it would be appropiate to evaluate the variations in diameter considering (knowing) the local (BA) hemodynamic changes associated with cuff-inflation.

FMD% and L-FMC% have demonstrated association with CV risk. However, as stated above, their data depend on local hemodynamic factors. Further works would be necessary: (i) to isolate VR and characterize the responsiveness to a given stimulus, (ii) to develop indexes that consider (e.g., adjust for) brachial BP acute or transient impact on vascular response and/or (iii) to integrate complementary data from different tests so as to accurately characterize VR and/or EF. In this regard, in addition to tests (and indexes) that evaluate the response to transient changes in blood flow and WSS, it would be useful to analyze the response to ´sustained´ changes (e.g. increases) in WSS (e.g., by limb heating, vasodilators infusion, exercise) that would mimic *in vivo* physiological conditions [68]. Transient and sustained WSS stimuli may test different aspects of vascular physiology. Thus, they could be complementary [68].

***Third, although macro VR indexes depended on BA diameter and BP, the size effect would be low, even more so when considering VR indexes adjusted for the hyperemic stimulus*** (e.g., ΔDD Peak-Basal/ΔVPeak-Basal, FMD/ΔVPeak_Basal, pFMDv, FMD%WSS) [Fig 8].

**Fourth**, **the need for sex-specific RIs relied on the VR index and/or age considered** *[*S7 and S8 *Tables* in S1 File*]*.

The relationship between sex and VR is controversial. The described sex-related differences in VR could not be explained by sex-related differences in arterial properties *per se*, but by sex-related differences in arterial sensitivity to CRFs and/or by the age of the studied subjects. On the other hand, it should be noted that sex-related differences were in many cases defined on the basis of statistical results (e.g., p-value), disregard of the true ´effect size´. Additionally, in some cases the definition and/or analysis of sex-related differences was done without taking into account cofactors (i.e., exposure to CRFs) [17].

In 1994, Celermajer et al. studied healthy subjects (n = 500, age: 36±15 (5–73) y; 248 females) without known CVD, hypertension, familial hypercholesterolemia and homocystinuria; none of the included subjects was taking cardioactive drugs [69]. However, subjects could be exposed to other CRFs (e.g., tobacco use, high cholesterol). After adjusting for basal diameter and CRFs, the authors found larger FMD% levels in females than in males [69]. In healthy subjects (n = 40, age: 23–52 y) non-exposed to CRFs (i.e., hypertension, diabetes, smoking, hypercholesterolemia, family history of heart disease), Corretti et al. (1995) did not find differences in FMD% between males and females <40 y of age, whereas at older ages larger FMD% values were observed in females (data not adjusted for BA basal diameter) [70]. In a group of subjects (n = 20, age: 31±3 y) without history of hypertension, hyperlipidemia, diabetes or current smoking, Uehata el al. (1997) found larger FMD% values in females than in males of similar age (14.1±6.0% vs. 5.3±2.3%, p<0.001) [71]. There were no differences in the hyperemic stimulus between males and females. It should be noted that males had lower HDL-cholesterol and higher mean brachial BP levels [71]. The described differences in FMD% were attributed to differences in BA diameter since it was the only explanatory factor for BA response in a multivariate analysis considering sex, bSBP, HDL-cholesterol, in addition to BA diameter [71].

Herrington et al. (2011) analyzed data from epidemiological and clinical trials (n = 4040, age: 74.5±13.1 y, range. 13.8–97.8 y.). The authors found larger FMD% values in females than in males (3.89±2.75 vs. 3.16±2.14%, p<0.001), which was primarily explained by the smaller basal diameters observed in females [14]. The hyperemic stimulus and absolute changes in diameter did not show sex-related differences. After adjusting for age and baseline diameter, lower FMD% values (and absolute changes in BA diameter) were observed in females (3.50±1.55 vs. 3.70±0.06%, p = 0.027). Taking into account the age of the subjects (mean: 74.5 y; most subjects between 75–85 y) and the greater vasodilatory response described in association with premenopausal status, women ≤50 y (n = 140) and men of similar age (n = 164) were compared. After adjusting for age and basal diameter, there were no significant differences in FMD% or absolute changes in arterial diameter between females and males (6.48±0.33% vs. 6.49±0.30% and 0.25±0.01 vs. 0.25±0.01mm, respectively) [14]. Unfortunately, other potential covariates (e.g., cholesterol levels, smoking status, CVD, lipid and BP-lowering drugs use) were not considered.

In 2015, Hopkins et al. published sex-specific RIs for VR indexes considering data obtained in children and adolescents from United Kingdom, United States of America and Australia (n = 978, age: 6–18 y) [18]. In turn, RIs for Chinese subjects were defined in 2018 by Li et al. (n = 1637; age: 8–18 y) [19]. Hopkins et al. stated that sex-related differences in FMD% were only apparent at 17 and 18 years old. When FMD% was adjusted for basal BA diameter, sex-related differences were attenuated (non-significant). Li et al. found sex-related differences in FMD% in 12 and 13 year old subjects [19]. Note in Fig 11 the overlapping of FMD% curves for females and males, obtained from data published by Hopkins et al. and Li et al.

Skaug et al. reported sex-specific RIs for FMD% (Norwegian subjects, n = 4739; 20–89 y, obese and smokers included) [13]. Shear rate yielded similar patterns for age and sex. The authors reported higher FMD% values in females (5.33%, 95%CI: 5.15–5.51%) than in males (4.29%, 95%CI: 4.12–4.45%) across different age-groups from 20 to 70 y (p<0.001). For subjects older than 70 y., sex-related differences were no longer statistically significant (p = 0.21) [13]. The authors analyzed (in the same group of subjects) the association between EF and exposure to CRFs [72]. They found that hyperglycemia, high BP, low fitness and a cluster of CRFs comprising the metabolic syndrome were more strongly associated with reduced FMD% in females than in males. Since RIs published by Skaug et al. were obtained in subjects exposed to some traditional CRFs, it is unknown whether the sex-related differences in VR were influenced by sex-related differences in susceptibility to CRF impact, which could not be considered by (merely) adjusting for the number of CRFs. It would be of interest to determine whether the described differences would be observed in males and females not exposed to CRFs.

Königstein et al. did not find sex-related differences in L-FMC% values (age-related differences were similar in females and males), which is in agreement with our findings [15].

***Fifth, population-based RIs for macro, macro/micro and micro VR indexes were defined from data obtained in the same group of subjects*.** Defining RIs is an important step when considering the introduction of VR indexes in research and clinical practice (e.g., to identify conditions associated with data deviation form expected values in physiological settings and/or to detect subclinical target organ damage) [Table 6; S10-S63 Tables in S1 File; S2 File]. To our knowledge this is the first time RIs for different ´macro´, ´macro/micro´ and ´micro´ VR indexes were determined (at the same time) in large groups of healthy children, adolescents and adults (3–85 y), defined taking into account inclusion and exclusion criteria used by our and other groups.

Despite several groups published FMD% data, only few analyzed them as a function of age. Fig 11 shows the RIs for FMD% defined in this work, together with profiles obtained from data published by other authors. Note the similarity between this work FMD% data for adults and those obtained by Skaug et al. [13], despite profiles for Skaug et al. data were constructed (in the Figure) from MVs for each age-decade whereas in this work RIs were developed based on year-by-year data [Fig 11]. The p50th obtained in this work and Q3 (range: p40th to p60th) determined from Skaug et al. data showed similar profiles (slopes) in males and females. This work FMD% data and those obtained by Königstein et al. for adult males almost overlapped and showed similar age-related profiles [13] [Fig 11]. A similar observation can be made when comparing the results of this work and those of Tomiyama et al. (n = 1908, females: 28%, without CRFs and CV disease; data obtained as mean values for each decade) [17] [Fig 11]. Data obtained for females by the different groups (values and profiles) did not show the similarity described for males [Fig 11]. The analysis of the explanatory factors and (practical) significance of the above is beyond the scope of this work, but in any case it highlights the importance of considering appropriate references values.

Findings were heterogeneous when data from children and adolescents (6–18 y) were analyzed. Comparing data obtained in subjects from United Kingdom, United States of America and Australia, with this work data (´European Criteria´) there were ages at which data were different and ages at which data almost overlapped and showed a similar tendency to decrease with age [18]. When comparing our data with those from Chinese children [19], there were ages in which there was an almost perfect overlapping (e.g., 10–15 y), despite the authors did not find an age-related reduction in FMD%, but reported a flattened age-related profile between 6 and 18 y [Fig 11].

As expected, FMD% values (p50th) obtained for the RIs-subgroup defined following restrictive criteria (´European´ criteria) were higher than those for the subgroup obtained in agreement with a comprehensive criteria (´HUNT-FIT´ criteria) [Fig 11].

Different cut-off values have been used to define ´low FMD%´. In this regard, based on data from receiver-operating characteristic (ROC) curve analysis some authors defined an FMD% ≤5.0% (lowest 30th percentile) as the cut-off value [57–59]. Other authors considered an FMD% ≤10.0% to define a reduced FMD response [60]. The cut-off values were (mainly) defined considering BA FMD% data obtained in control subjects and patients with coronary artery disease. According to our findings, regardless of the RIs-subgroup and life stage considered, there would be (healthy) subjects with FMD values ≤5.0%. The number of subjects fulfilling this condition would be: (i) ~10% of 6- year-olds, (ii) ~25% of 20-year-olds, (iii) ~50% of adults aged 50–55 y, and (iv) ~70% of subjects aged 80–84 y [Fig 10]. If a cut-off value equal to 10% were considered, subjects with reduced FMD would represent: (i) ~50% of 6-year-olds, (ii) ~75% of 20-year-olds, (iii) ~90% of adults aged 50–55 y, and (iv) ~95% of subjects aged 80–84 y [Fig 10]. The above advises against the universal use of particular cut-off values since it could lead to over-diagnose impaired VR and EF. Furthermore, selecting inadequate cut-off points to define normal and abnormal VR responses would impact negatively on the clinical value ascribed to the analyzed response. Further studies will contribute to determine and define the most appropriate index (or indexes) and the corresponding cut-off values that should be applied in a given subject and population.

### Strengths and limitations

Our results should be analyzed in the context of the work´s strengths and limitations. First, it should be noted that even using the previously described definitions for ´micro´, ´macro´ and ´macro/micro´ VR indexes [4], the distinction is often difficult or even inaccurate and probably all the obtained data would be the result or reflect a combination (of different relative magnitude) of both macro- and microvascular reactivity [4,5]. Second, since this is a cross-sectional study, it provides no data on longitudinal age-related variations in VR indexes. Third, outcome data were not considered. Thus, cut-off points (e.g., p75th, p90th, p95th) could not be selected based on the association with increased CV risk, but on data distribution in the RIs group. Whether or not the RIs values should be used as cut-off values for diagnose and treatment is unknown. Fourth, in this work, the concept of VR was (mainly) presented as ´static or unchanged´, rather than the composite of (i) ´fixed or stable´ (e.g., age-dependent vascular (intrinsic) capability to produce and respond to vasoactive factors) and (ii) ´variable or adjustable´ (e.g., endothelial and vascular smooth muscle capability to temporally adjust their function). The systematization of recording conditions is necessary to evaluate VR considering the existence of modulating factors. In this work, to systematize the records and as a way to minimize the impact of sources of variability, VR levels were assessed and determined at rest, under stable hemodynamic conditions. Fifth, as a strength, in this study VR indexes were obtained in a large population sample (of children, adolescents and adults) that included subjects within a wide age-range (almost the whole life expectancy range), as a continuum. This would contribute to understand VR behavior (levels and variations) throughout life.

## Conclusions

VR indexes used to assess macro, macro/micro and micro VR responses showed little association with each other. Adjusting for the stimulus did not result in an increase in the strength of association between VR indexes. Macrovascular responses were not associated (or the association was very weak) with microvascular responses or RH stimulus indexes.

Healthy subjects non-exposed to factors associated with increased CV risk could have negative FMD%.

The need for sex-specific RIs relied on the parameter and/or age considered. RIs for different macro, macro/micro and micro VR indexes were defined (at the same time) in a large population of healthy children, adolescents and adults (3–85 y). Equations for mean, standard deviation and percentiles values (sex- and/or age- specific) were included in text and spreadsheet formats. Thus, expected values for a given subject can be calculated.

## Supporting information

S1 File Table S1. Subjects demographic, anthropometric and clinical characteristics: All. Table S2. Brachial artery characteristics and vascular reactivity indexes: All. Table S3. Subjects demographic, anthropometric and clinical characteristics: Reference Intervals subgroup (´European criteria´). Table S4. Brachial artery characteristics and vascular reactivity indexes: Reference Intervals subgroup (´European criteria´). Table S5. Subjects demographic, anthropometric and clinical characteristics: Reference Intervals subgroup (´HUNT-FIT criteria´). Table S6. Brachial artery characteristics and vascular reactivity indexes: Reference Intervals subgroup (´HUNT-FIT criteria´). Table S7. Multiple regression models (with interaction terms between age and sex) as determinant of vascular reactivity indexes: Reference Intervals subgroup (´European criteria´ and ´HUNT_FIT criteria´). Table S8. Age-related and/or sex-related RIs: schematic diagram. Table S9. Age-related mean and standard deviation equations: mathematical model summary (Reference Intervals subgroup: ´European criteria´ and ´HUNT-FIT criteria´). Table S10. ΔDD Peak_Basal [mm] reference intervals: All (European criteria). Table S11. DD Ratio Peak_Basal [%] reference intervals: All (European criteria). Table S12. FMD% [%] reference intervals: All (European criteria). Table S13. TPD FMD% [seconds] reference intervals: All (European criteria). Table S14. ΔDD Peak-Basal/ΔVPeak-Basal [mm/cm/s] reference intervals: All (European criteria). Table S15. FMD/ΔVPeak_Basal [1/cm/s] reference intervals: All (European criteria). Table S16. pFMDv reference intervals: All (European criteria). Table S17. FMD% WSS reference intervals: All (European criteria). Table S18. ΔDPrerelease_Basal [mm] reference intervals: All (European criteria). Table S19. DD Ratio Prerelease_Basal [%] reference intervals: All (European criteria). Table S20. L-FMC% [%] reference intervals: All (European criteria). Table S21. ΔDDPrerelease_Basal/ΔEDVPrelease_Basal reference intervals: All (European criteria). Table S22. L-FMC/ΔVPrerelease_Basal [1/cm/s] reference intervals: All (European criteria). Table S23. pL-FMCv reference intervals: All (European criteria). Table S24. L-FMC%/WSS reference intervals: All (European criteria). Table S25. TVR [%] reference intervals: All (European criteria). Table S26. TVR [%] reference intervals: Female (European criteria). Table S27. TVR [%] reference intervals: Male (European criteria). Table S28. ΔVPeak_Basal [cm/s] reference intervals: Male (European criteria). Table S29. ΔWSSPeak_Basal [dyn/cm2] reference intervals: All (European criteria). Table S30. ΔWSSPeak_Basal [dyn/cm2] reference intervals: Female (European criteria). Table S31. ΔWSSPeak_Basal [dyn/cm2] reference intervals: Male (European criteria). Table S32. ΔRIPeak_Basal reference intervals: All (European criteria). Table S33. ΔRIPeak_Basal reference intervals: Female (European criteria). Table S34. ΔRIPeak_Basal reference intervals: Male (European criteria). Table S35. ΔRI%Peak_Basal [%] reference intervals: Male (European criteria). Table S36. ΔDD Peak_Basal [mm] reference intervals: All (HUNT-FIT criteria). Table S37. DD Ratio Peak_Basal [%] reference intervals: All (HUNT-FIT criteria). Table S38. FMD% [%] reference intervals: All (HUNT-FIT criteria). Table S39. TPD FMD% [seconds] reference intervals: All (HUNT-FIT criteria). Table S40. ΔDD Peak-Basal/ΔVPeak-Basal [mm/cm/s] reference intervals: All (HUNT-FIT criteria). Table S41. FMD/ΔVPeak_Basal [1/cm/s] reference intervals: All (HUNT-FIT criteria). Table S42. pFMDv reference intervals: All (HUNT-FIT criteria). Table S43. FMD%WSS reference intervals: All (HUNT-FIT criteria). Table S44. ΔDPrerelease_Basal [mm] reference intervals: All (HUNT-FIT criteria). Table S45. DD Ratio Prerelease_Basal [%] reference intervals: All (HUNT-FIT criteria). Table S46. L-FMC% [%] reference intervals: All (HUNT-FIT criteria). Table S47. ΔDDPrerelease_Basal/ΔEDVPrelease_Basal reference intervals: All (HUNT-FIT criteria). Table S48. L-FMC/ΔVPrerelease_Basal [1/cm/s] reference intervals: All (HUNT-FIT criteria). Table S49. pL-FMCv reference intervals: All (HUNT-FIT criteria). Table S50. L-FMC%/WSS reference intervals: All (HUNT-FIT criteria). Table S51. TVR [%] reference intervals: All (HUNT-FIT criteria). Table S52. TVR [%] reference intervals: Female (HUNT-FIT criteria). Table S53. TVR [%] reference intervals: Male (HUNT-FIT criteria). Table S54. ΔVPeak_Basal [cm/s] reference intervals: All (HUNT-FIT criteria). Table S55. ΔWSSPeak_Basal [dyn/cm2] reference intervals: All (HUNT-FIT criteria). Table S56. ΔWSSPeak_Basal [dyn/cm2] reference intervals: Female (HUNT-FIT criteria). Table S57. ΔWSSPeak_Basal [dyn/cm2] reference intervals: Male (HUNT-FIT criteria). Table S58. ΔRIPeak_Basal reference intervals: All (HUNT-FIT criteria). Table S59. ΔRIPeak_Basal reference intervals: Female (HUNT-FIT criteria). Table S60. ΔRIPeak_Basal reference intervals: Male (HUNT-FIT criteria). Table S61. ΔRI%Peak_Basal [%] reference intervals: All (HUNT-FIT criteria). Table S62. ΔRI%Peak_Basal [%] reference intervals: Female. (HUNT-FIT criteria). Table S63. ΔRI%Peak_Basal [%] reference intervals: Male (HUNT-FIT criteria).
(XLSX)

S2 File Figure S1. Age-related profiles for vascular reactivity indexes: ´European criteria. Figure S2. Age-related profiles for vascular reactivity indexes: ´HUNT.FIT criteria.
(DOCX)
