# Supplementary material for: Age- and sex-related profiles for macro, macro/micro and microvascular reactivity indexes: Association between indexes and normative data from 2609 healthy subjects (3-85 years)
Source: PLoS One. 2021 Jul 19;16(7):e0254869. doi: 10.1371/journal.pone.0254869 (PMC8289111; doi:10.1371/journal.pone.0254869)

**Figure S1. Age-related profiles for vascular reactivity indexes: ´European criteria´**


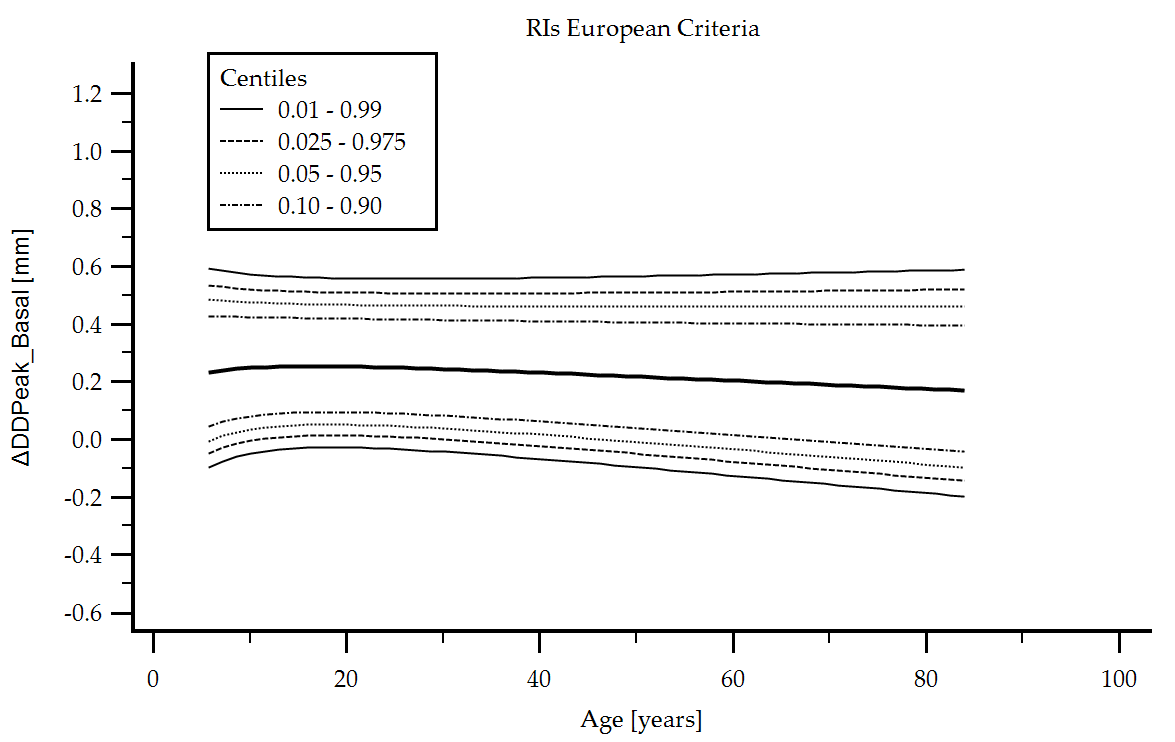


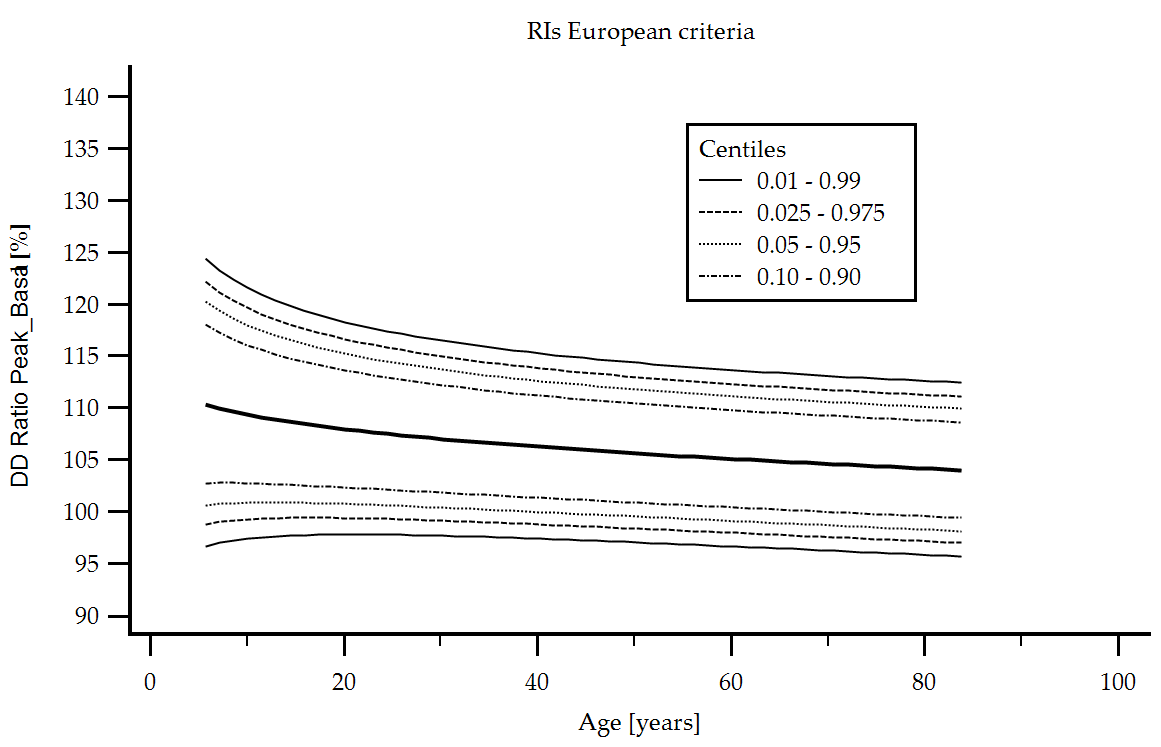

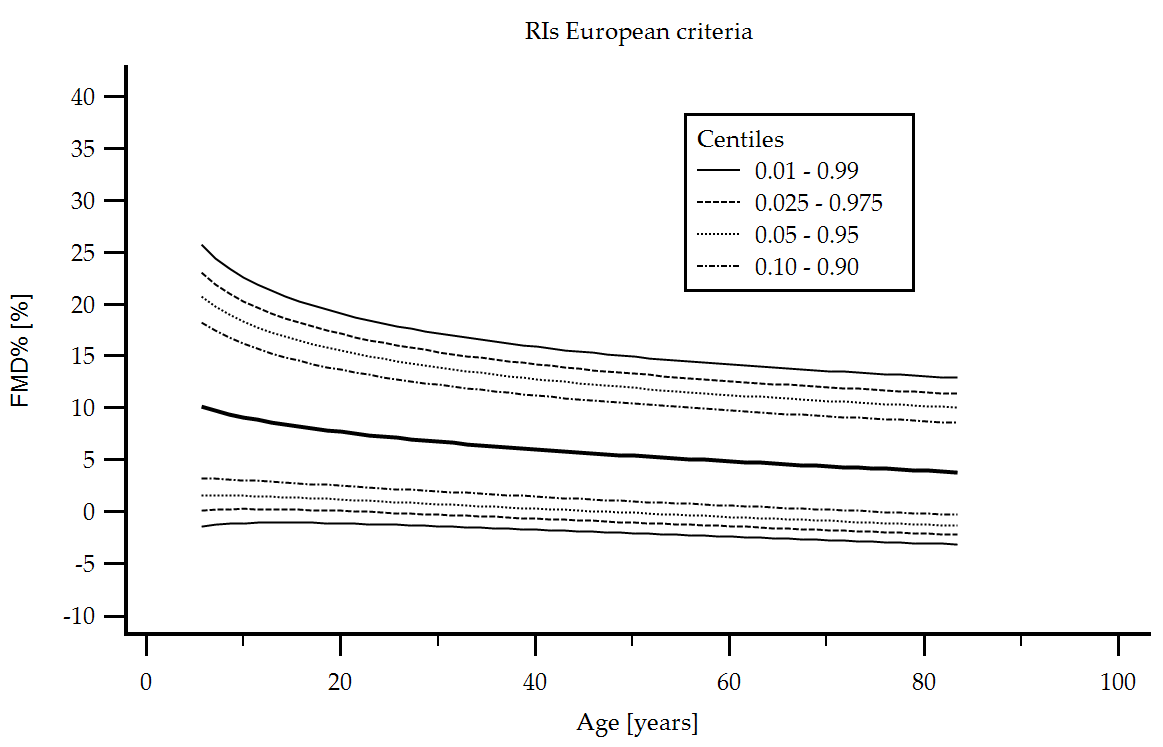


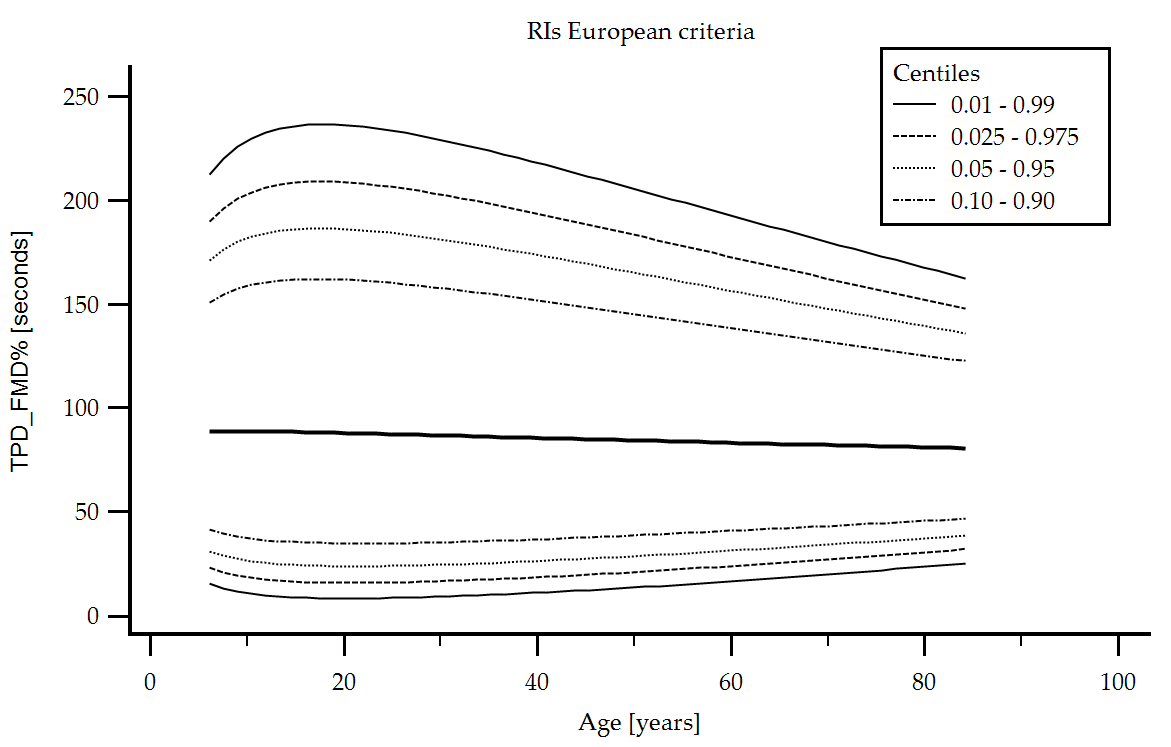


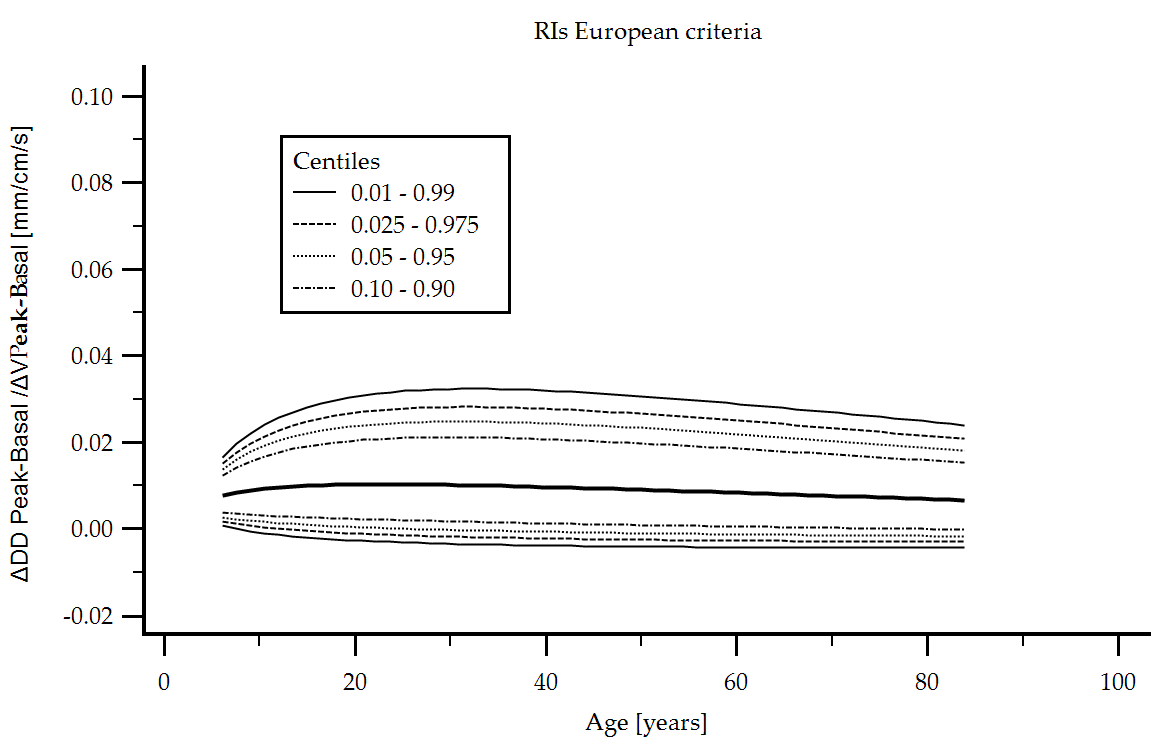


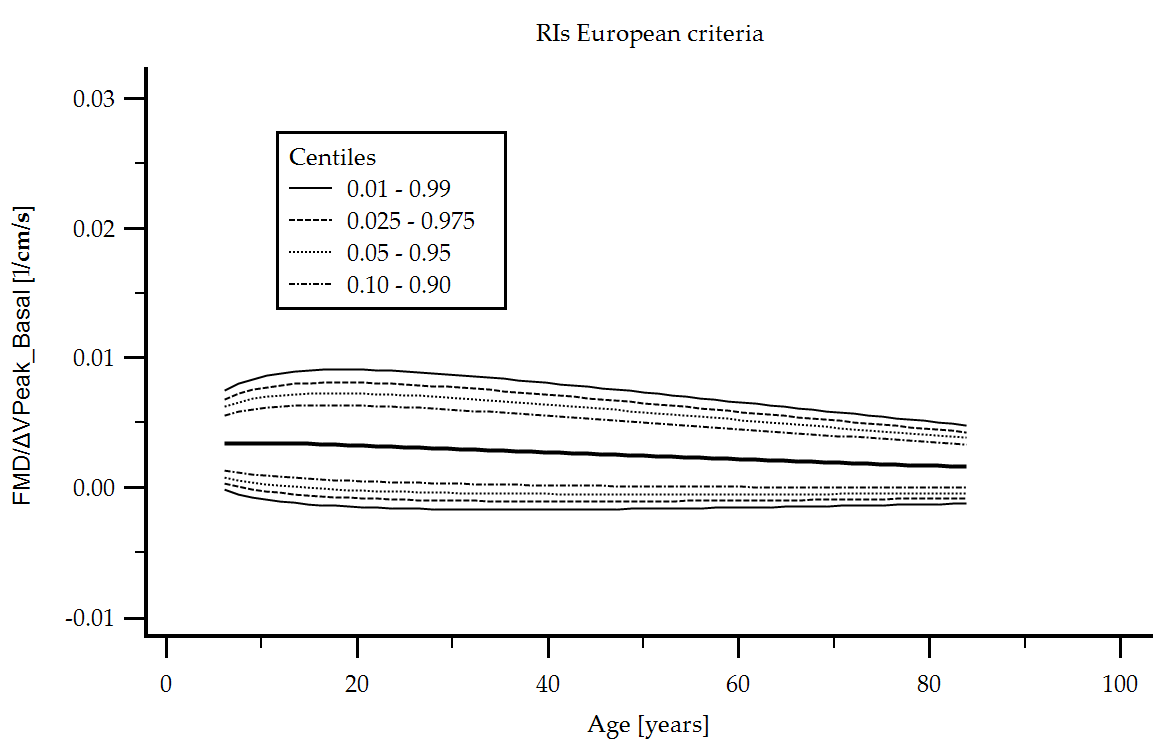


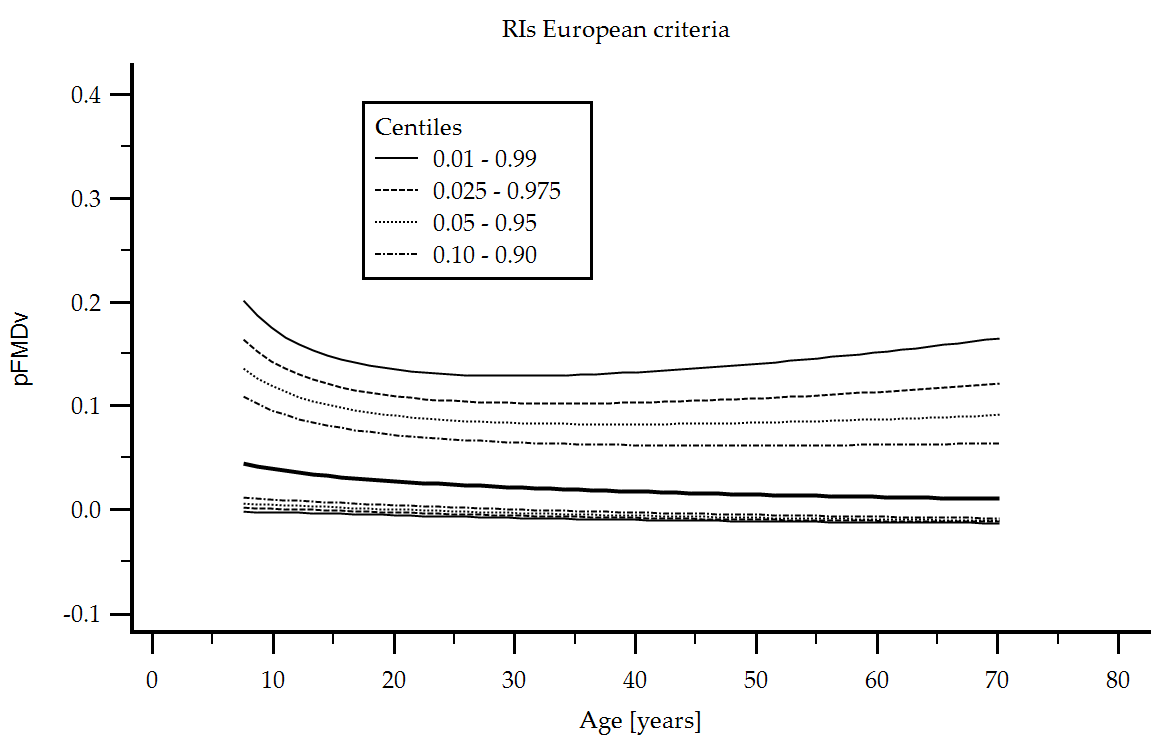


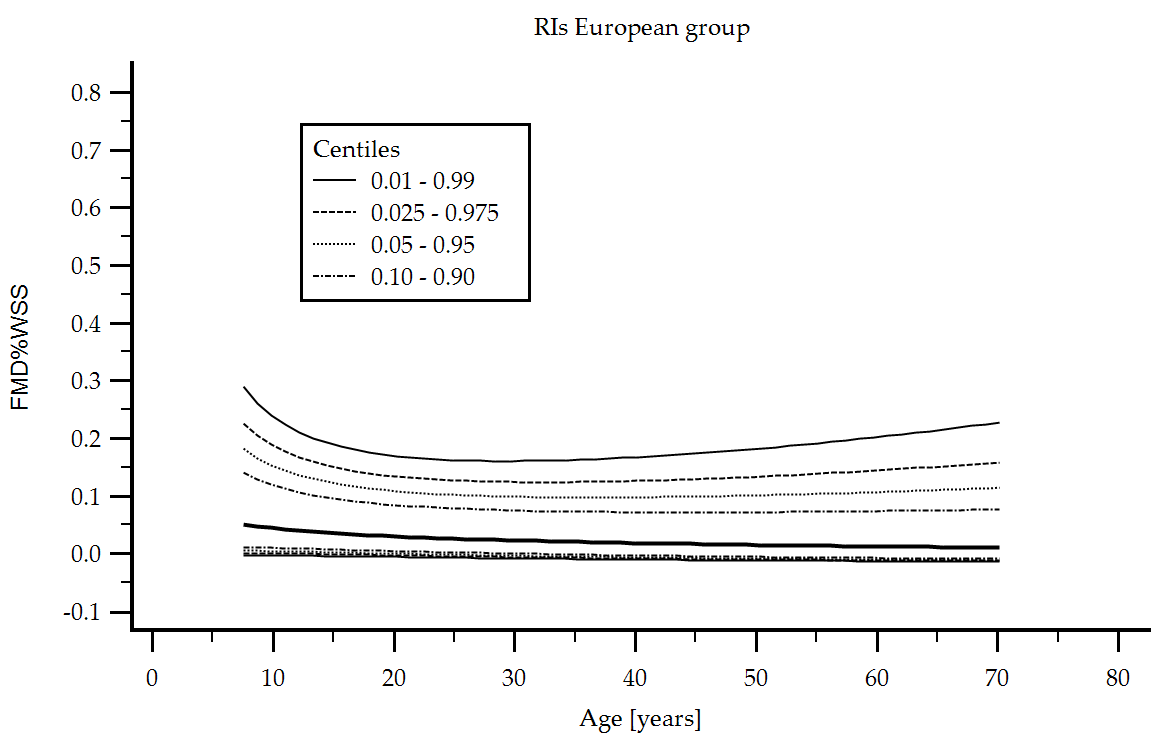


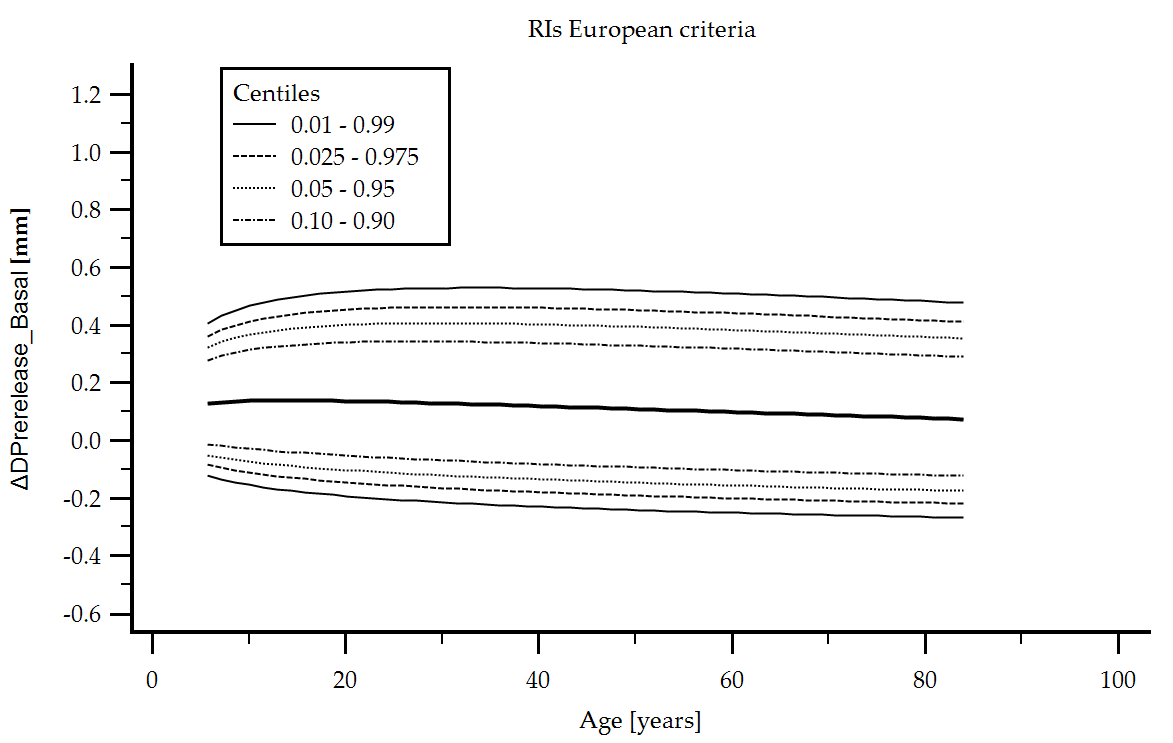


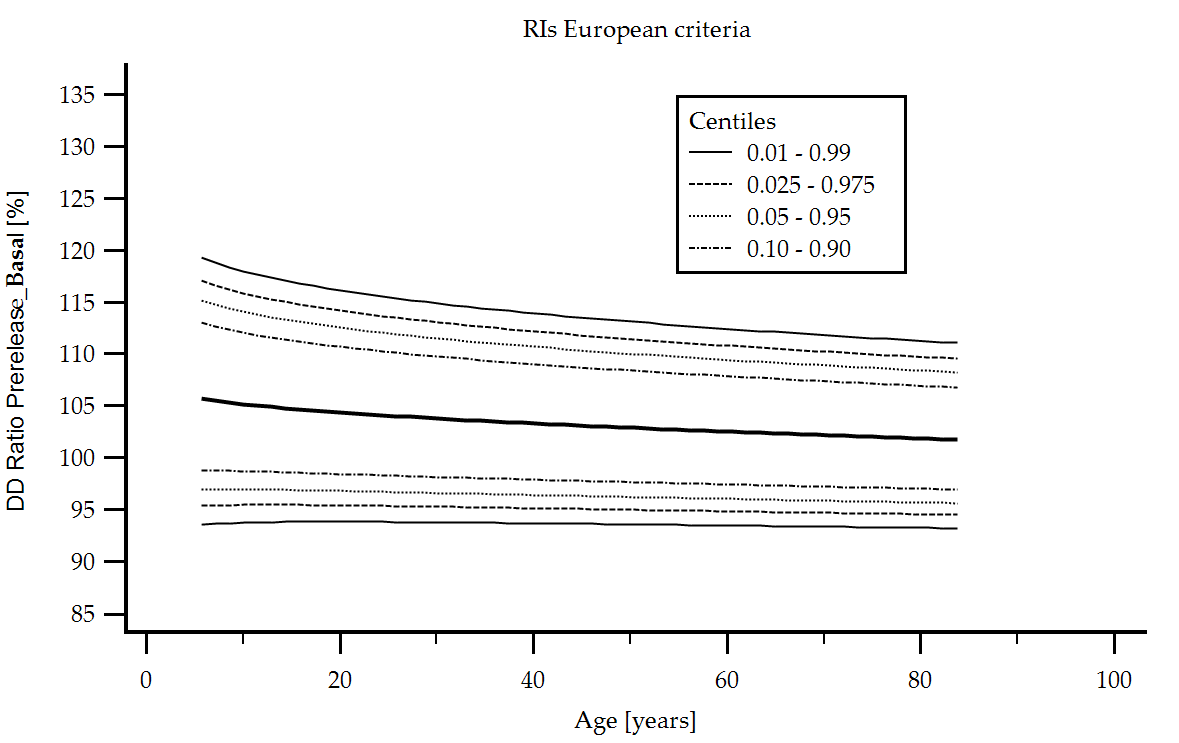


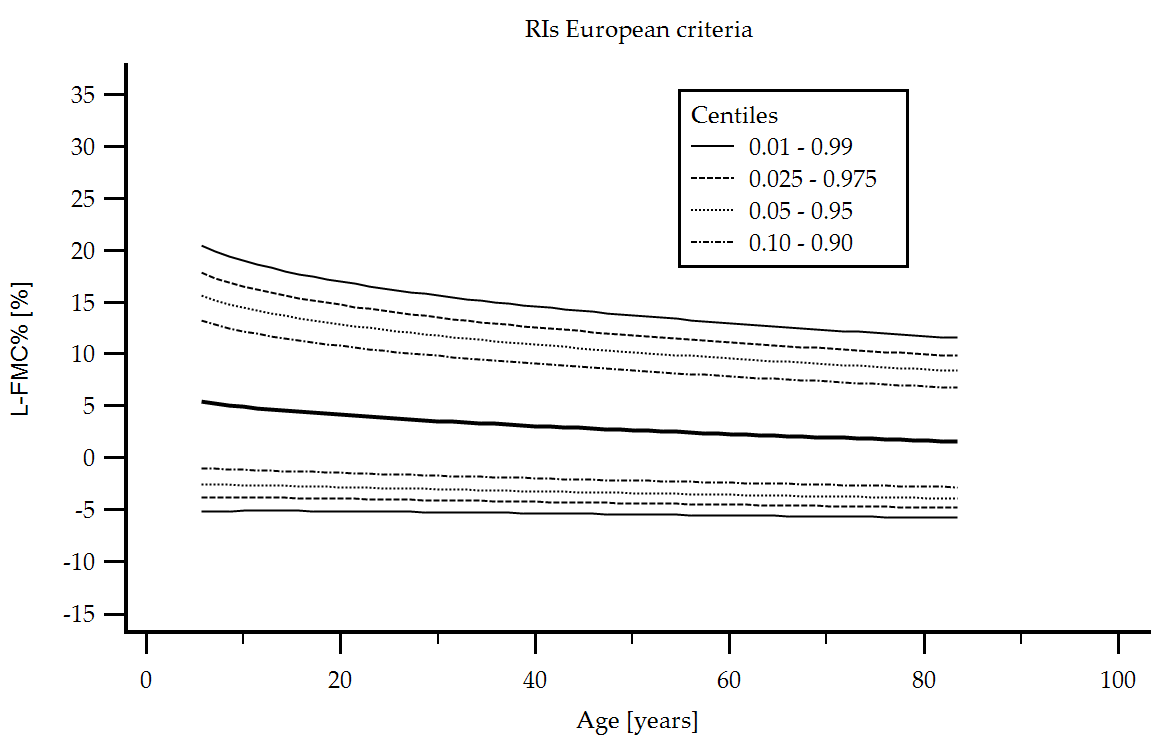


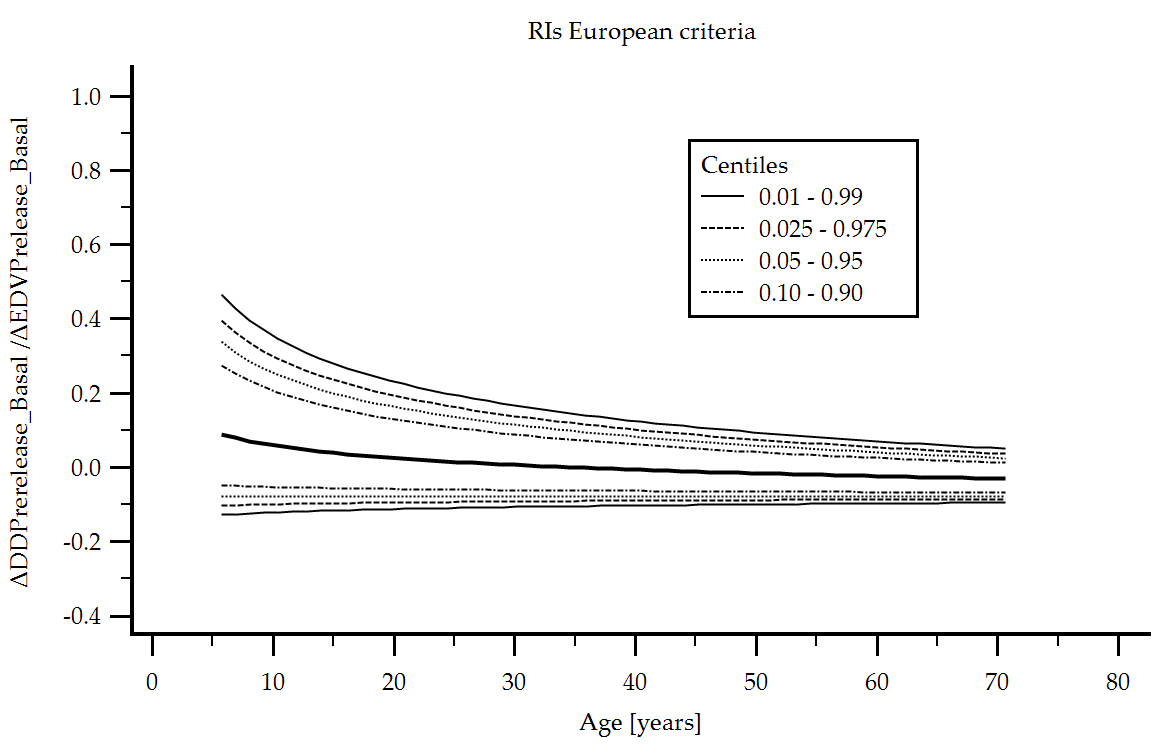


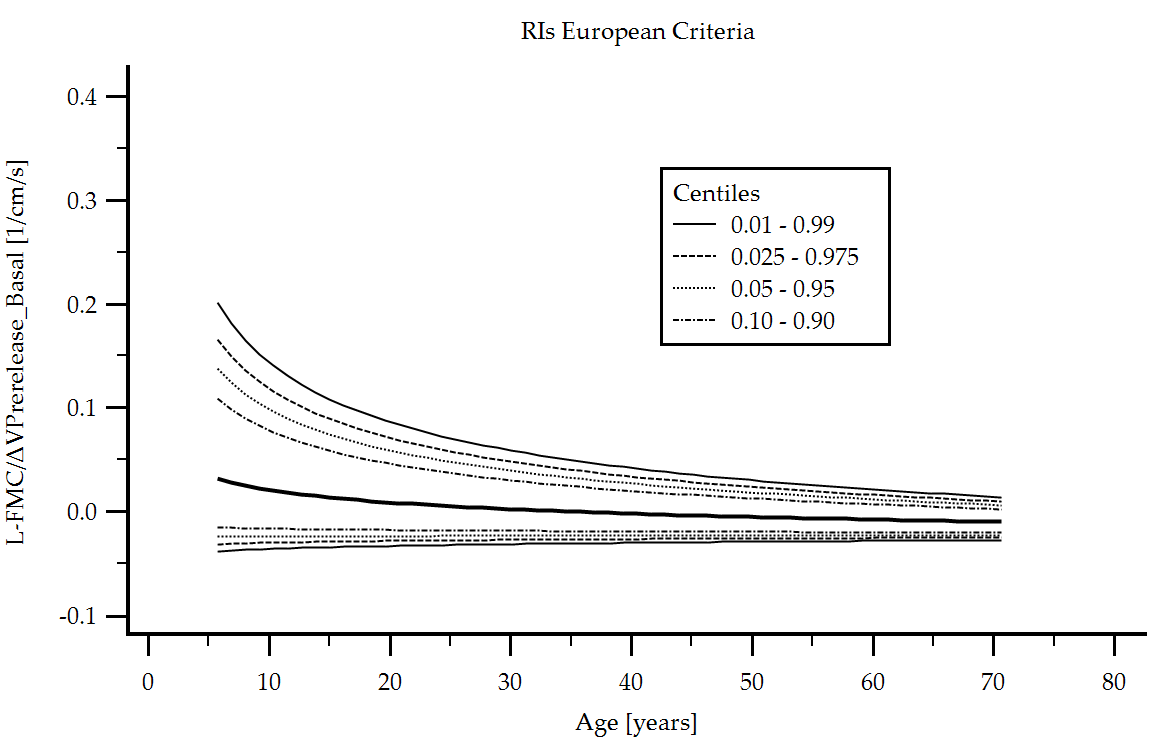


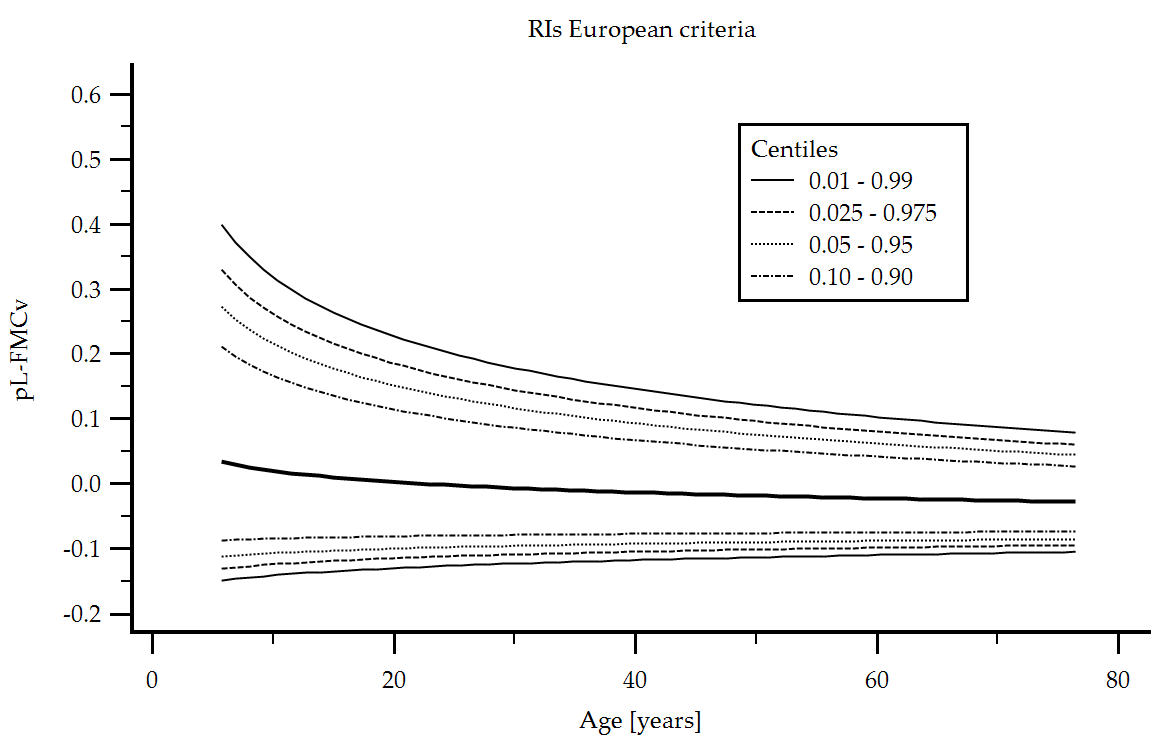


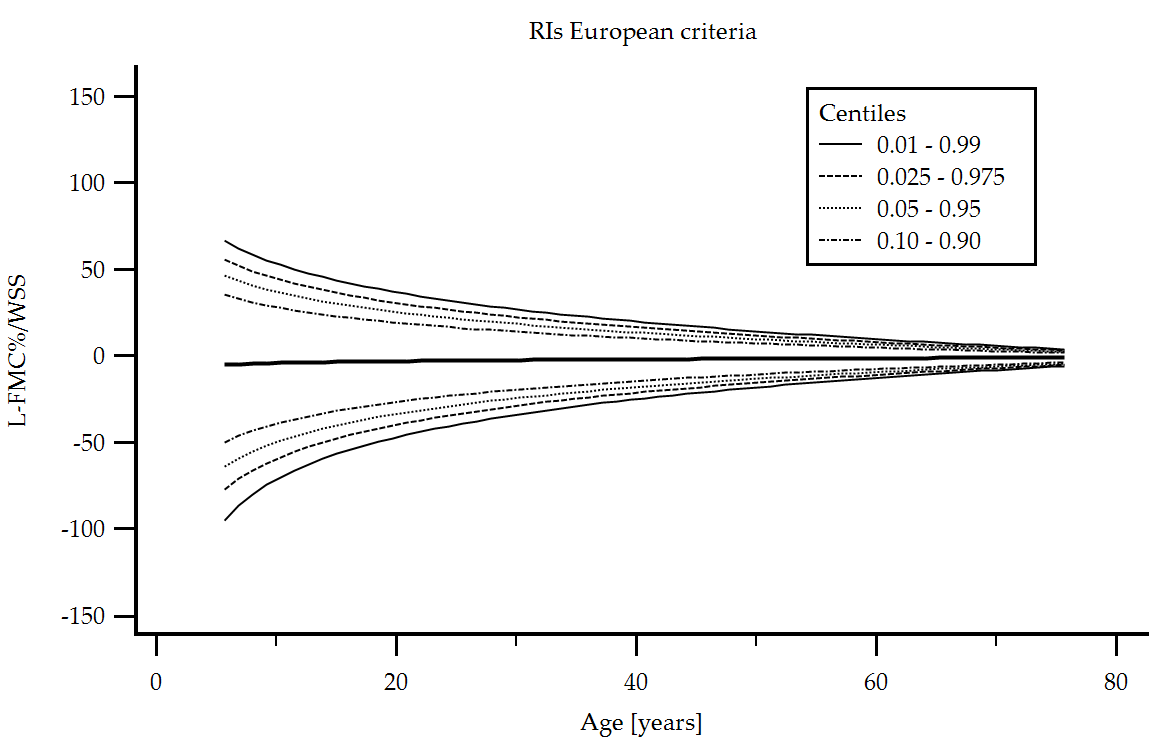


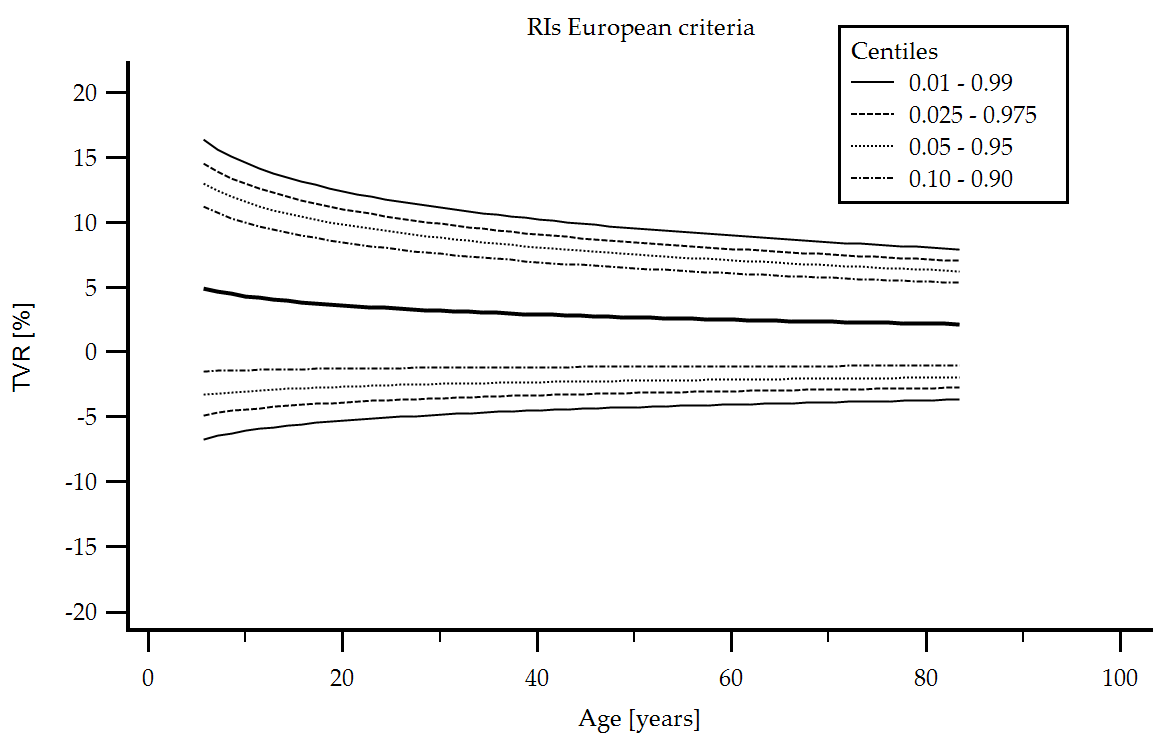


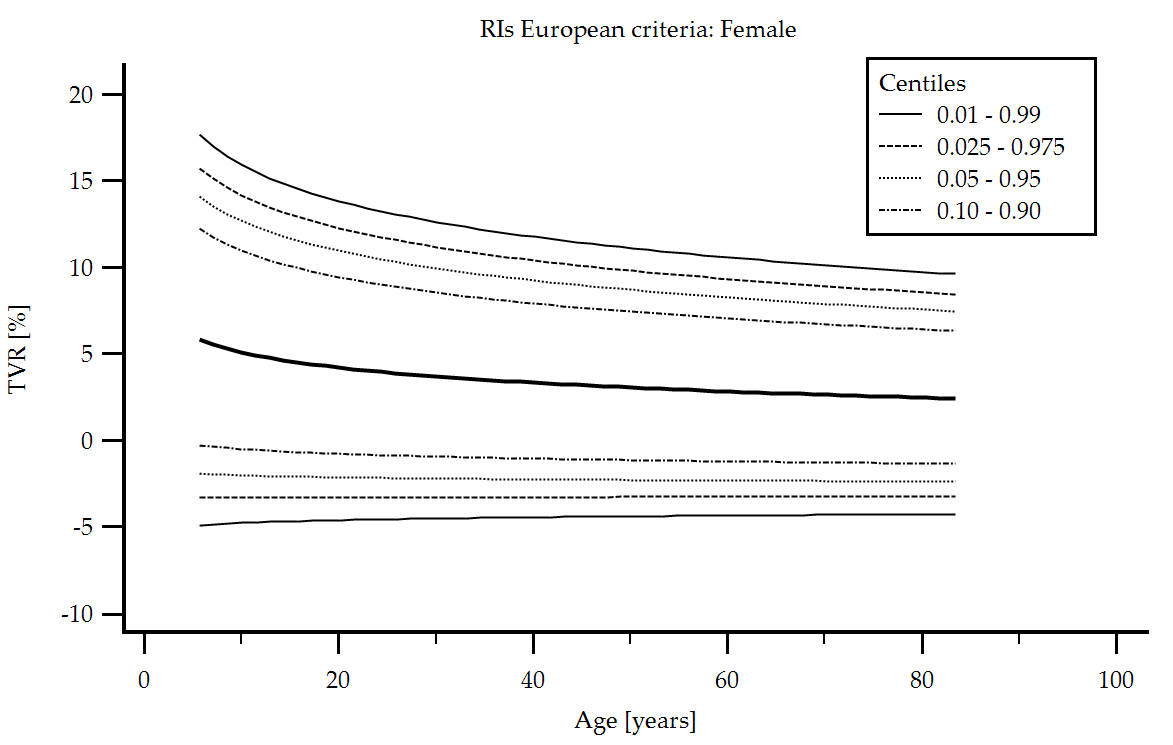


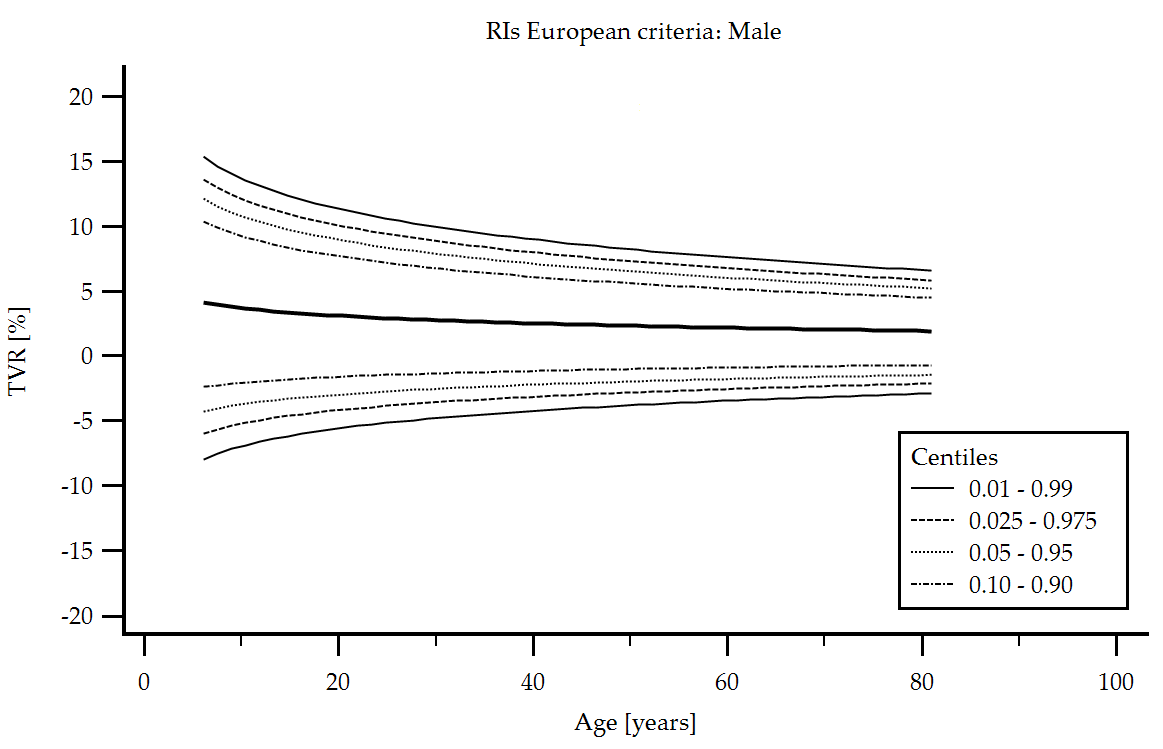


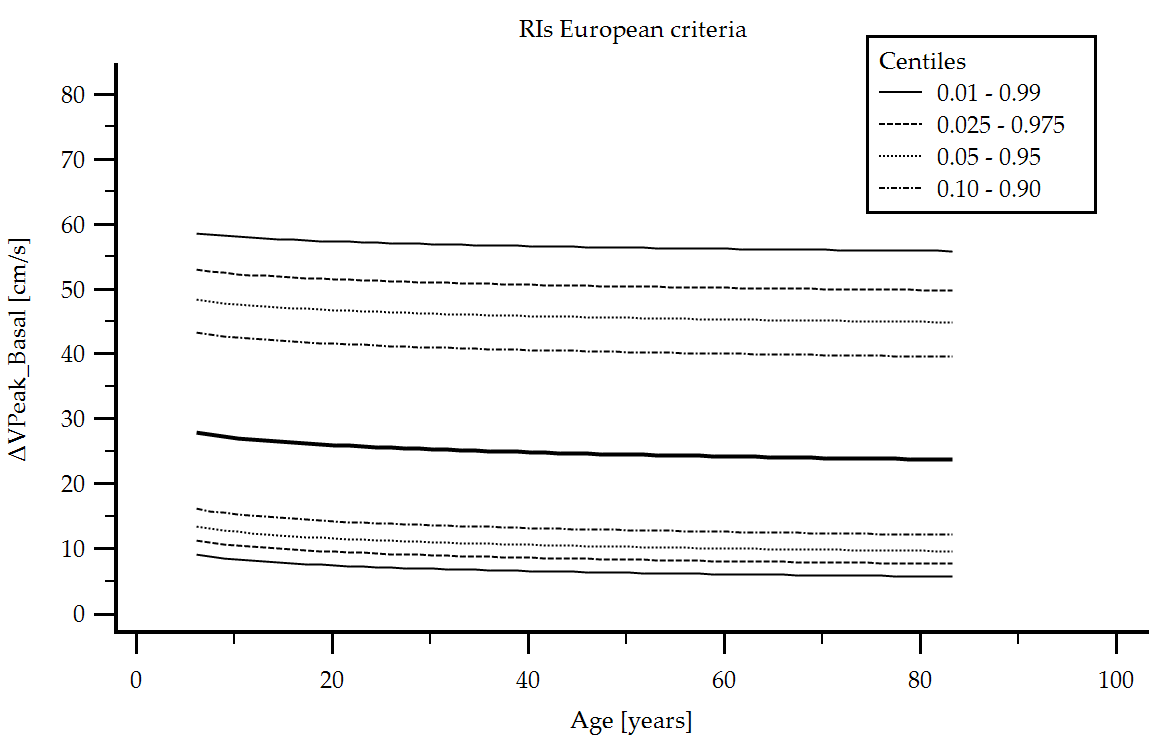


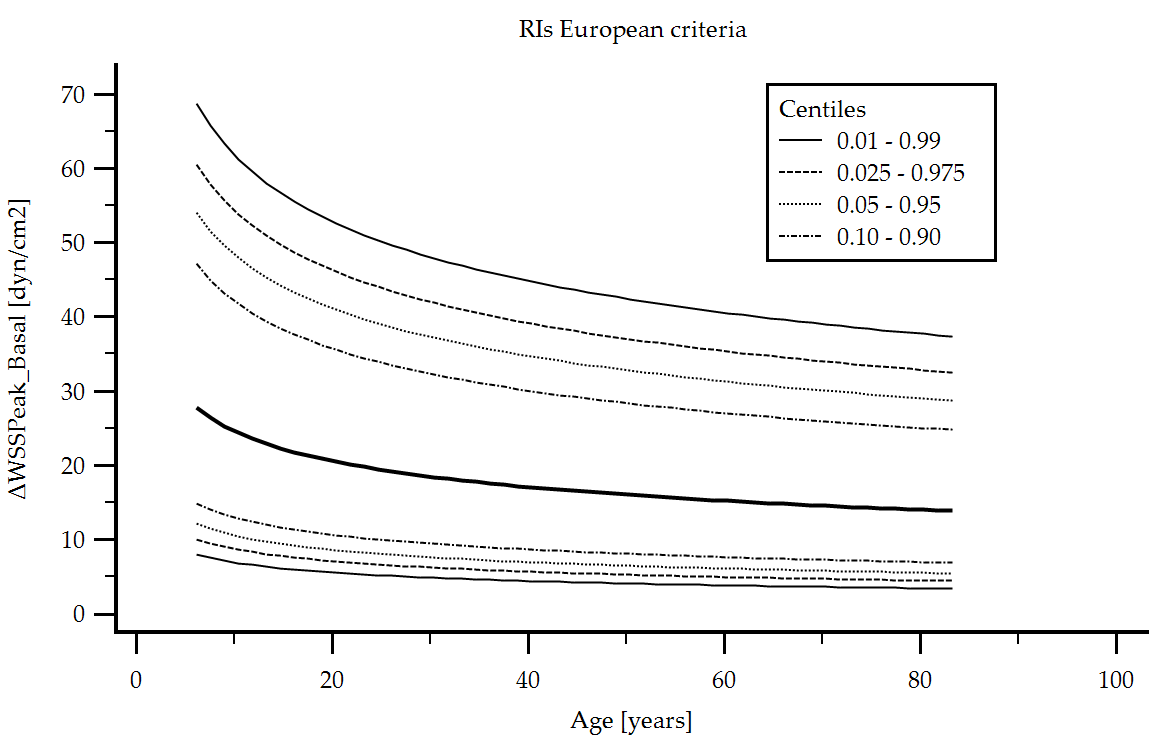

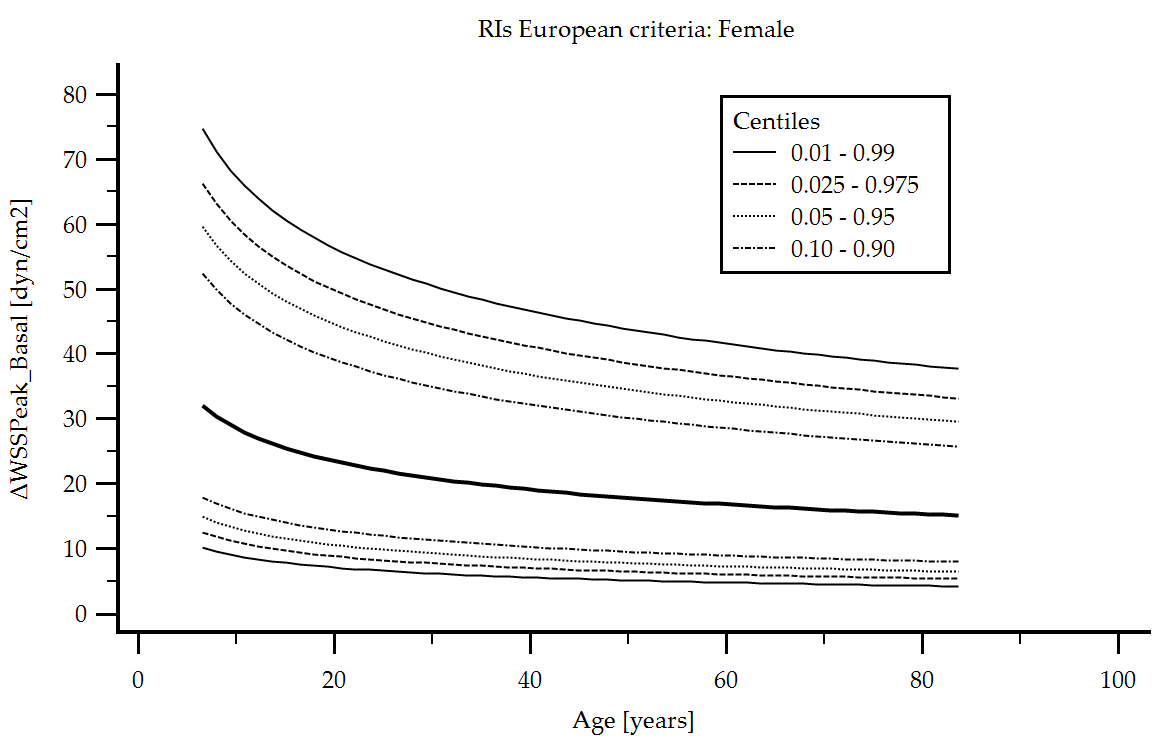


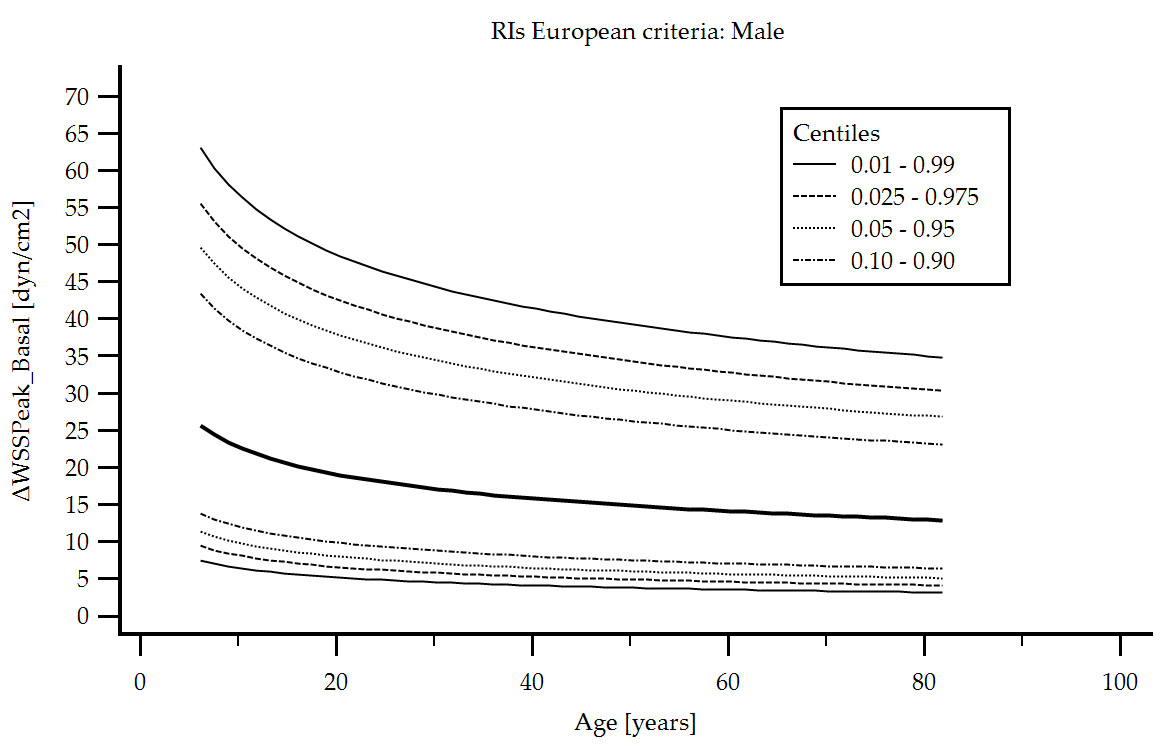


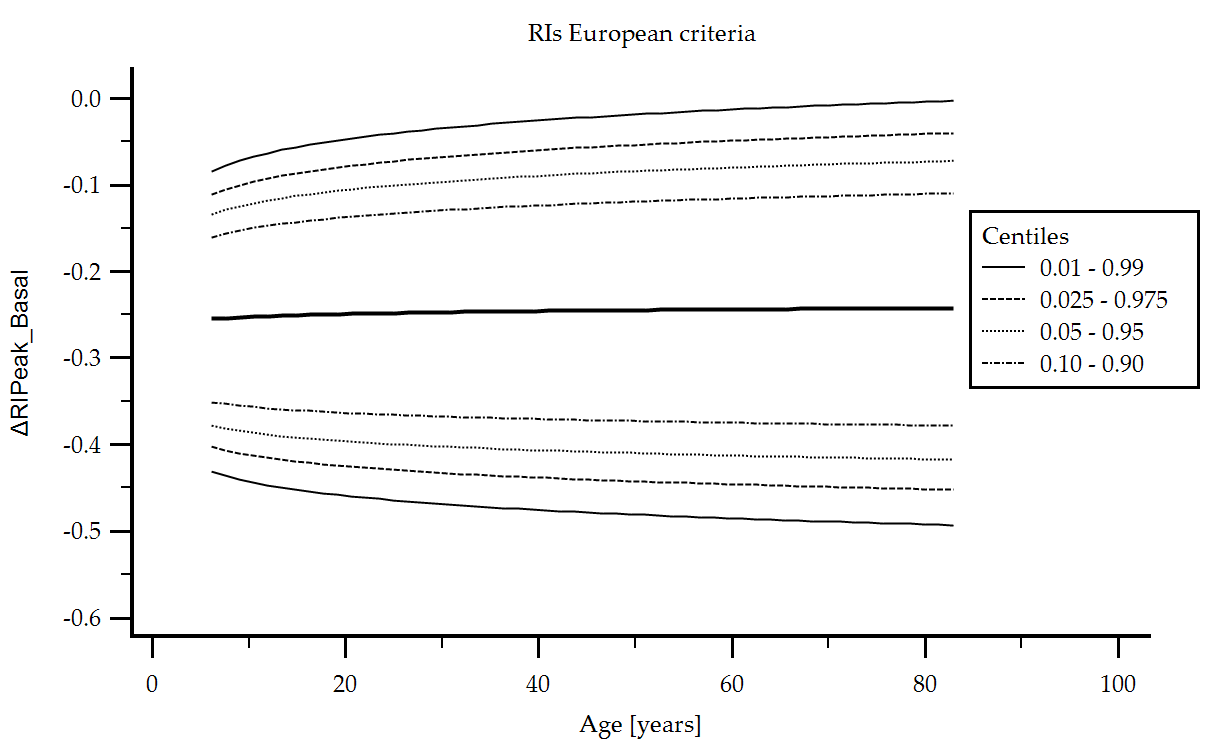


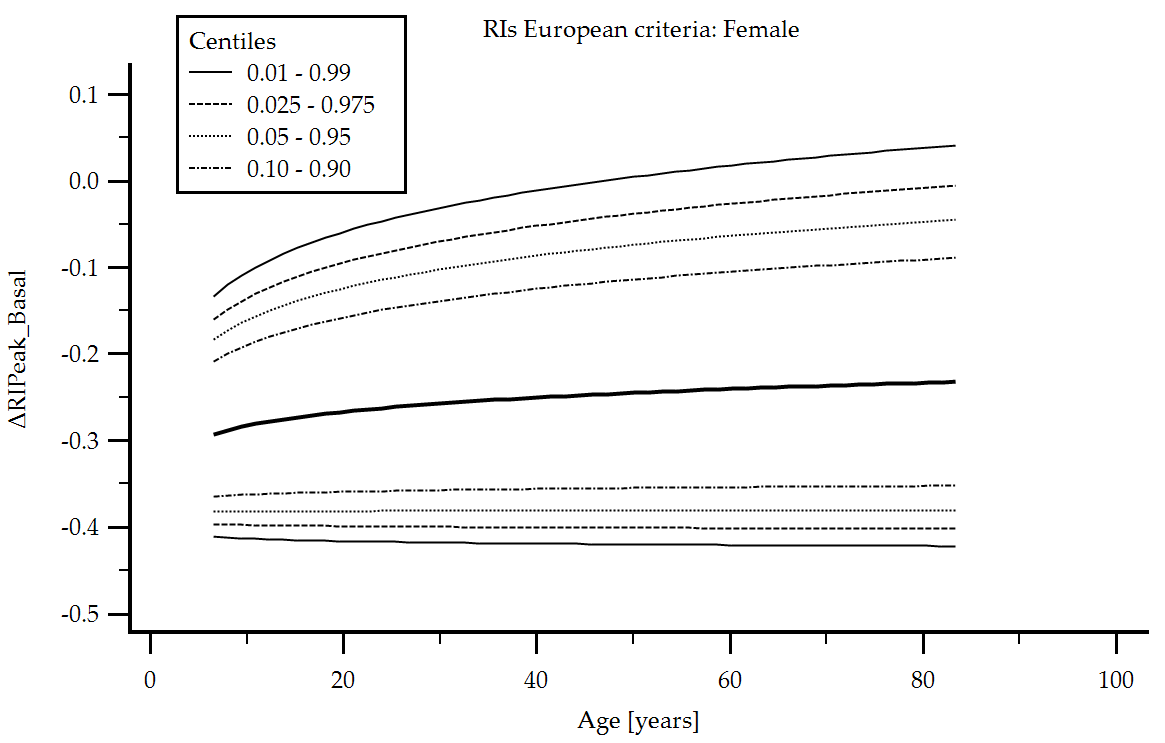


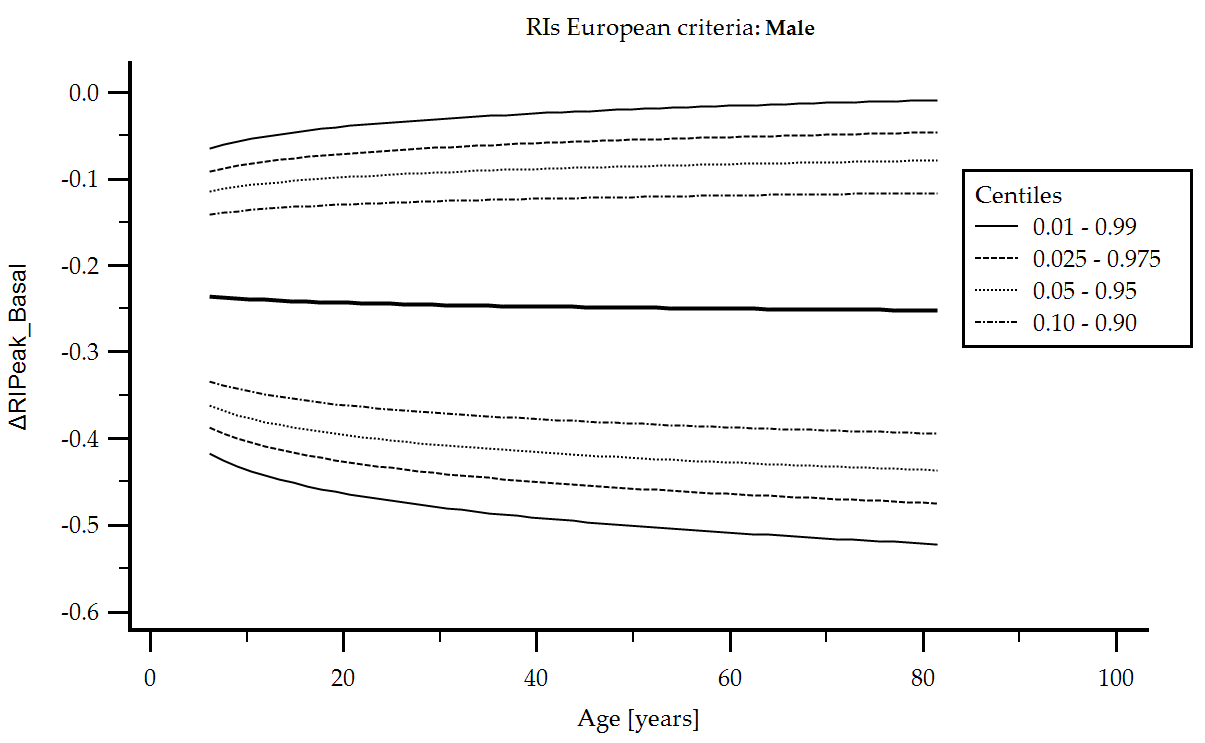


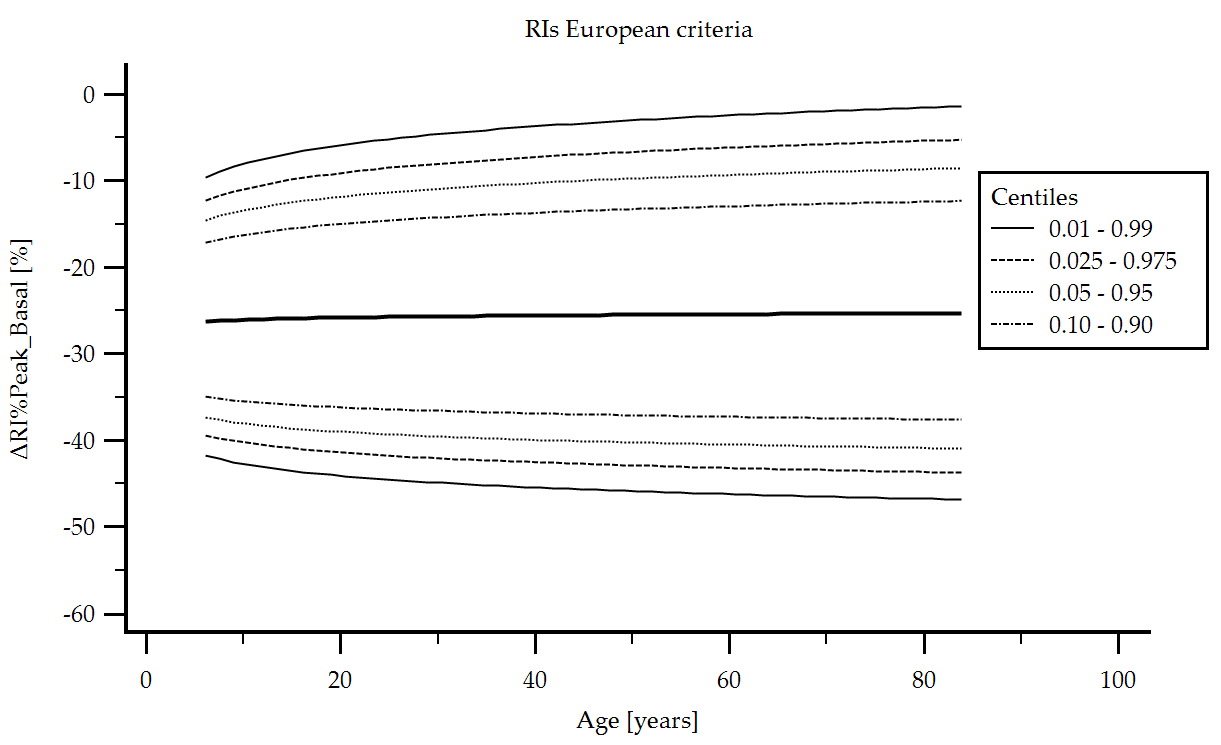


-------------------------------------------------------------------------------------------------------------------

**Figure S2. Age-related profiles for vascular reactivity indexes: ´HUNT-FIT criteria´**


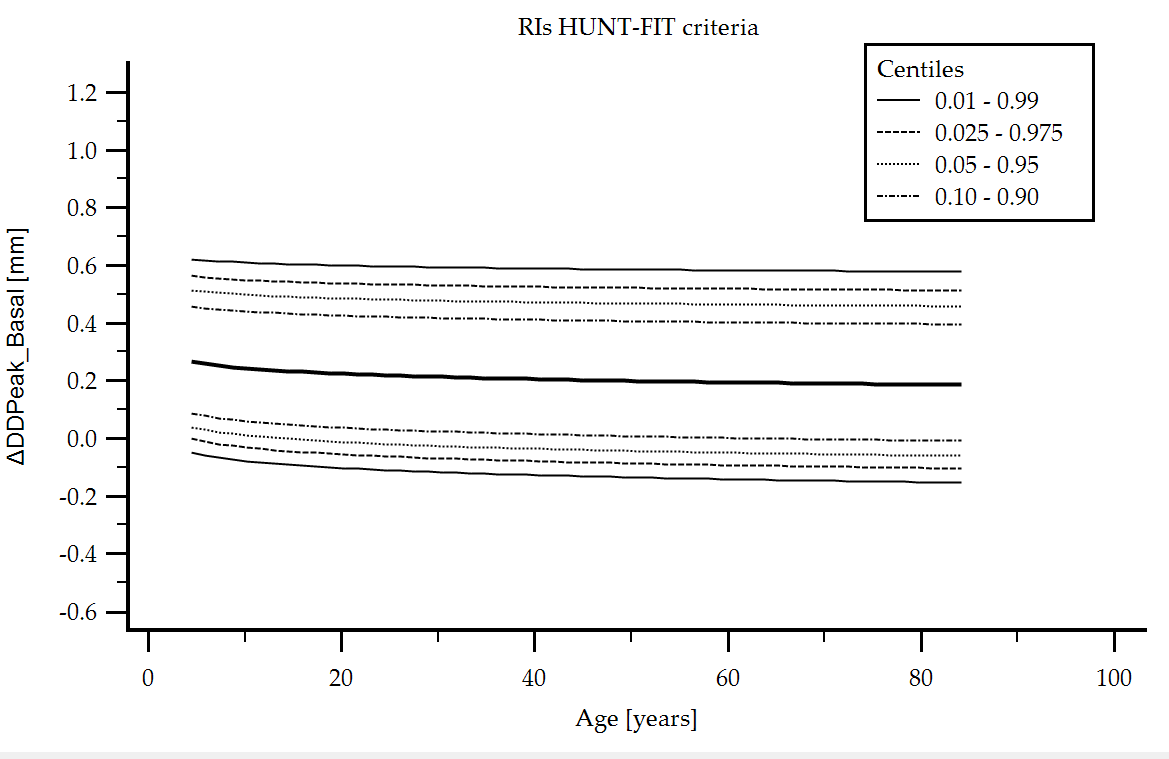


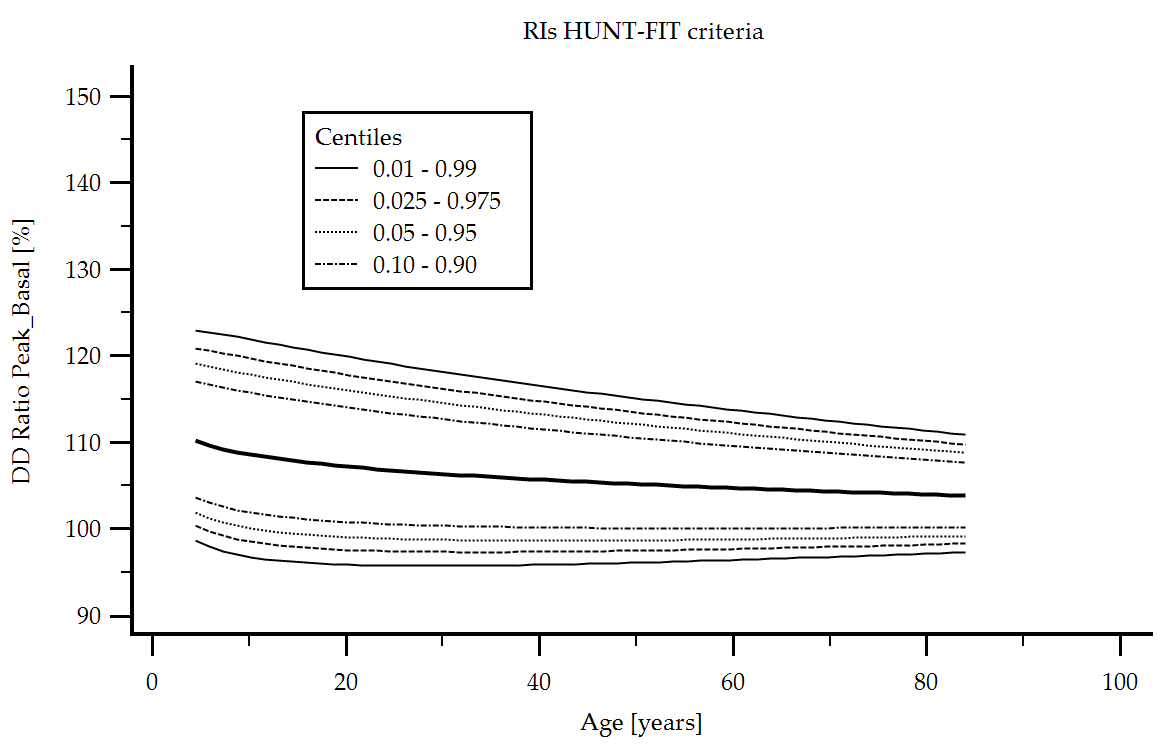


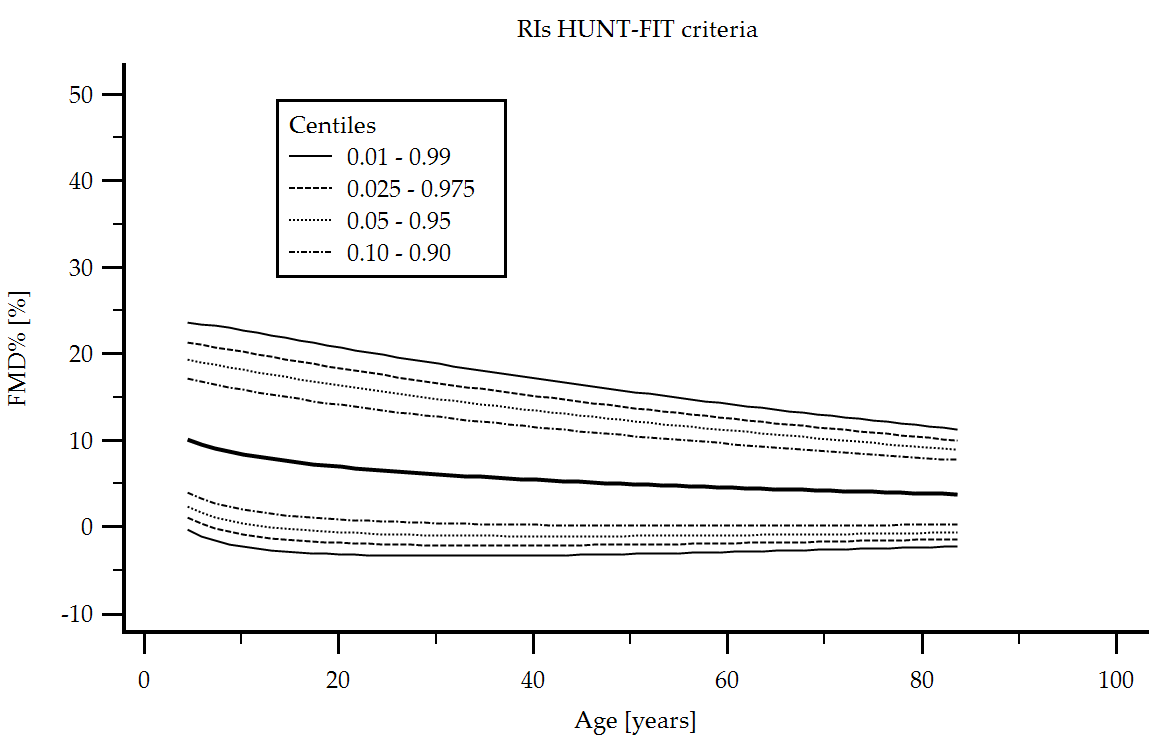


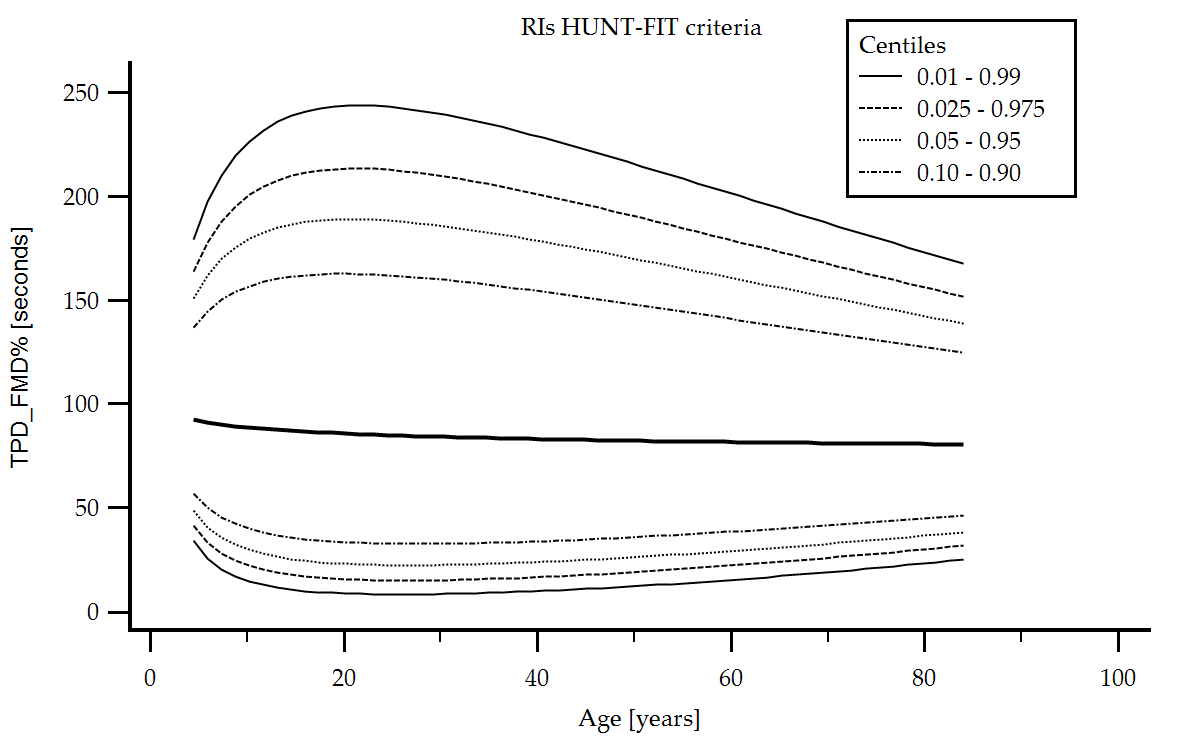


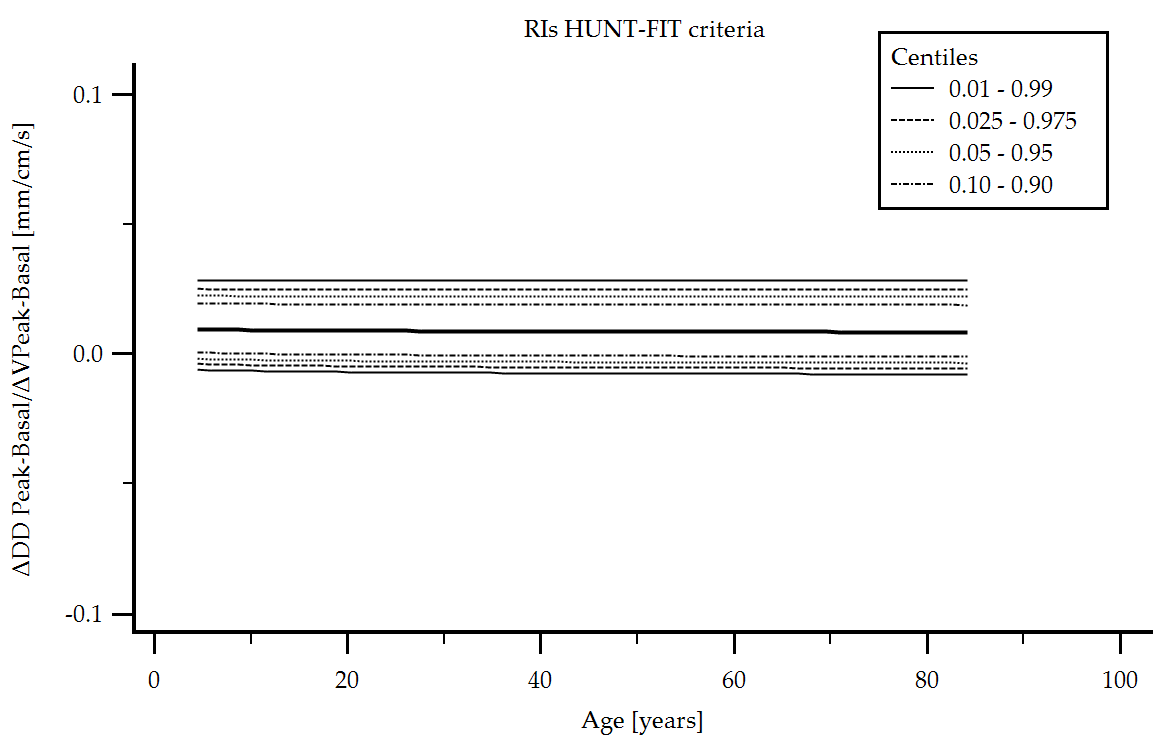


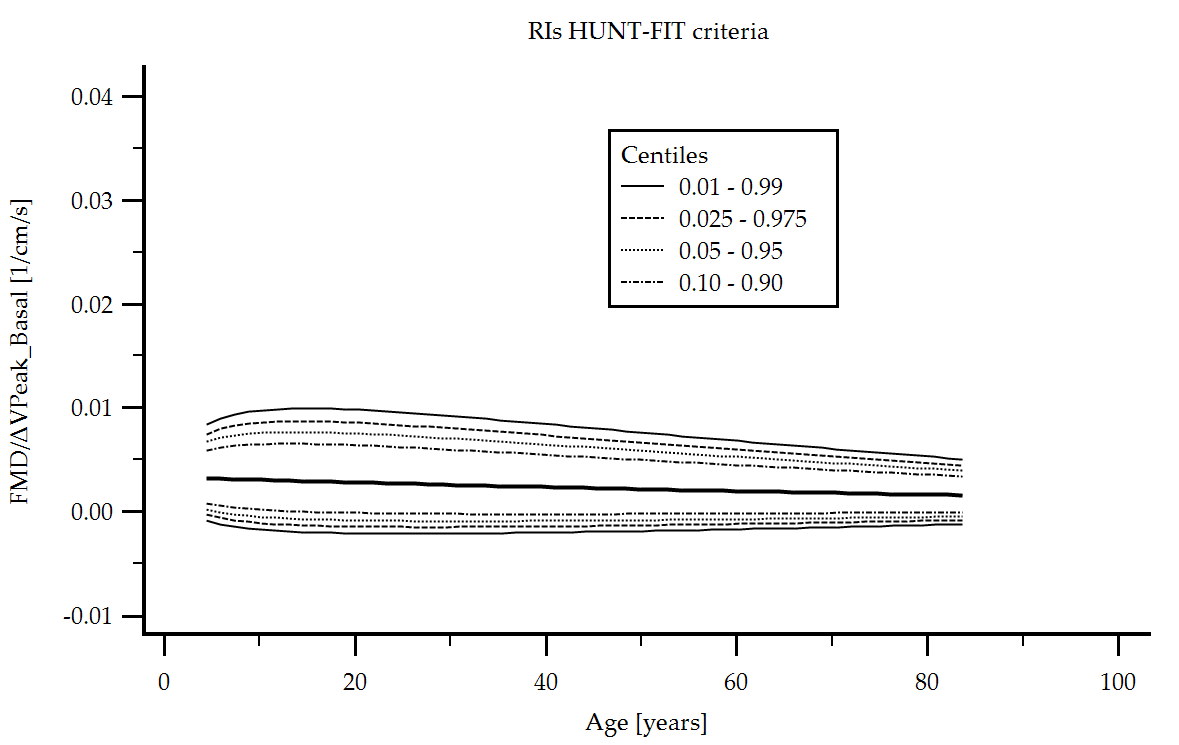


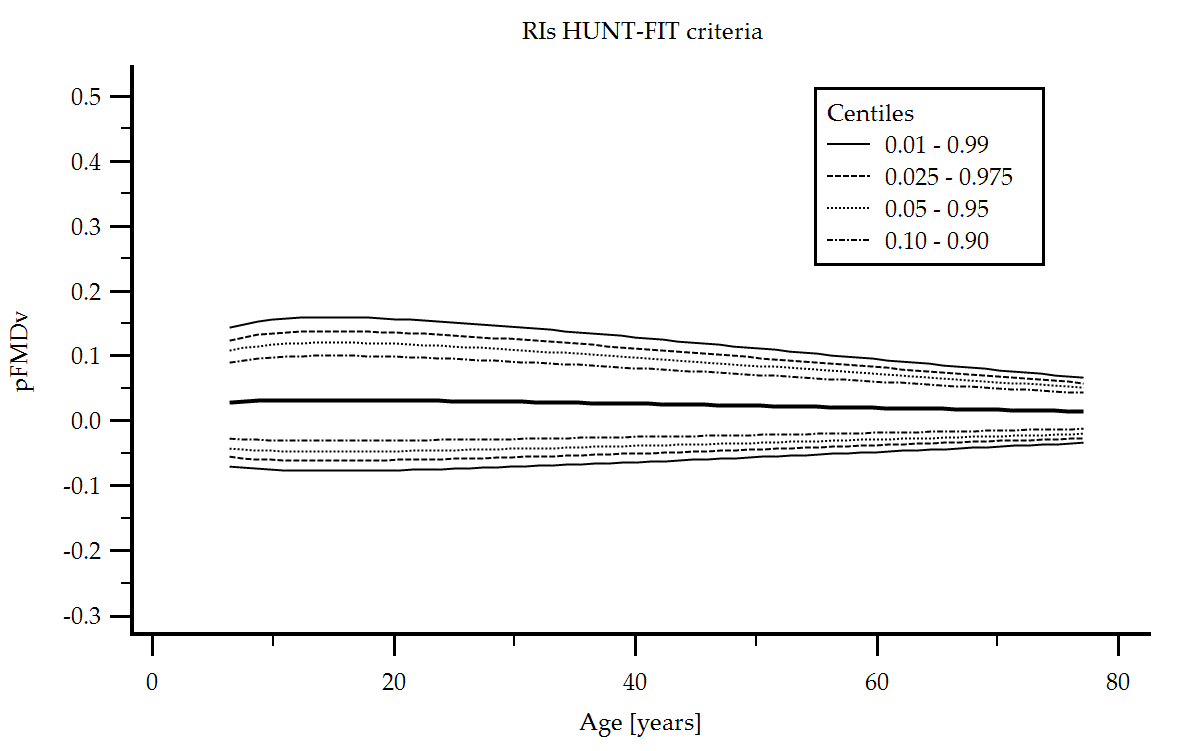


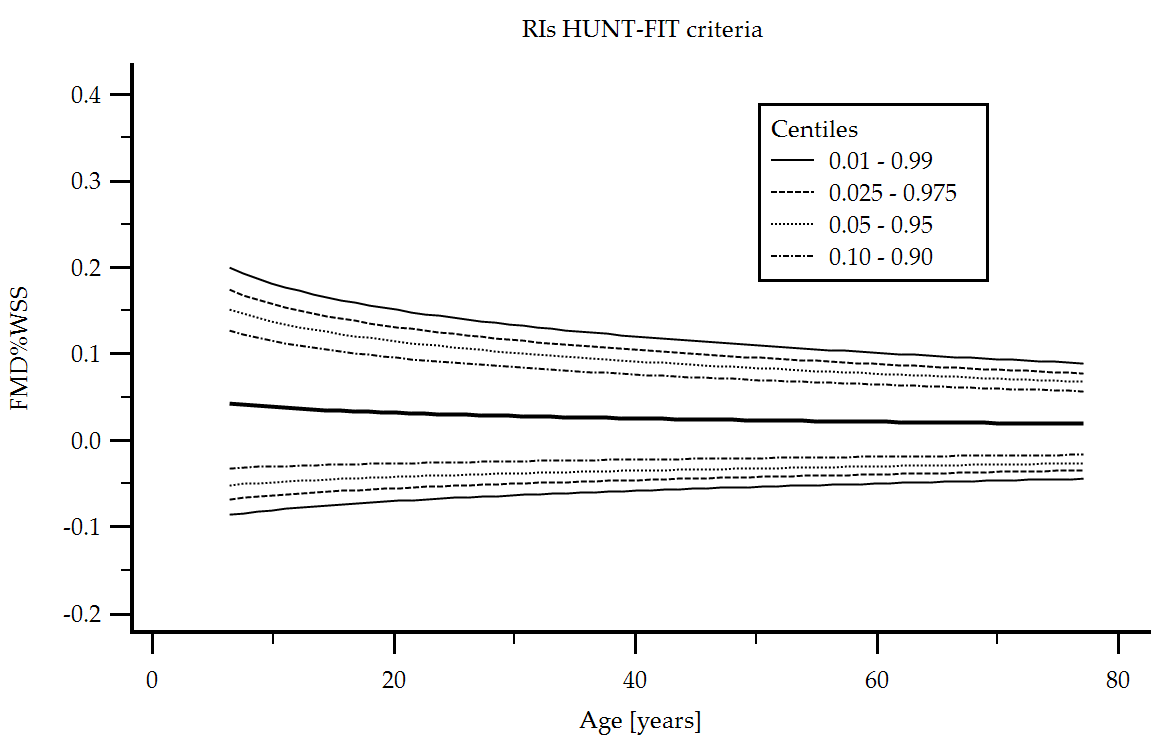


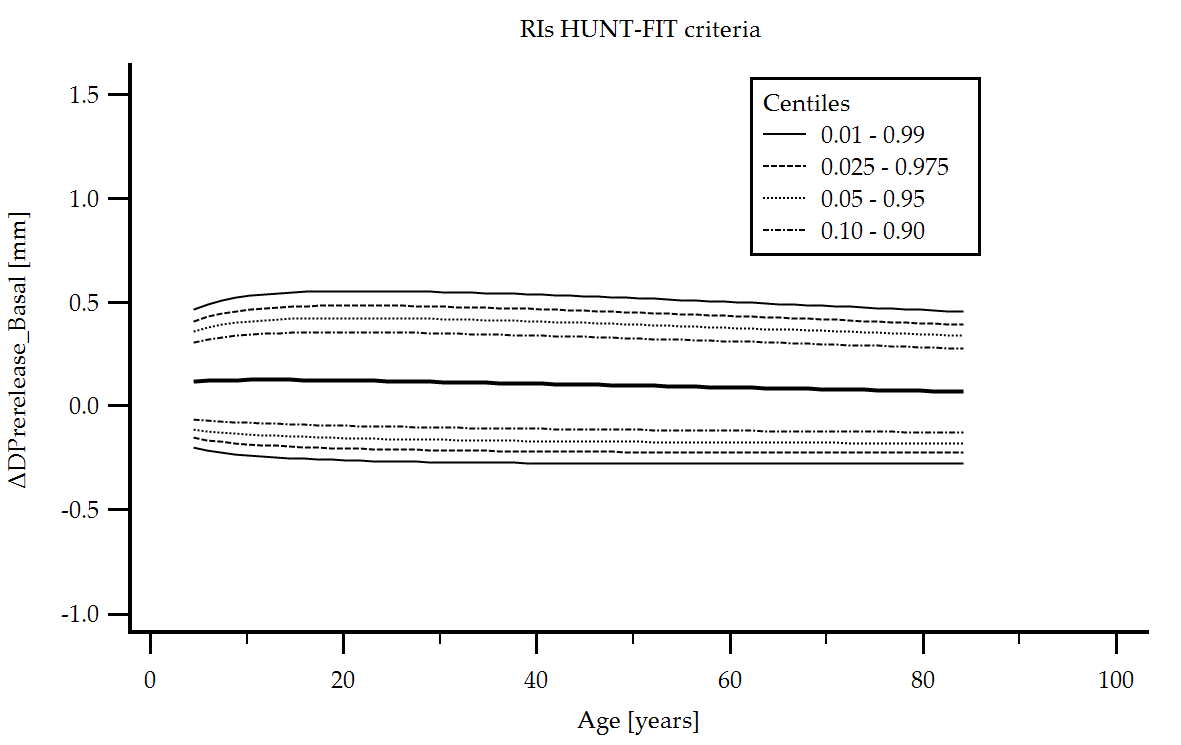


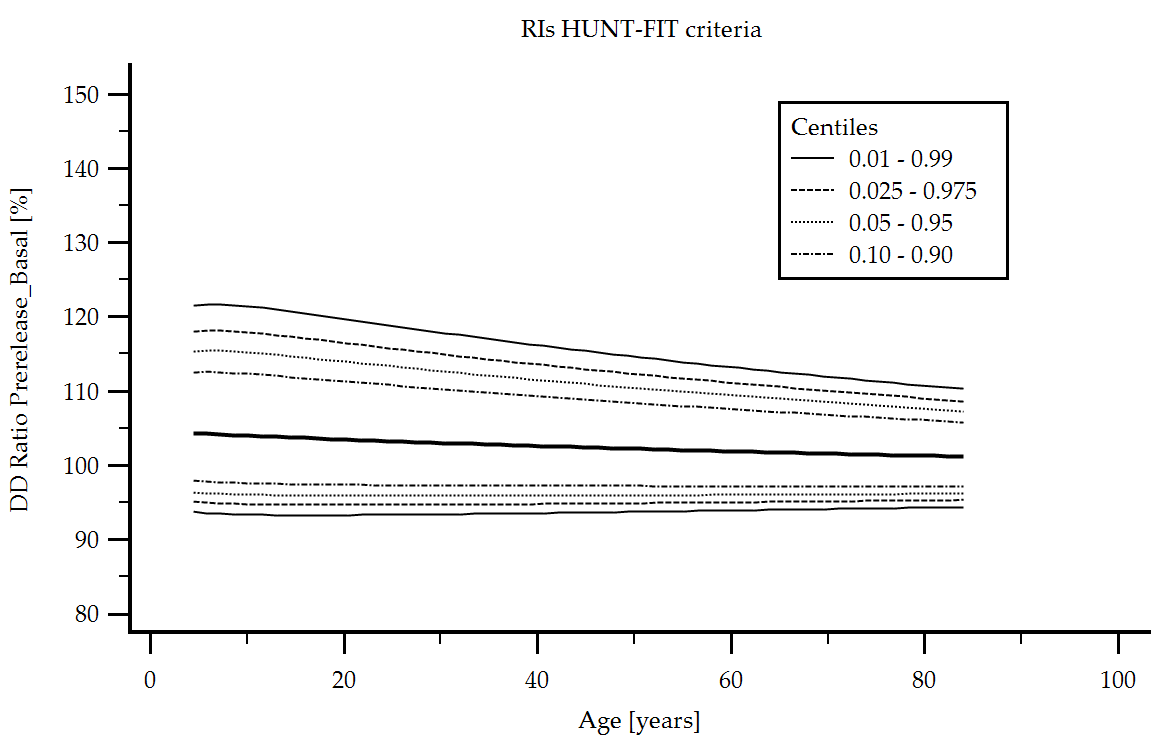


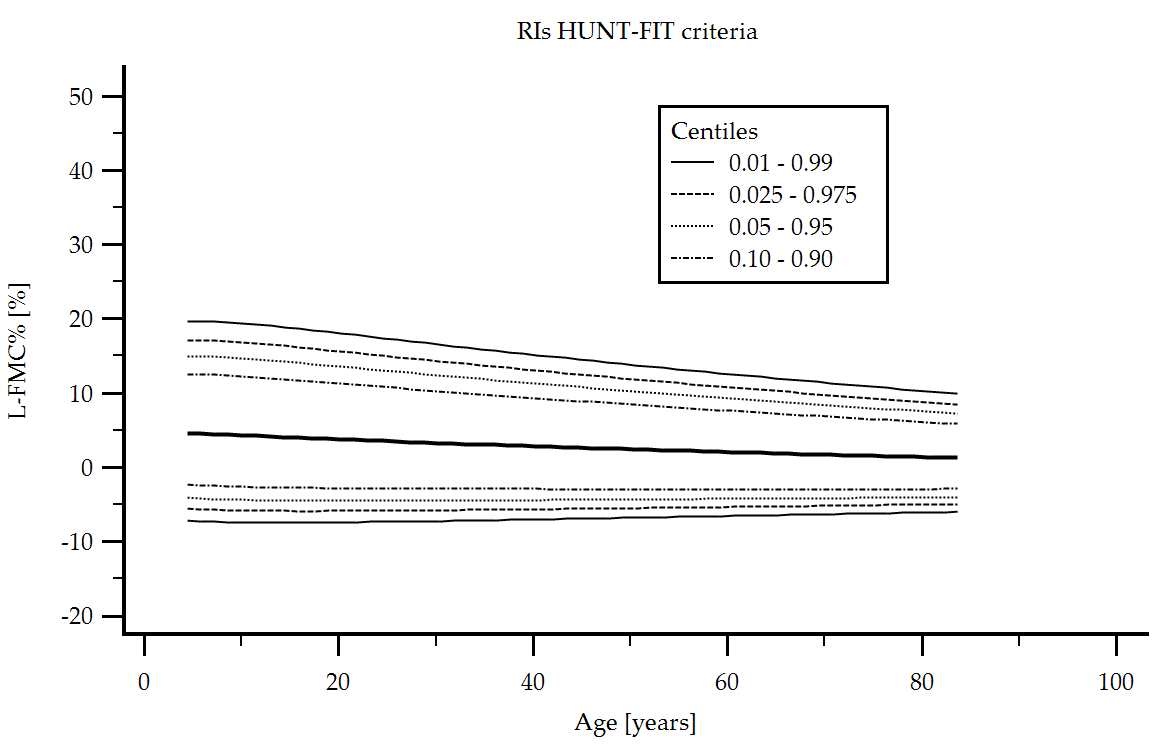

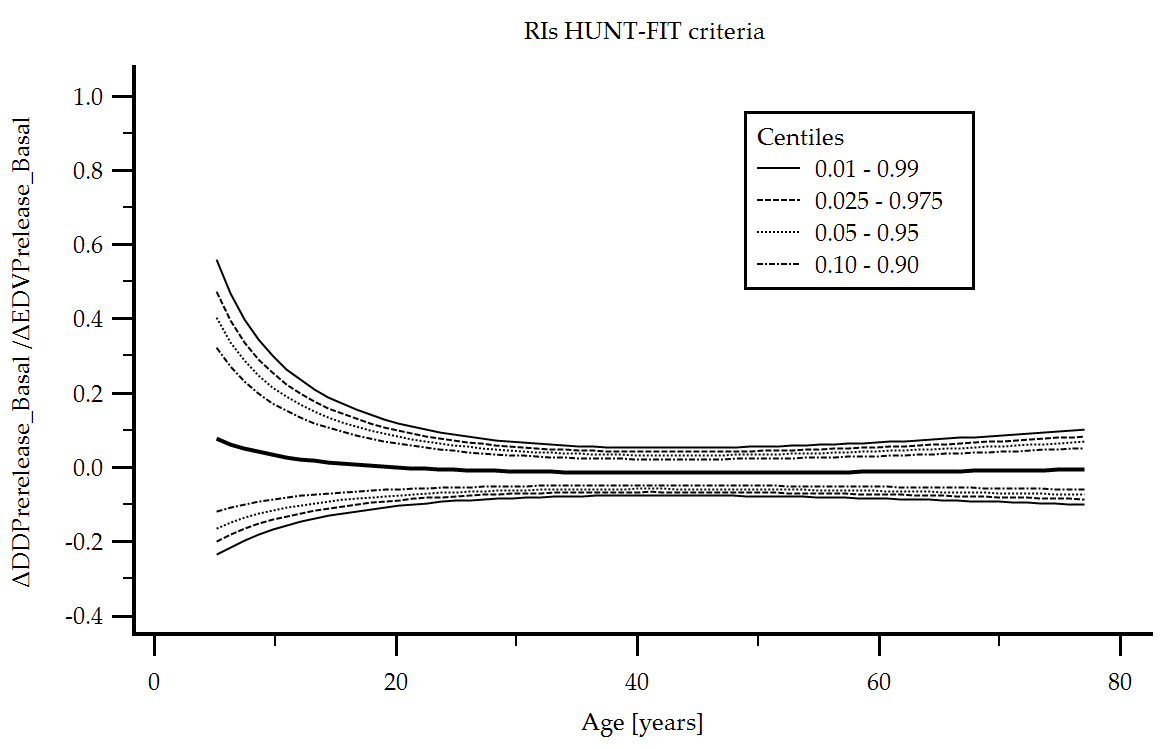


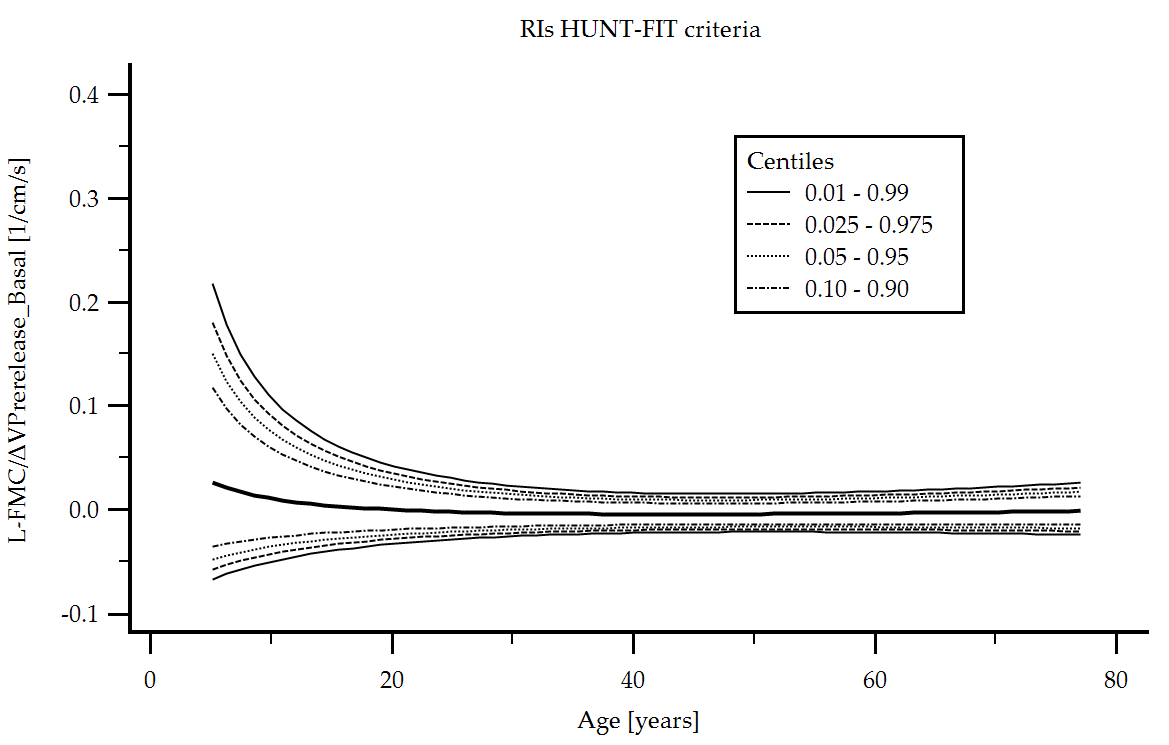


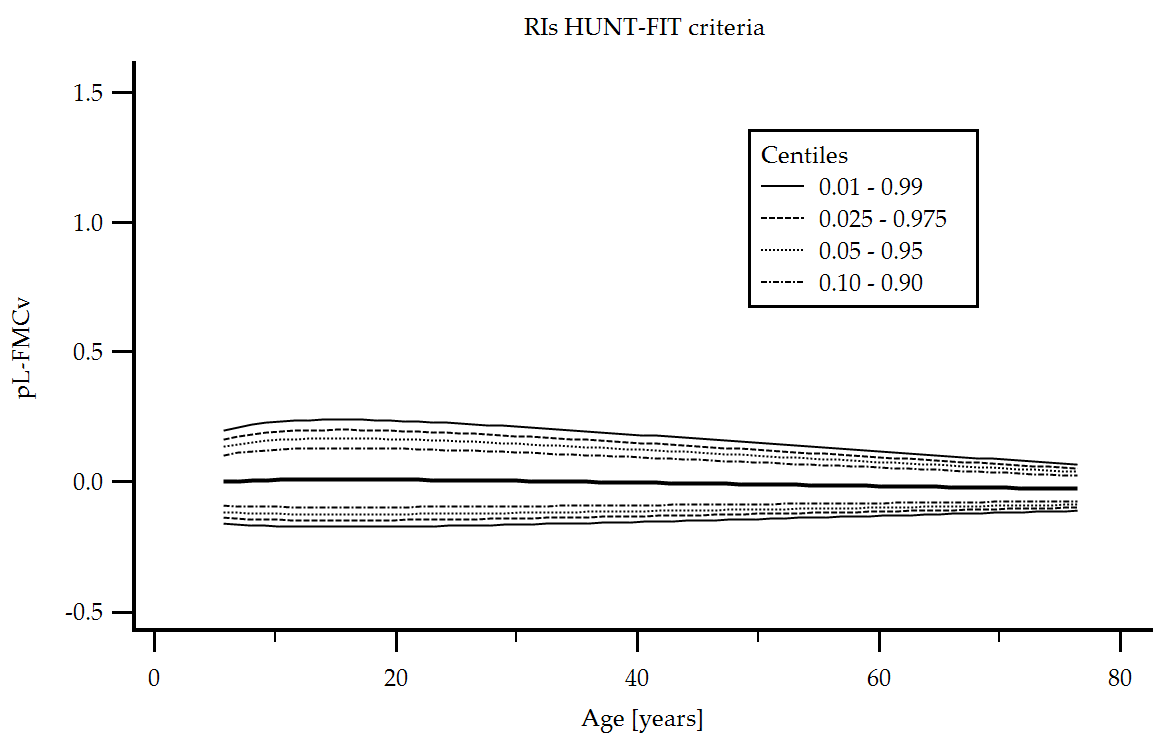


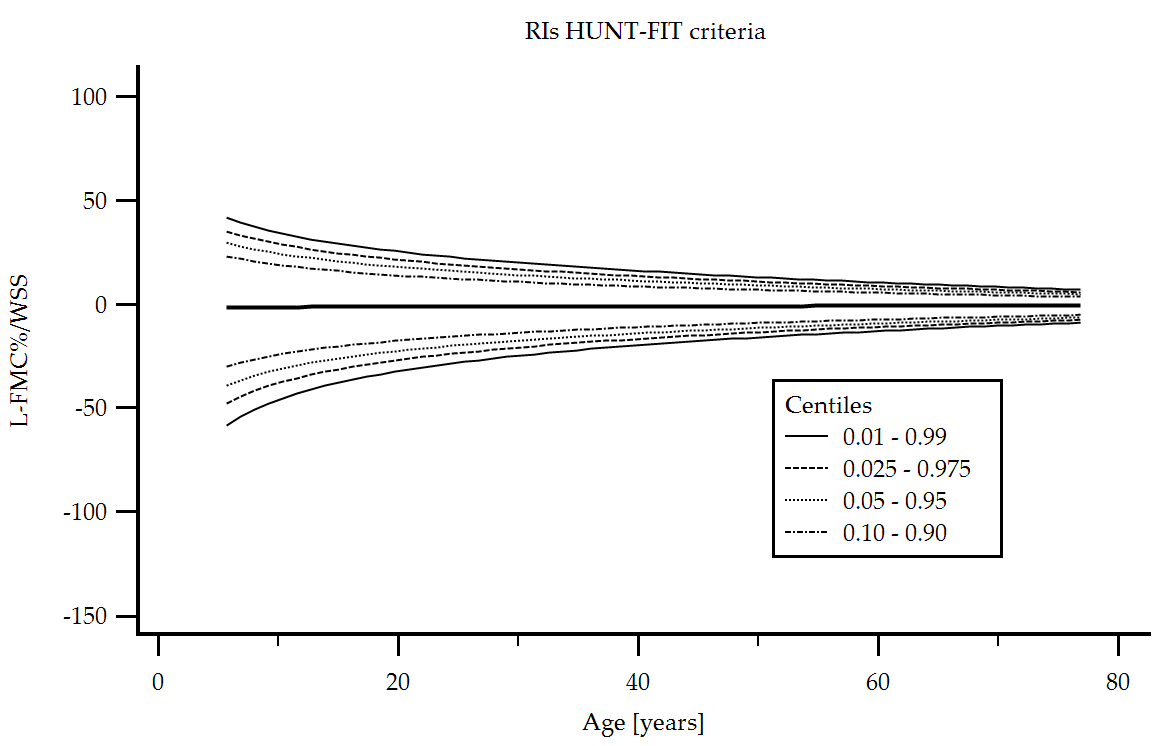


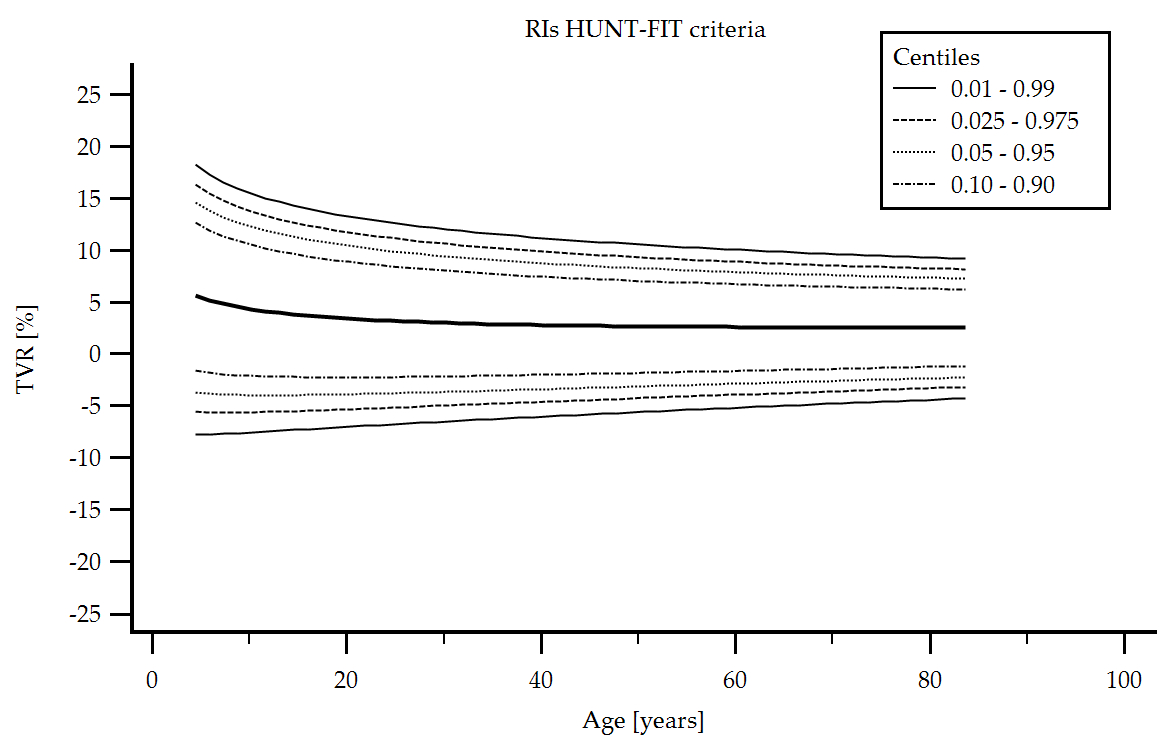


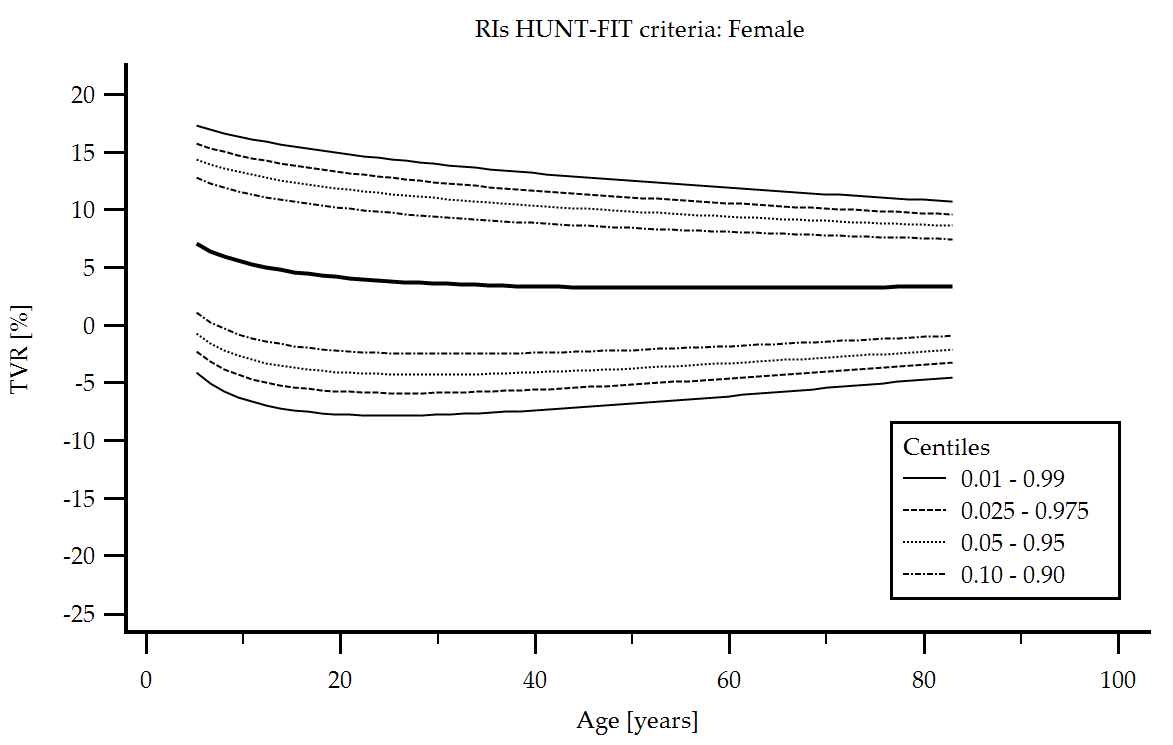


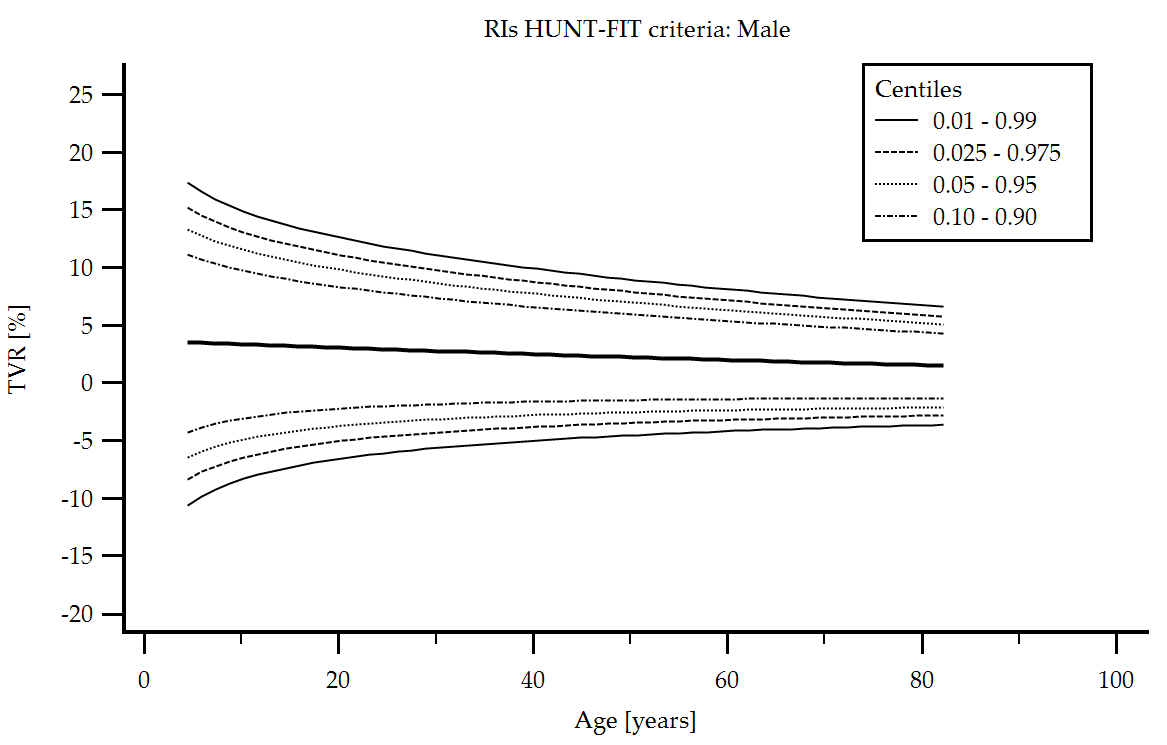


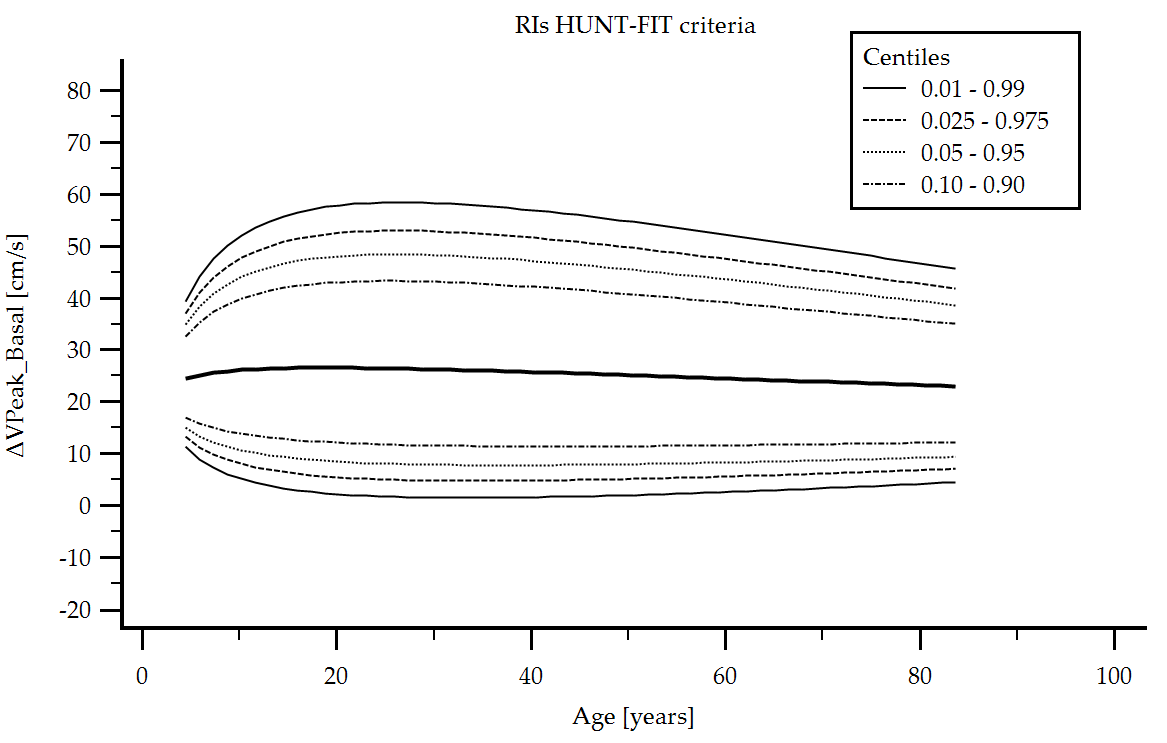


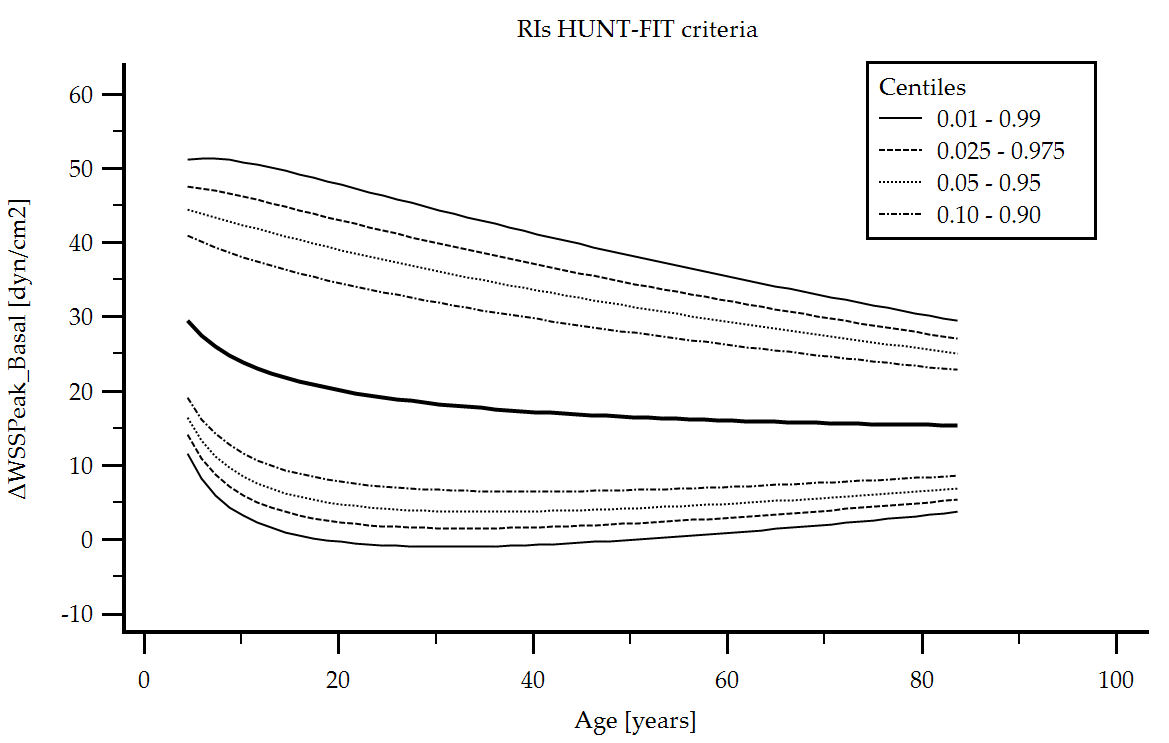


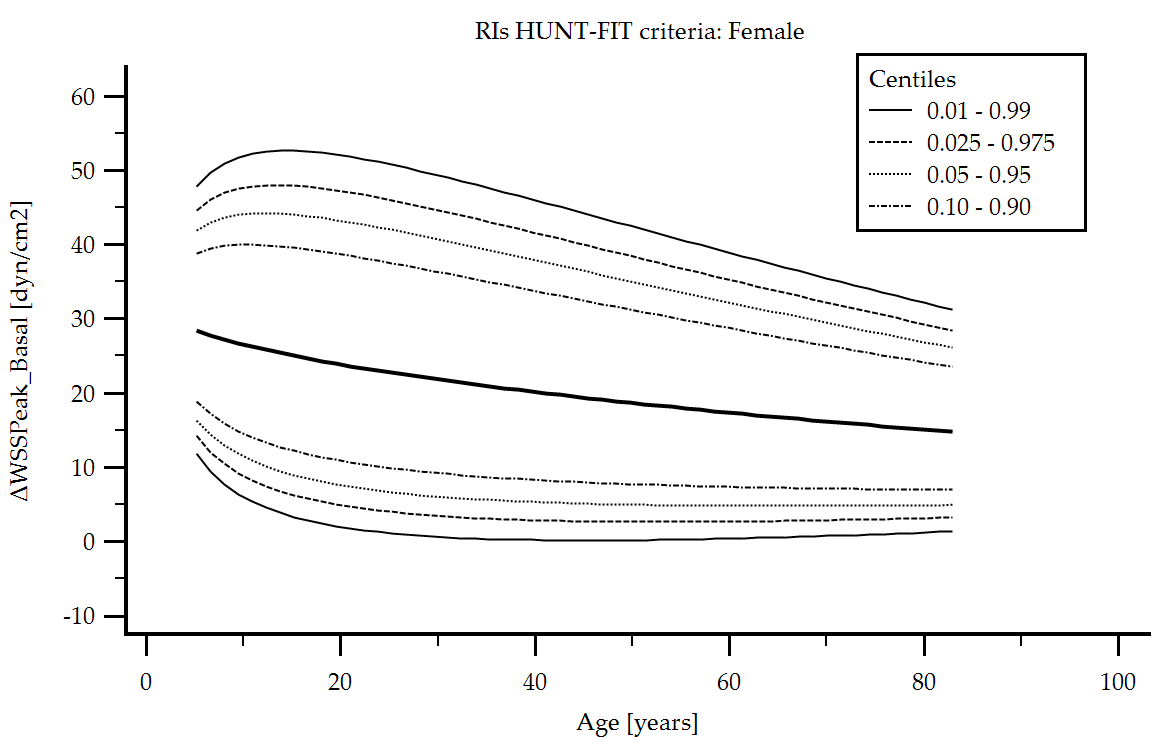


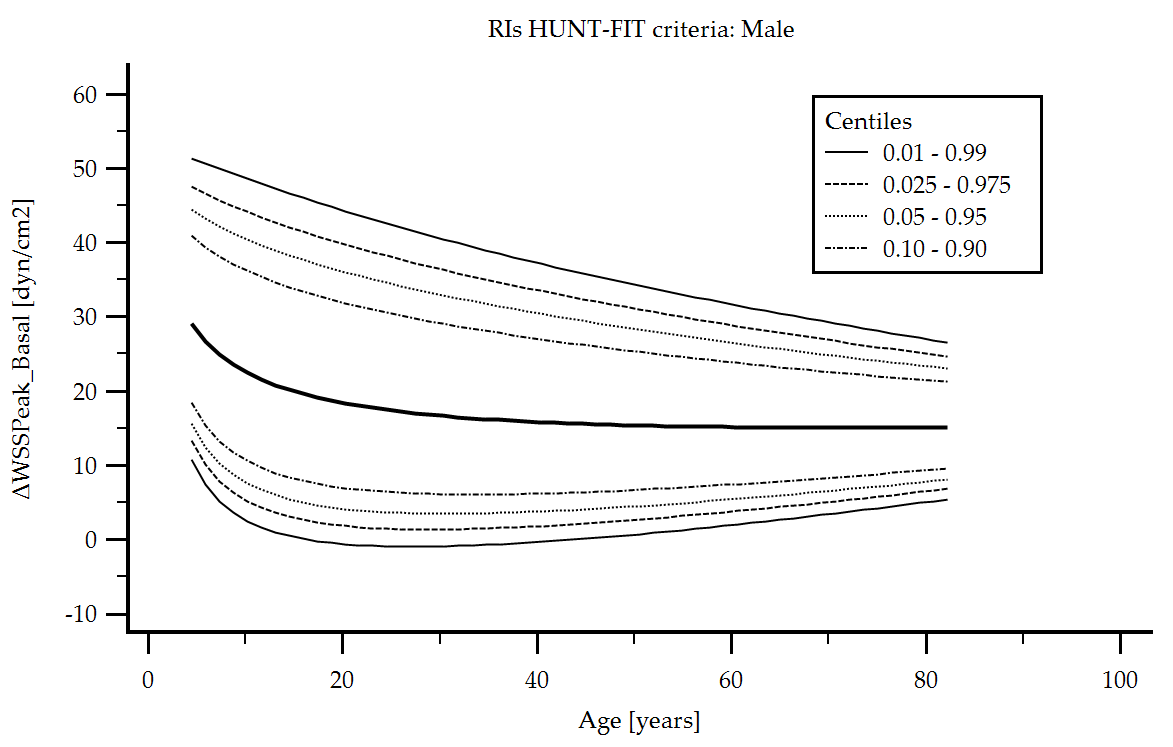


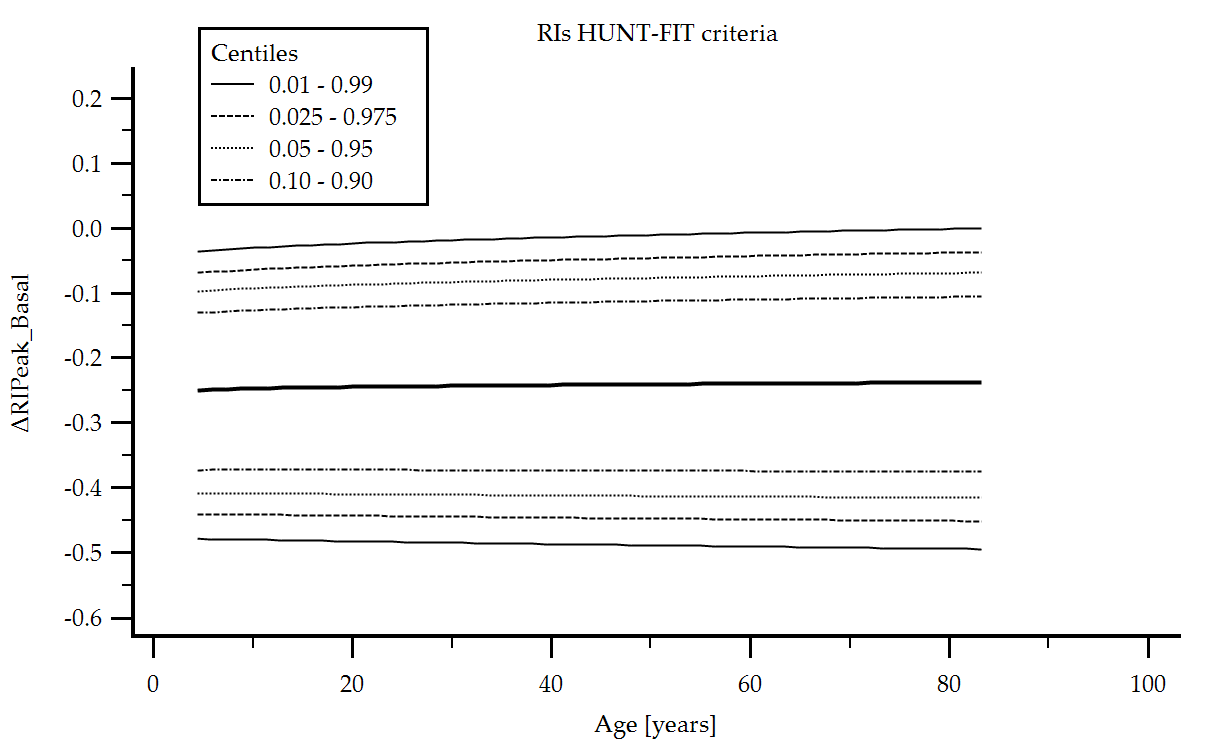


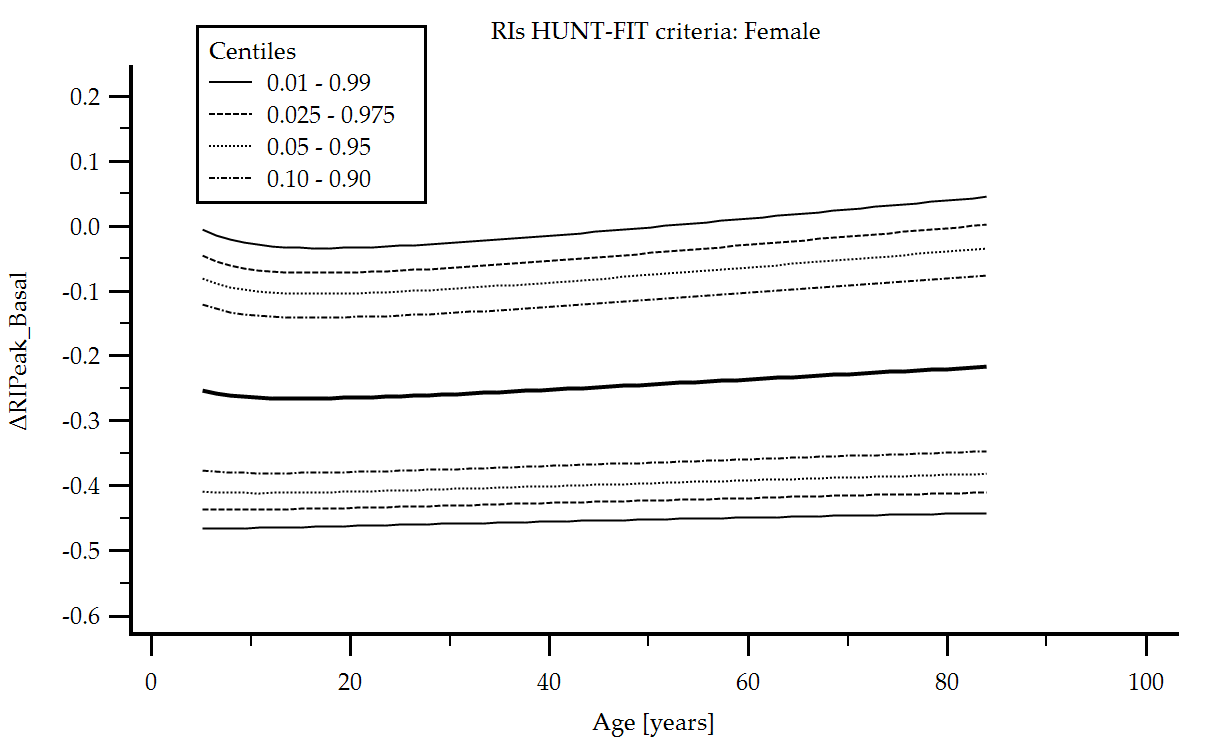


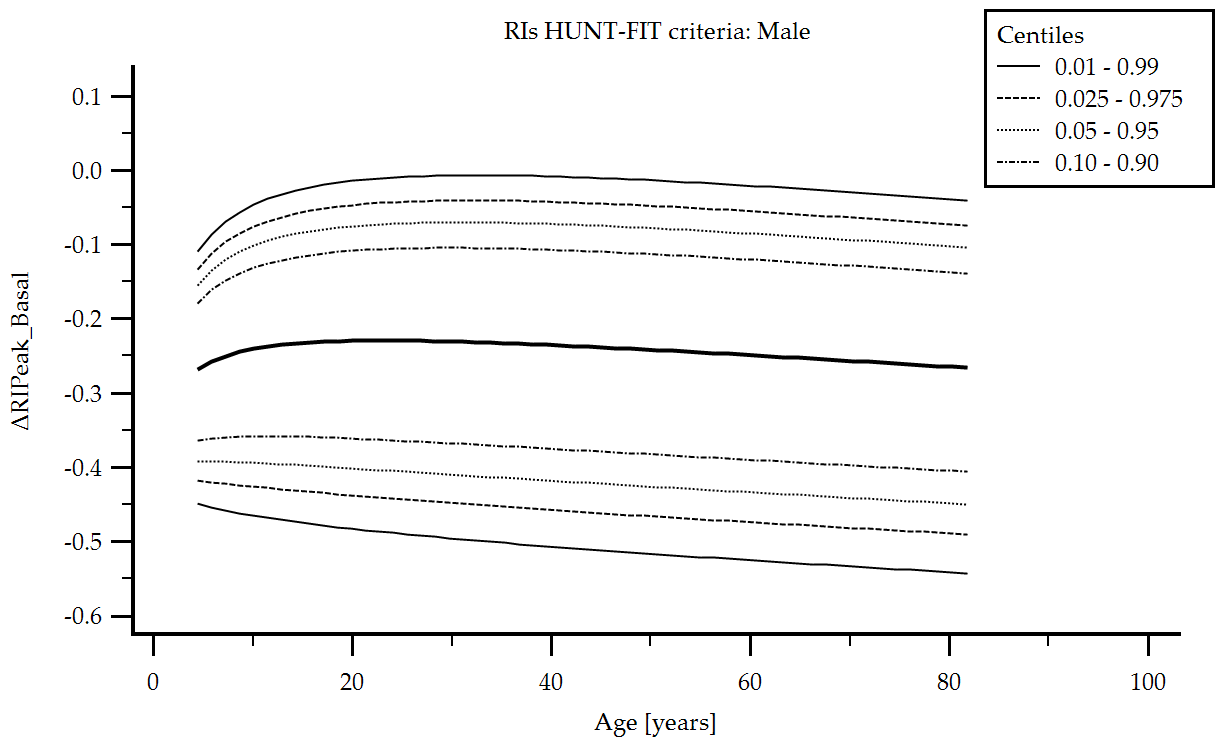


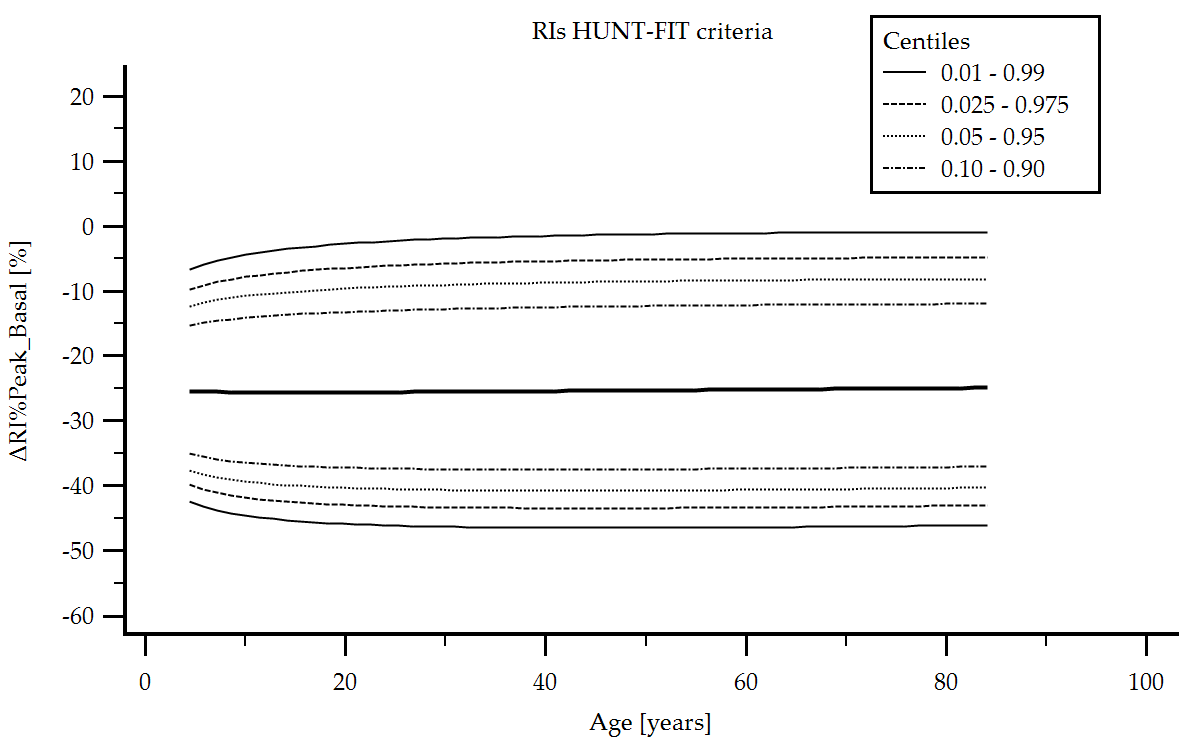

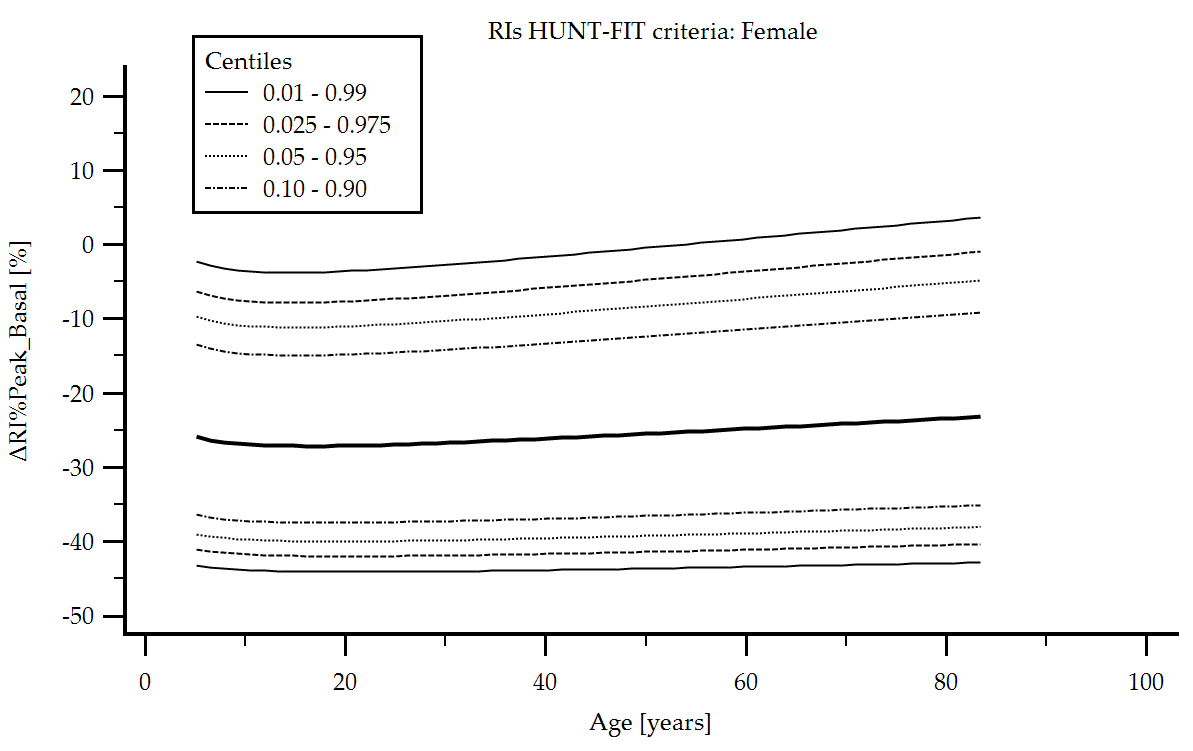


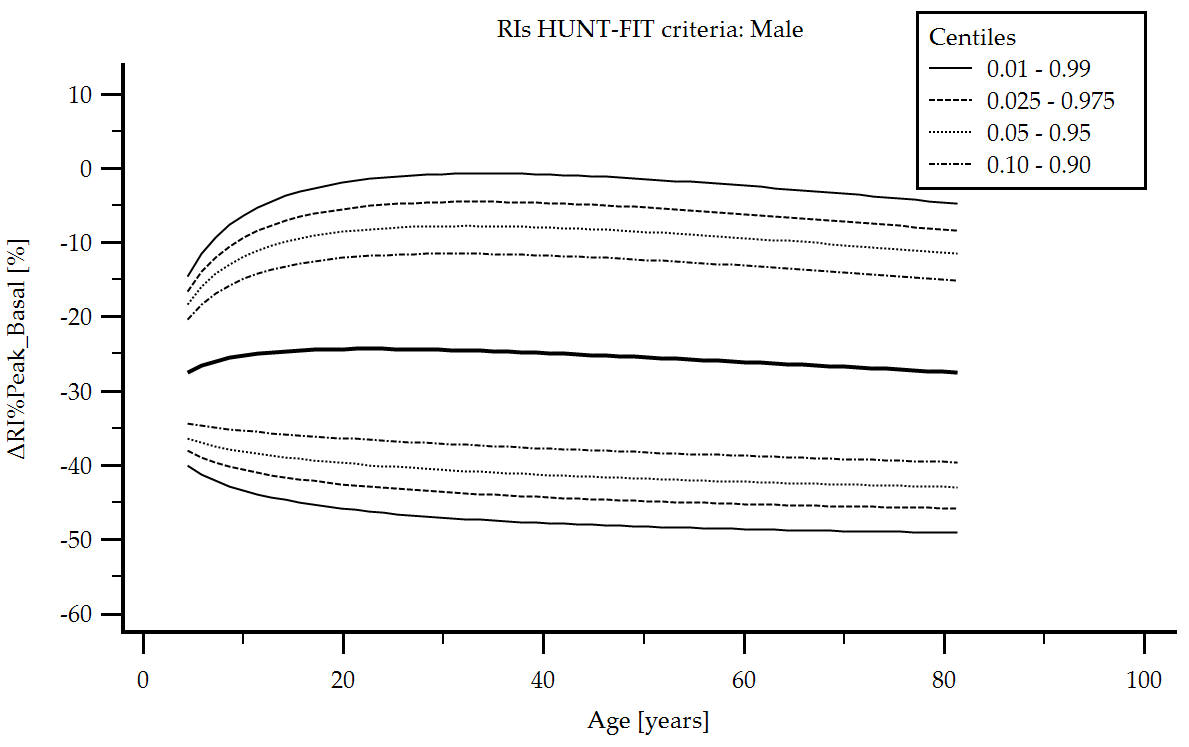

Supplement: S2 File — Figure S1. Age-related profiles for vascular reactivity indexes: ´European criteria. Figure S2. Age-related profiles for vascular reactivity indexes: ´HUNT.FIT criteria. (DOCX) [file pone.0254869.s002.docx]
